# Multidimensional strategy enables scalable metabolome diversity in microbial fermentations

**DOI:** 10.1038/s41598-026-37748-9

**Published:** 2026-01-29

**Authors:** Anton Lindig, Makram Fataeri, Georg Hubmann, Stephan Lütz

**Affiliations:** 1 Chair for Bioprocess Engineering, Department of Biochemical and Chemical Engineering, TU Dortmund University, Emil-Figge-Straße 66, 44227 Dortmund, Germany; 2 Bioprocess Engineering, Faculty of Technology, Bielefeld University, Universität straße 25, 33615 Bielefeld, Germany

**Keywords:** Natural product discovery, Multidimensional optimization, Microbioreactor system, Stirred tank bioreactor, *Streptomyces*, Molecular networking, Biotechnology, Chemical engineering, Applied microbiology

## Abstract

Natural products (NPs) are critical sources of new drug leads, but their biosynthesis is highly sensitive to bioprocess parameters and environmental conditions, making NP discovery especially susceptible to challenges in achieving robust scalability and reproducibility of the metabolome across various cultivation systems. Here, we identified key factors that improved the metabolic footprint overlap of *Streptomyces griseochromogenes* across three cultivation systems, i.e. baffled shake flasks (BSF), 48 flower plates (48 FP), and a stirred tank bioreactor (STR), by 50 %. Using the classical scale-up criterion of constant oxygen availability, the metabolic footprints were found to differ considerably, with only an 18 % overlap in mass feature (MF) number. Efforts to improve overlap by varying ethanol concentration in culture medium and oxygen availability resulted in an 18 % increase. Factor analysis of 80 cultivations including variations in bioprocess conditions, growth, and MF detection identified morphology and total MF number, mainly influenced by the cultivation systems, as key factors in metabolic footprint reproducibility. Molecular network analysis revealed that 48 FP and STR share the highest number of molecular clusters. Our findings reveal that only multidimensional optimization unlocks robust metabolome scalability and reproducibility across cultivation systems, paving the way for the discovery of novel NPs.

**Supplementary Information:**

The online version contains supplementary material available at 10.1038/s41598-026-37748-9.

## Introduction

To date, more than 50 % of known drugs are derived from natural products (NPs)^1^. However, NP discovery campaigns face challenges related to reproducibility, as many NPs identified fail to be replicated when switching from screening conditions to cultivation systems with increased reaction volume^2–6^. This issue is particularly pronounced in the early stages of the discovery process, where NP biosynthesis is typically low and cultivation must be scaled in volume for structural characterization and biological activity studies^2,4,7,8^. The change from microbioreactor systems to larger volumes constitutes an unappreciated engineering challenge, as NP biosynthesis is vulnerable to cultivation conditions and process parameters^2,6^. Hence, the reproducibility and the scalability of NPs lacks a comprehensive understanding of the underlying principles that determine requirements for a successful change in cultivation systems in early NP discovery^9–12^.

The scale-up of bacterial fermentation processes presents a multifaceted challenge, as it requires addressing numerous bioprocess and physical parameters, for example agitation, dissolved oxygen, and power input^13,14^. Specifically, the change from shaken to stirred cultivation systems is particularly challenging due to the significant differences in several bioprocess parameters (Table 1). The classical approach for scale-up relies on empirical rules based on the principle of similarity and dimensional analysis, aiming to maintain specific bioprocess parameters and system states constant^12,13^. The most commonly applied criteria rely on the specific power input, the oxygen transfer coefficient, the heat transfer rate or the gas volumetric flow rate^13,14^. Maintaining a constant oxygen transfer coefficient is preferred for scaling from shaken to stirred cultivation systems^13,14^, as reported for the growth and bioprocesses of *Escherichia coli*^15–19^, *Corynebacterium glutamicum*^20,21^, *Azospirillum brasilense*^22^, and *Gluconobacter oxydans*^16^.

**Table 1.** Bioprocess parameter of shaken and stirred cultivation systems. The cultivation systems exhibit notable differences in their bioprocess parameters and available measurements during culturing.

**Cultivation systems**	**Material**	**Agitation mode**	**Flow pattern**	**On-line measurements**^†^	**Working volume** **[mL]**	**Maximum OTR** **[mM/h]**	**Maximum power input** **[W/m**^**3**^**]**	**Reference**
STR	Steel, glass or plastic	Stirred	Defined	pH, dissolved oxygen, temperature, optical density, foam detection, pressure, gas flow, off-gas, substrate and product concentration	> 8	377	1100	^5,88–90^
BSF	Glass or plastic	Shaken	Chaotic	pH, dissolved oxygen, temperature,backscatter, fluorescence, pressure, off-gas, substrate and product concentration	> 5	100	260	^5,48,51,90,91^
48 FP	Polystyrene	Shaken	Chaotic	pH, dissolved oxygen, temperature, backscatter and fluorescence	0.8–1.9	110	-	^5,19,49^

^*†*^*It should be noted that on-line measurements are only available in BSF with additional applications or single-use flasks and generally cannot be combined.*

The cultivation and scale-up of bacteria from the *Streptomyce*s genus present an even more complex challenge^23,24^. Unlike unicellular bacteria in submerged cultures, *Streptomyces* form multinucleate hyphae that can aggregate into pellets^25,26^. This complex growth morphology has a significant impact on both the growth and NP biosynthesis^23,24,26,27^. Consequently, the scale-up of *Streptomyces* species cultivation has been extensively studied to achieve comparable growth and biosynthesis of various proteins, including *Mycobacterium tuberculosis* alanine–proline–rich antigen, *Rhodothermus marinus* cellulase, cholesterol oxidase, L-asparaginase, keratinase, and NPs such as oxytetracycline, actinorhodin, platensimycin and platencin, undecylprodigiosin, natamycin, and pristinamycin^23,24,26,28–38^. Only two studies have considered scale-up criteria^32,34^. Specifically, the scale-up criterion of maintaining specific power input was applied to ensure consistent forces on the complex cell structures, with the goal of achieving comparable biomass and alanine–proline–rich *O*-mannosylation^32,34^. However, maintaining specific power input and the other applied strategies of the aforementioned studies were not straightforward for scale-up, requiring further efforts in medium and process optimization^23,24,28–38^. Medium optimization included the addition of mineral salts^29^, modifications to medium components and their concentrations^30,31,37^, evaluation of complex versus minimal media^35^ and the incorporation of buffer solutions^23,36^. Moreover, the supplementation of small organic molecule elicitors was used to induce secondary metabolite (SM) biosynthesis in *Streptomyces* strains^39,40^. In particular, the addition of ethanol had a stimulatory and inductive effect on the biosynthesis of NPs when added to the medium^41–44^. However, sustaining a constant ethanol concentration appeared to be challenging due to the ethanol partitioning between the liquid and gaseous phases. The quantity of ethanol that is lost over time is an inherent property of the cultivation system, as has been demonstrated by the quantification of the ethanol evaporation in different cultivation systems^45–47^. Lastly, process optimization strategies involved altering fermentation modes^31^, adjusting fermentation parameters^28–32,37,38^, extending fermentation length^36^, and modifying the inoculation process^24,29,38^ to achieve successful scale-up.

Based on these efforts, studies hypothesized that cell morphology, in combination with bioprocess parameters, constitutes a critical factor in the successful scale-up of *Streptomyces* cultivations from shaken to stirred cultivation systems^23,24,28–38^. To understand cellular responses during scale-up processes, a cell system-level analysis has proven beneficial, as demonstrated by transcriptomics and secretomics analyses conducted during *Streptomyces* scale-up^23^. Regarding the metabolome, the effect of cultivation condition from shaken to stirred cultivation systems on the scalability and reproducibility of the metabolic footprint of *Streptomyces* species remains unclear.

For the first time, the scalability and reproducibility of the metabolic footprint of SMs secreted by *Streptomyces griseochromogenes* was evaluated in three cultivation systems: baffled shake flasks (BSF), 48 flower plates (FP) and a bench-top stirred tank bioreactor (STR), which are typically used in the early stages of NP discovery. The mass features (MFs) appearance was used as a proxy for the SM space of *S.* *griseochromogenes*, enabling a comparison of the metabolic footprint and relevant bioprocess parameters across the cultivation systems and conditions. Maintaining either a constant oxygen transfer rate (OTR) or the composition of the medium, i.e. the amount of added ethanol as an elicitor to induce SM biosynthesis, resulted in a poor metabolic footprint that was difficult to compare across cultivation systems. However, allowing variation in these factors improved comparability to a moderate extent. Factor Analysis from 80 cultivations identified key process factors, guiding the selection of conditions for similar metabolic footprints. Lastly, molecular network analysis revealed a greater overlap between the SM families of 48 FP and STR than with BSF, which is mainly used in NP discovery. This work highlights how integrating multiple process parameters, with a strong emphasis on cultivation system choice, is essential for achieving reproducible and scalable metabolome profiles and unlocking new opportunities for identifying novel molecules in NP discovery.

## Results

### Cultivation systems at constant oxygen transfer rate

To evaluate whether the scale-up criterium, which aims to maintain similar oxygen availability, result in similar metabolic footprints of *S.* *griseochromogenes* across the cultivation systems, a constant theoretical OTR of 75 mM/h was tested using 48 FP, BSF, and a bench-top STR. Ethanol was supplemented as a small organic molecule elicitor to stimulate SM biosynthesis in *Streptomyces* strains^41–44^. Due to its evaporation, the ethanol quantity changes over time, thereby generating volatile ethanol levels that cells are exposed to in the cultivation systems. The loss of ethanol is an inherent property of the cultivation system, as has been demonstrated by the quantification of ethanol evaporation in different cultivation systems^45–47^. The growth, cell morphology and MFs formation were studied over a five-day cultivation in glucose-yeast-malt extract (GYM) medium supplemented with 3 % *[v/v]* ethanol at 30 °C. The theoretical OTRs in this study were obtained and calculated based on data from literature and manufacturer specifications^48–51^. Biomass, glucose and ethanol concentrations were measured daily. Cell morphology was determined at the end of the cultivation. To assess the metabolic footprint across the cultivation systems, an untargeted metabolomics workflow was applied to supernatants of the culture broths collected daily. Only MFs matching the specific criteria, MS/MS availability, abundance over 1000 counts across all available biological duplicate and absence in medium controls and the pre-culture, were included in further analyses.

**Fig. 1:**
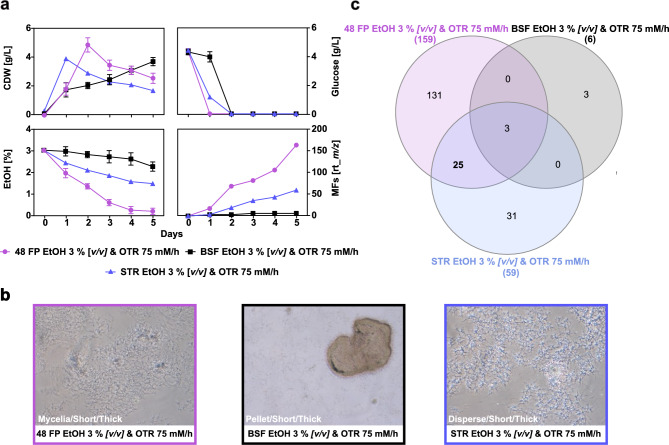
Growth and metabolic footprint at constant oxygen transfer rates in cultivation systems. **a** The graphs display the biomass formation, the glucose consumption, the ethanol depletion and the MF detected as a proxy of the metabolic footprint of *S.* *griseochromogenes* during cultivation in 48 FP (purple circles), BSF (black squares) and STR (blue triangles). *S.* *griseochromogenes* was cultivated in GYM medium with 3 % *[v/v]* ethanol at a theoretical OTR of 75 mM/h for five days at 30 °C. The theoretical OTR was obtained from the specifications provided by the manufacturer and published literature^48–51^. For 48 FP and BSF, samples were taken daily from two independent biological replicates, with results presented as mean values and error bars indicating the standard deviation. For STR, samples were likewise taken daily but derived from a single cultivation per condition due to feasibility constraints. **b** The images, captured at the end of the cultivation period using a 10x magnification lens, depict the main morphology observed in the cultivation systems. **c** The Venn diagram displays the degree of overlap of the detected MFs at the end of the cultivation period across the cultivation systems. For metabolic footprint determination, an untargeted metabolomic workflow was employed utilizing the collected supernatant extracts from each sample to measure the appearance of MFs, where an individual MF is a detected ion that is grouped with its respective retention time (rt_*m/z*). Only those MFs that satisfied the following criteria were considered for the metabolic footprint analysis: an abundance greater than 1000, available MS/MS fragmentation and detection in two replicate cultivations. The MFs that originated from pre-culture and medium control samples were excluded.

Despite the constant OTR, the growth and substrate consumption of *S.* *griseochromogenes* showed considerable differences in the three cultivation systems (Figure 1a). In particular, the biomass formation and the time required to reach the secondary growth phase differed across the three systems. The highest biomass concentration was obtained in the 48 FP after two days of 5 g/L and in the STR after one day with 4.1 g/L. After this peak, biomass concentrations in 48 FP and STR slowly declined and reached final concentrations of 2.8 g/L and 1.9 g/L on day five, respectively. This decline may result from the complex behaviour and morphogenesis of *Streptomyces* species in submerged cultures with high power input and mechanical agitation^52,53^. It can be hypothesized that cell lysis is the primary factor contributing to the observed decrease in biomass concentrations in 48 FP and STR. In contrast to the growth observed in 48 FP and STR, the growth in BSF continued to increase even after full consumption of glucose and reached 3.9 g/L after five days at the end of cultivation. Glucose was fully consumed within one day in 48 FP, and within two days in STR and BSF. After glucose depletion, other complex carbohydrates such as maltose, were still available in the complex GYM medium. Although we did not quantify their consumption, glucose is known to be preferentially utilized and to repress the uptake of alternative carbon sources, which are therefore expected to be metabolized mainly after glucose depletion and to contribute to sustained carbon availability over the five-day cultivation period^54–60^. Ethanol concentrations at the end of cultivation remained 0.6 g/L in 48 FP, 10 g/L in BSF, and 5.5 g/L in STR. We excluded the consumption and use of ethanol for growth, because the slow decrease in ethanol concentration in the cultivation systems over time followed an exponential decay, pointing towards the evaporation of ethanol rather than consumption by the biomass. After five days, the cell morphology of *S.* *griseochromogenes* exhibited notable differences across the cultivation systems (Figure 1b). In 48 FP, both dispersed cells and mycelia formation were observed, while BSF exhibited pellet agglomeration and STR showed completely dispersed growth. Next, the changes in metabolic footprint across the three systems were analyzed and showed notable differences (Figure 1a/c). The highest number of MFs was found after five days of cultivation in 48 FP with 159 MFs, followed by STR with 59 MFs and BSF with six MFs. A notable increase in MFs was seen after glucose was fully consumed, at the onset of the secondary cultivation phase. MFs continued to increase in 48 FP and STR until the end of cultivation. The highest overlap of MFs was between 48 FP and STR, with 25 shared MFs. Three MFs were common across all systems, and 131 unique MFs were identified in 48 FP, three in BSF and 31 MFs in STR. The low metabolic footprint overlap of 18 % suggests that the cultivation system specific characteristics greatly influence the biosynthesis of the SMs space. This finding underscores that maintaining oxygen availability alone is insufficient to ensure system similarity for NP discovery.

### Impact of varying single culture conditions on metabolic footprint

To evaluate the impact of varying cultivation conditions on the metabolic footprint of *S.* *griseochromogenes* across the shaken cultivation systems and STR, the effects of ethanol concentrations ranging from 1 % *[v/v]* to 5 % *[v/v]* and agitation speeds corresponding to theoretical OTRs from 19 mM/h to 119 mM/h were independently assessed, while keeping the other bioprocess parameter constant. The different ethanol concentrations were first tested at a fixed OTR of 75 mM/h. Growth, cell morphology, and metabolic footprint were analyzed at the end of the five-day cultivation period.

**Fig. 2:**
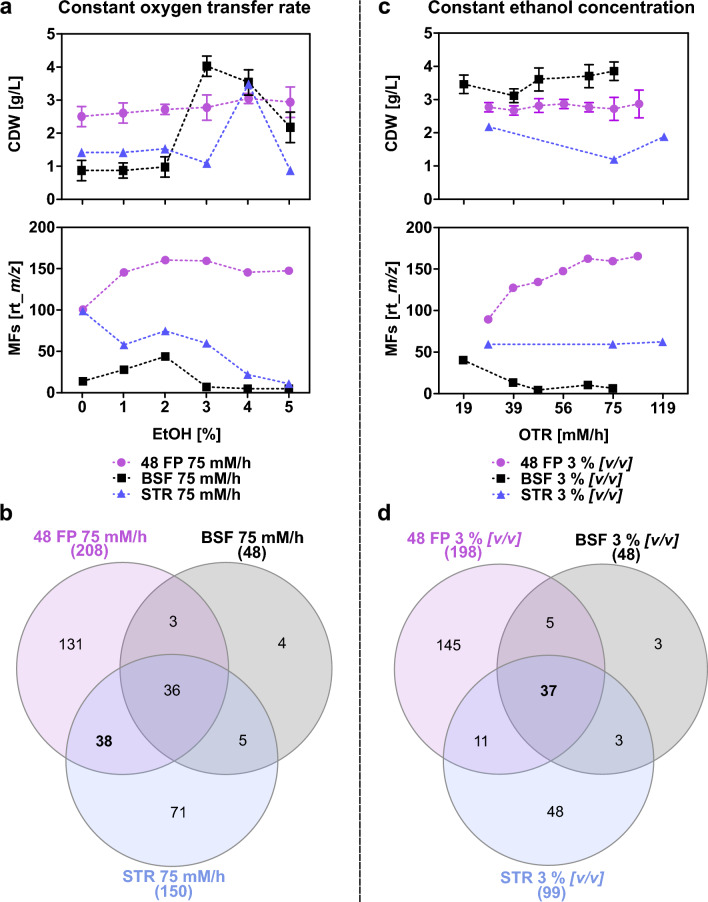
Growth and metabolic footprint at a single varying condition.** a** The graphs illustrate the biomass formation and MFs appearance of *S.* *griseochromogenes* during cultivation in GYM medium and with ethanol concentrations ranging from 1 % *[v/v]* to 5 % *[v/v]*. The cultivation was conducted at a theoretical OTR of 75 mM/h for five days at 30 °C in 48 FP (purple circles), BSF (black squares) and STR (blue triangles). For 48 FP and BSF, samples were collected from two independent biological replicates after five days, with results presented as mean values and error bars representing the standard deviation. For STR, samples were likewise collected after five days but derived from a single cultivation per condition due to feasibility constraints. **b** The Venn diagram illustrates the overlap of all detected MFs in the cultivation systems across the tested ethanol concentrations after five days of cultivation. **c** The graphs display biomass formation and MF formation in *S.* *griseochromogenes* during cultivation in GYM medium with a fixed ethanol concentration of 3 % *[v/v]* and varying agitation speeds, resulting in theoretical OTRs ranging from 19 mM/h to 119 mM/h over five days at 30 °C in 48 FP, BSF, and STR. For 48 FP and BSF, samples were withdrawn from two independent biological replicates after five days, with results presented as mean values and error bars indicating the standard deviation. For STR, samples were also withdrawn after five days but from a single cultivation per condition due to feasibility constraints. **d** The Venn diagram provides an analysis of the overlap of all detected MFs at all tested agitation speeds at the end of the cultivation across all cultivation systems.

Across varying ethanol concentrations, biomass formation in 48 FP remained consistent, peaking at 4 % *[v/v]* with 3 g/L (Figure 2 a). In BSF and STR, increasing ethanol concentrations enhanced growth, with the highest recorded at 3.9 g/L at 3 % *[v/v]* in BSF and 3.4 g/L at 4 % *[v/v]* in STR. Cell morphology in 48 FP varied, with mycelia formation observed at ethanol concentrations from 1 % *[v/v]* to 3 % *[v/v]*, and total dispersed growth at 4 % *[v/v]* and 5 % *[v/v]* (Supplementary Table S1). In BSF, pellets were formed across all ethanol conditions while we observed that more dense pellets were formed with increasing ethanol concentration. These dense pellets allowed for growth in the presence of even higher ethanol concentrations. The cell morphology in STR exhibited mainly dispersed growth with the exception at 2 % *[v/v]* ethanol, where we observed loose cell pellets. The impact of varying ethanol concentrations at constant OTRs on the amount of MFs was evaluated. For varying ethanol concentrations, the highest number of MFs were obtained at 2 % *[v/v]* ethanol in 48 FP with 160 MFs and in BSF with 43 MFs, followed by a slight decline with higher ethanol concentrations (Figure 2a). In STR, ethanol addition reduced the MF formation, decreasing from 98 MFs in the ethanol-free medium to 74 MFs at 2 % *[v/v]* ethanol. Across all ethanol concentrations, a total of 208 MFs were identified in 48 FP, 48 MFs in BSF, and 150 MFs in STR. The highest overlap was observed between 48 FP and STR, with 38 shared MFs, and 36 MFs were common across all systems (Figure 2b). Additionally, 131 MFs were unique to 48 FP, four MFs to BSF, and 71 MFs to STR.

Next, varying oxygen availabilities at a constant 3 % *[v/v]* ethanol concentration were investigated. Across varying theoretical OTRs generated by adapting the agitation speeds, biomass formation was consistent in 48 FP and BSF, with the highest concentrations of 2.9 g/L in 48 FP and 3.9 g/L in BSF (Figure 2c). In STR, increasing oxygen availability decreased biomass, with the highest recorded at 2.2 g/L at a theoretical OTR of 25 mM/h. Cell morphology in 48 FP varied, showing pellet formation at 25 mM/h and mycelial growth at higher OTRs (Supplementary Table S1). BSF exhibited consistent pellet formation, while STR showed dispersed growth across all conditions. At varying OTRs, MF formation in 48 FP increased, reaching a maximum of 165 MFs at a theoretical OTR of 87 mM/h (Figure 2c). In STR, MF formation remained constant, peaking at 62 MFs at 119 mM/h. In BSF, MF formation decreased with increasing agitation, reaching a maximum of 40 MFs at 19 mM/h. Across all agitation speeds, a total of 198 MFs were identified in 48 FP, 48 MFs in BSF, and 99 MFs in STR. The highest overlap was observed across all cultivation scales, with 37 shared MFs (Figure 2d). Additionally, 145 unique MFs were observed in 48 FP, three MFs in BSF, and 48 MFs in STR.

The moderate 18 % increase in metabolic footprint overlap during varying single culture conditions such as the ethanol concentration in the medium and the oxygen availability suggests that SMs biosynthesis in cultivation systems is influenced by a complex interplay of bioprocess parameters. Simple adjustments to individual factors are insufficient to reliably reproduce the metabolic footprint across different cultivation systems.

### Relevance of bioprocess factors to metabolic footprint

To further explore the relation of the bioprocess parameters and the metabolic footprint of *S. griseochromogenes*, a Factor Analysis of Mixed Data was performed (Supplementary Table S2), identifying the smallest number common factors. These common factors are seen as qualitative variables of unobserved characteristics of the cultivation systems. The dataset (Supplementary Table S3) included varied bioprocess parameters, growth characteristics, and metabolic footprints from 80 cultivation conditions across the three cultivation systems. The bioprocess parameters encompassed ethanol concentrations and theoretical OTRs. Growth was evaluated based on biomass concentration and cell morphology, including filament length categorized as short or long, filament thickness classified as thick or thin, and overall cell form grouped into dispersed growth, mycelial formation, and pellet agglomeration (Supplementary Table S4). The metabolic footprint comprised the total number of MFs detected under each cultivation condition (overall MFs), unique MFs identified in only one condition (unique MFs), and those MFs that were detectable in at least one condition in STR (scaled-up MFs).

**Fig. 3:**
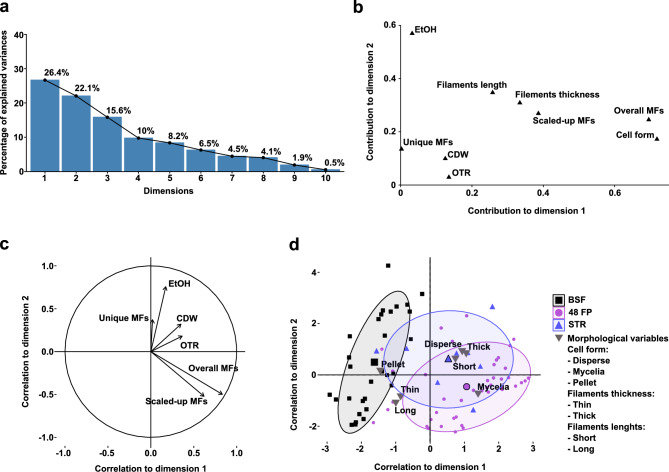
Identification of key factors impacting the metabolic footprint using Factor Analysis of Mixed Data. **a** The graph represents a scree plot, illustrating the number of dimensions and their corresponding eigenvalues. **b** The graph illustrates a variables plot which shows the correlation between all quantitative and qualitative factors and their contribution to the dimensions, determined in factor analysis. Quantitative factors included the theoretical OTR, CDW, ethanol concentrations (EtOH), the total number of MFs (Overall MFS), the unique MFs and the MFs that were detectable in STR (Scaled-up MFs). Quantitative factors encompassed the filament lengths and thickness, as well as the general cell form. **c** The graph represents a monoplot of quantitative factors, which illustrate the relationship between factors and the quality of the representation of the factors by the two principal dimensions. **d** The graph presents a factor map illustrating the interrelationship between experiments in 48 FP (purple circles), BSF (black squares), and STR (blue triangles) and their correlation to the dimensions and qualitative factors (gray inverted triangles), including cell form (pellet, mycelia, and disperse), filament length (long and short), and filament thickness (thin and thick). Colored ellipses indicate a 68 % confidence level for each cultivation system.

The factor analysis identified ten principal dimensions required to explain the variance in the dataset, as shown in the scree plot (Figure 3a). The first three dimensions accounted for 64.1 % of the total variance, with 26.4 %, 22.1 %, and 15.6 % explained by the first, second, and third dimension, respectively. Overall MFs and cell form contributed most to the first dimension, ethanol concentration had the highest loading on the second dimension, and cell form, theoretical OTR, biomass concentration and unique MFs were most influential for the third (Supplementary Figure S1). Based on these contributions, we deduced that the first dimension reflects differences due to cultivation systems, the second one the changes due to medium variations, and the third one the adjustments in agitation speed. Since the third dimension had a minimal impact on scaled-up MFs, the analysis focused on the first two dimensions as the most important for describing differences in the metabolic footprint across the three cultivation systems. To further investigate factor contributions to the dimensions and their interrelations, a variables plot was generated (Figure 3b). Unique MFs showed no contribution to the first dimension and only minimally to the second dimension, indicating a poor representation by the second dimensions and a negligible influence by cultivation systems. Scaled-up MFs and filament form clustered with overall MFs and cell form, all exhibiting similar loadings on the second dimension, reflecting their close interdependence. While the factors overall MFs and cell form showed higher loadings on the first dimension, scaled-up MFs and filament form displayed comparable loadings across both dimensions. This indicates that morphology, i.e. the type of filaments and cell form, primarily influences the diversity and scalability of the metabolic footprint. Whereas cultivation systems predominantly affecting cell form, the type of filaments is equally influenced by ethanol concentrations and cultivation systems. To gain deeper insight into the correlation between the quantitative factors, a monoplot was generated (Figure 3c). The vectors for unique MFs and ethanol concentration are closely aligned, suggesting a strong correlation. Overall MFs and scaled-up MFs form a small angle, indicating a high positive correlation. In contrast, the wide angle relative to the unique MFs indicates a weak negative correlation with the overall MFs and scaled-up MFs. Lastly, a factor map (Figure 3d) was generated to evaluate relationships between cultivation systems, individual experiments, and qualitative factors. The map highlights experimental similarities and detected morphologies, as indicated by overlapping confidence ellipses and the proximity of qualitative factors and individual experiments. The results from all 80 cultivations across the three cultivation systems were mapped onto the main two dimensions. BSF exhibited exclusively negative loadings on the first dimension and transitioned from negative to positive loadings on the second dimension. In contrast, 48 FP and STR predominantly showed positive loadings on the first dimension, with loadings ranging from negative to positive on the second dimension. Additionally, the overlap of confidence intervals is greater between 48 FP and STR than with BSF, suggesting that the metabolic footprints in 48 FP and STR are more similar. The proximity of morphological variables confirms our microscopic observations that BSF promotes pellet formation with thin, long filaments, 48 FP supports mycelial growth with short, thick filaments, and STR favors dispersed growth with short, thick filaments. This indicates that cultivation in 48 FP and STR results in more comparable experimental results and morphology compared to BSF.

**Fig. 4:**
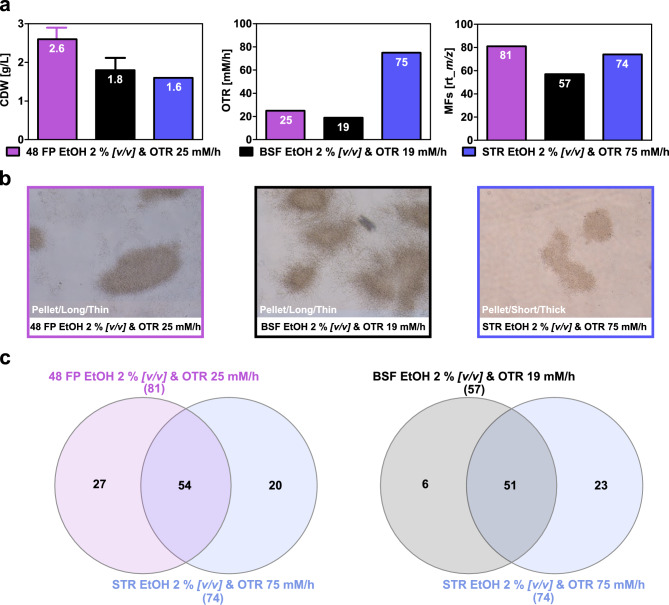
Growth and metabolic footprint of cultivation conditions with similar profiles in the factor map.** a** The bar charts depict the biomass, theoretical OTR, and MF formation for the cultivation conditions exhibiting similar profiles in the factor map and the highest degree of metabolic footprint overlap from *S.* *griseochromogenes* after five days of cultivation in 48 FP (purple), BSF (black), and STR (blue). These conditions included cultivation in GYM medium with 2 % *[v/v]* ethanol at a theoretical OTR of 25 mM/h in 48 FP, 19 mM/h in BSF, and 75 mM/h in STR. For 48 FP and BSF, the bars represent mean values with error bars indicating the standard deviation from two independent biological replicates. For STR, bars correspond to a single cultivation. **b** The presented microscopic images, captured at the end of the cultivation using a 10x magnification lens, illustrate the morphology of the bacteria across the cultivation systems. **c** The Venn diagrams displays the degree of overlap between the detected MFs at the end of the cultivation, obtained from 48 FP to STR and from BSF to STR.

By selecting experiments in close proximity on the factor map, cultivation conditions with the highest overlap in metabolic footprints across all three cultivation systems were identified (Figure 4). In these conditions, biomass formation was comparable at 2.6 g/L in 48 FP, 1.8 g/L in BSF, and 1.6 g/L in STR (Figure 4a). Agitation speeds varied notably, resulting in theoretical OTR values of 25 mM/h in 48 FP, 19 mM/h in BSF, and 75 mM/h in STR. Overall MF formation was comparable across all systems, with 81 MFs in 48 FP, 57 MFs in BSF, and 74 MFs in STR. Cell morphology was consistent, with loose pellets and short, thick filaments in STR and long, thin filaments in 48 FP and BSF (Figure 4b). The highest MF overlap occurred between 48 FP and STR with 54 MFs, followed by BSF and STR with 51 MFs. Additionally, 27 unique MFs were observed in 48 FP, six MFs in BSF, and a maximum of 23 unique MFs in STR (Figure 4c). This analysis highlights that the cultivation systems itself play a crucial role in determining the reproducibility of the metabolic footprint.

### Molecular characterization of metabolic footprint

**Fig. 5:**
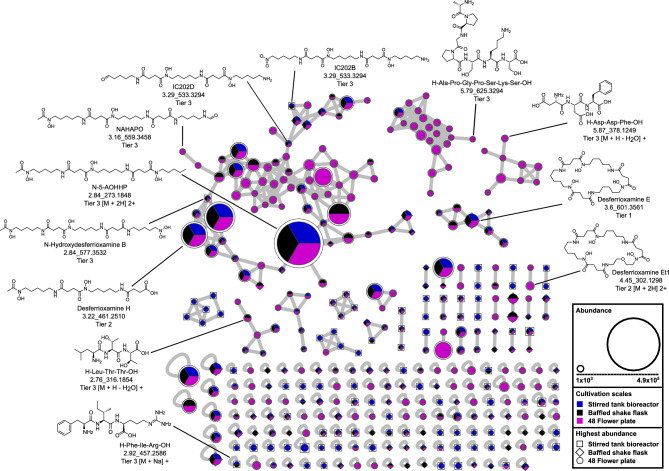
Molecular network of all detected mass features across the cultivation scales. The graph illustrates a molecular network, with the appearance of each node during cultivation of *S.* *griseochromogenes* in 48 FP (purple), BSF (black) and STR (blue), presented in a pie chart. The size of the pie chart displays the overall highest observed intensity in this study. The shape surrounding the pie chart represents the cultivation systems, with 48 FP (circle), BSF (diamond) and STR (square), in which the highest abundance was observed. The nodes represent MF, and selected nodes are labeled with their chemical structure, name, feature (rt_*m/z*) and the annotation confidence level (Tier 1–4). All selected nodes represent [M + H]+ unless otherwise specified.

To analyze the molecular relationships of SMs and their occurrence in the cultivation systems, a molecular networking analysis was conducted using all detected MFs from all cultivation conditions (Figure 5). The analysis generated single and clustered nodes based on LC-MS/MS fragmentation pattern similarity. Annotations were generated through retention time and spectral comparisons with chemical standards, the GNPS online spectral library, or the *in-silico* tool SIRIUS. A detailed list of detected MFs and annotations is provided in the supplementary in table S5.

Molecular networking revealed 145 single nodes, 23 double nodes, and 12 molecular families comprising three or more nodes (Figure 5). Among the single nodes, 38 were unique to 48 FP, 12 to BSF, and 33 to STR. Double nodes included four unique to 48 FP and five to STR. Molecular families included one with nine nodes exclusive to 48 FP and one with six nodes unique to STR. Six single nodes and two double nodes were common between the shaken systems, with at least one node in each cluster shared. Scalable nodes from a shaken system to STR included 14 single nodes, two double nodes, and one molecular family including five nodes, with at least one node in shaken systems and one node in STR in each cluster. Nodes detected across all systems included 42 single nodes, 10 double nodes, and nine molecular families, each containing at least one node detected in all cultivation systems.

In conclusion, large clusters often contain at least one node, i.e. one representative of a molecular family that is common to all cultivation systems. However, molecular families, and small clusters in particular, are produced specifically in one cultivation system, which emphasizes the challenge of obtaining a scalable and robust metabolic footprint across different systems.

**Fig. 6:**
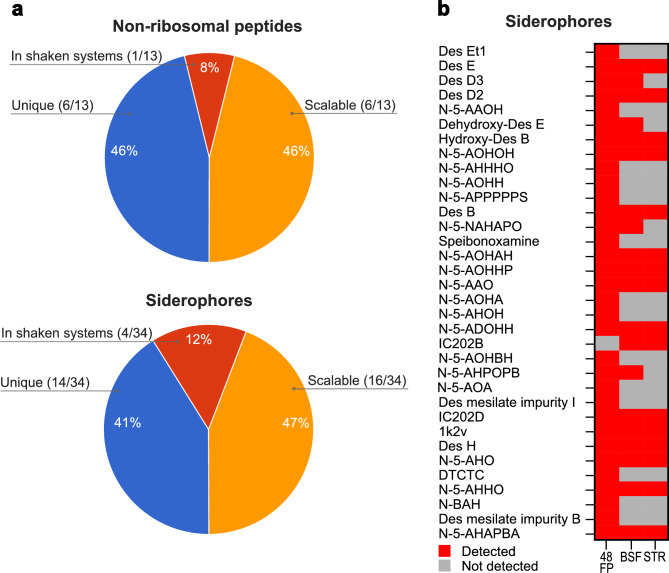
Appearance and structural modification of annotated mass features in the cultivation systems. **a** The pie charts demonstrate the occurrence of the annotated non-ribosomal peptides and siderophores across the cultivation systems. The pie charts are divided into three categories: (1) annotated MFs that were exclusively identified in a single cultivation system (blue), (2) annotated MFs that were identified in both the 48 FP and BSF shaken cultivation systems (red) and (3) annotated MFs that were identified in all cultivation systems or in one of the shaken cultivation systems and in the STR (orange). **b** The qualitative heat map illustrates the detection (red) or absence (grey) of annotated siderophores in the cultivation systems. The full names and further information of the annotated siderophores are listed in the supplementary in Table S5 and Table S6.

Next, we compared the chemical structure of the identified nodes. A purine nucleoside NP, futalosine, was identified with a single node. Three clusters, two double nodes, and five single nodes were annotated as non-ribosomal peptides (NRPs). The largest cluster of 68 nodes, alongside two smaller clusters, three double nodes, and four single nodes, was linked to siderophores, all desferrioxamine compounds. The desferrioxamine derivative N-5-AOHHP, lacking one amino group compared to Des B, showed the highest overall abundance of 4.9 × 10^5^ counts in 48 FP and was detected across all systems. The structural modifications and distribution of annotated NRPs and siderophores across cultivation systems were further analyzed. A total of 13 NRPs were annotated (Figure 6 & Supplementary Table S6). Five, including L-asparagyl-L-asparagyl-L-prolyl-L-serine, were unique to 48 FP, while leupeptin Pr was exclusive to BSF. L-leucyl-L-threonyl-L-threonine was shared between the shaken systems, and four NRPs, such as L‑lysyl-L-threonyl-L-isoleucine, were found across all systems. Two NRPs, including acetylleucylleucyllysinal hydrate, were common to BSF and STR. Structural modifications included chain length variations, chemical group substitutions, and amino acid changes. For example, acetylleucylleucyllysinal hydrate features a hydroxy group, a lysinal instead of argininal, and an acetyl group replacing a propionyl group compared to leupeptin Pr. Additionally, L-alanyl-L-prolyl-glycyl-L-prolyl-L-seryl-L-lysyl-L-serine, a heptapeptide, contains four more amino acids than leupeptin Pr, a tripeptide. A total of 34 siderophores, all belonging to the desferrioxamine class, were identified (Figure 6 & Supplementary Table S7). Of these, 14, including Des Et1, were exclusive to 48 FP, while four, such as dehydroxy-Des E, were shared between the shaken systems. In total, 15 desferrioxamine derivatives, including Des D2, were present across all systems, and IC202B detected in BSF and STR. Structural modifications included cyclization, chain-length variations, and functional group modifications. Examples include Des Et1 with an additional ether structure, dehydroxy-Des E lacking a hydroxy group, Des D2 missing one methyl group compared to the cyclic Des E, and IC202B containing a nitro group instead of hydroxyacetamide compared to Des B.

The molecular network analysis revealed that the biosynthesis of specific SMs and their derivatives is strongly influenced by the choice of the cultivation system. The highest diversity of SMs and derivatives was observed in the 48 FP. This may be explained by a higher degree of stress or by a specific combination of stressors only present in the 48 FP. For instance, unlike BSF and STR, the 48 FP are made of polystyrene, which has been associated with increased aggregation of intracellular reactive oxygen species^61,62^. This elevated stress can promote SM diversification or adjustments of the biosynthetic machinery to meet cellular demands during growth^63,64^. Examples include the occurrence of hydroxy-/dehydroxy-derivatives such as N-5-AHPOPB and N-5-AHOH, and N-tailored analogues such as N-5-AHO, N-5-AOHH, and N-5-NAHAPO, which were exclusively detected in 48 FP. In contrast, the STR showed the lowest number of annotated desferrioxamine derivatives (Figure 6). This could be attributed to the more homogeneous distribution of oxygen and nutrients, which reduces cellular stress and thus limits the formation of modified congeners^65,66^. Thus, increasing stress from cultivation systems correlates with enhanced structural diversification, while optimized cultivation systems favor the conservation of core SM scaffolds.

## Discussion

This study demonstrates that classical scale-up criteria, such as maintaining constant oxygen availability, alteration of bioprocess parameter or culture condition are not straightforward for scaling the metabolic footprint of filamentous bacteria in early NP discovery. The presented research identifies the cultivation system itself as the primary factor influencing the reproducibility of metabolic footprints. Due to the significant variations in bioprocess parameters across the cultivation systems, a multivariate adjustment of these parameters is essential to achieve robust metabolic footprint reproducibility. Furthermore, the analysis conducted highlights the need to consider in particular the cell morphology to enhance the scalability of metabolic footprints.

By maintaining comparable cell morphology, we increased the similarity of the metabolic footprint by 50 % from 48 FP and BSF to the STR. Any variations in bioprocess parameters within the cultivation systems, including fermentation volume, mass transfer, material, and illumination, can affect the morphology of bacteria. The differences in cell morphologies observed in the cultivation systems in our research can be attributed to variations in hydromechanical stress. For instance, at the same volumetric power consumption, the maximum local energy dissipation rate in BSF was found to be 10 times lower than in STR, while microtiter plate cultivation systems demonstrated specific power inputs comparable to STR^24,67,68^. Increased hydromechanical stress causes mycelium disruption, resulting in a more fragmented dispersed growth morphology. This dispersed growth, in turn, impacts the rheological properties of the cultivation liquid, further affecting the mixing performance^24^. In addition to mechanical stress, factors such as nutrient depletion and oxidative stress showed influence on the morphology of *Streptomyces* bacteria^26,69^. For example, the addition of polystyrene, the material of the 48 FP, during cultivation has been associated with the aggregation of intracellular reactive oxygen species^61,62^. Consequently, the cultivation system can significantly influence the cell morphology of *Streptomyces* bacteria, even when similar bioprocess parameters are maintained. The variations in the number of produced MFs can be attributed to the different stress in the cultivation systems, as described above. Even without the addition of external stressors, differences in SMs biosynthesis were observed across microtiter plate cultivation systems, shake flasks, and STR^6^. The stress factors arising from the cultivation systems, such as variations in power input and reactive oxygen species, not only influence morphology but also impact the entry into the secondary cultivation phase and the activation of SM biosynthesis^6^. Consequently, the cultivation systems themself have a massive impact on the cell morphology, growth, SM biosynthesis, and the reproducibility of the metabolic footprint of bacteria^6^.

To improve the reproducibility and scalability of the metabolic footprint in future NP discovery campaigns, three key considerations should be taken into account: Firstly, the selection of an appropriate screening platform is crucial. The screening cultivation system should allow for parallelized cultivation, promote increased SM biosynthesis, and closely mimic the bioprocess parameters of STR^2,6,23,24^. In this regard, the 48 FP presents a promising option as a screening platform, as our studies have shown it achieves the highest SM biosynthesis and comparability to STR in contrast to other cultivation systems^6^. Additionally, its ability to parallelize and monitor growth, pH, and dissolved oxygen will aid in identifying specific conditions to activate novel SMs and increase scalability during early NP discovery. Secondly, the selection of reproducible, scalable, and suitable culture conditions for STR is important. For example, cultivation conditions involving co-cultivation and biotic additives have been reported to present challenges in reproducibility^40,70^. Furthermore, a scale-up of cultivation conditions can be difficult and economically unfeasible, for example with rare earth elements or costly inducers like antibiotics^71,72^. Additionally, cultivation conditions must be compatible with STR requirements, such as preventing damage to probes or seals. Therefore, culture conditions should be designed not only to induce novel SMs but also to ensure scalability. For instance, factors like temperature, pH, medium limitations, and the addition of salts have been shown to enhance the amount of MFs while ensuring reproducibility, scalability, and STR compatibility^39^. Thirdly, considering other bioprocess parameters such as monitoring cell morphology is crucial. Implementing real-time imaging methods could significantly enhance the efficiency of morphology monitoring. For example, in situ microscopy has been employed to monitor bacterial cell morphology during cultivation^73–75^. To further refine this process, deep neural networks can be employed to analyze the resulting images^76^. One such example is the Omnipose algorithm, which utilizes deep learning to automatically identify complex cell morphologies, including filamentous structures like *Streptomyces* bacteria^77^. A successful application of in situ microscopy combined with real-time analysis using convolutional neural networks has been demonstrated for monitoring cell growth in mammalian cells^78^. Another promising approach is Focused Beam Reflectance Measurement, which analyzes laser light backscatter from particles to asses cell morphology and has been used to monitor *Streptomyces* cultures^79,80^. Although these techniques have been successfully implemented in STR, their application in screening platforms, such as microtiter plate cultivation systems, remains an untapped area, with no successful implementations reported to date. With these considerations and future advancements in establishing in situ morphological monitoring techniques in microtiter plate cultivation systems, the potential for successfully elucidating novel SMs during NP discovery will be significantly enhanced.

## Materials and methods

### Reagents

A comprehensive list of the chemicals used for the preparation of the culture medium and for the annotation of the formed SMs, is provided in the supplementary in table S8.

### Strain and media composition

The bacterial strain *S. griseochromogenes* DSM 40499 was obtained from the German Collection of Microorganisms and Cell Cultures (DSMZ, Braunschweig, Germany). The growth medium used was a GYM medium, containing 4 g/L glucose, 4 g/L yeast extract, and 10 g/L malt extract, with the pH adjusted to 7.2. A working cell bank was prepared by inoculating 20 ml of GYM medium in a 100 ml BSF with the cells provided by DSMZ. The culture was then incubated for 24 hours at 30 °C on an orbital shaker set to 200 rpm (50 mm orbit diameter). After the incubation, the bacterial broth was aliquoted and mixed with glycerol to a final concentration of 10 % *[v/v]* to reach a total volume of 1 mL in each tube of the working cell bank. This working cell bank was stored at −20 °C until further use.

### Pre-culture

Inoculum cultures were prepared by adding 1 mL of the working cell bank to 19 mL of GYM medium in a 100 mL BSF and incubated for 24 hours at 30 °C on an orbital shaker set to 200 rpm (50 mm orbit diameter).

### Cultivation in 48 flower plates with the BioLector system

Seven shaking speeds, ranging from 800 rpm to 1400 rpm with 100 rpm steps, were evaluated. The shaking speeds were tested in combination with GYM medium, both without ethanol as control and with five ethanol concentrations ranging from 1 % *[v/v]* to 5 % *[v/v]*. This resulted in a total of 47 cultivations, all carried out in biological duplicate. A separate pre-culture was prepared for each shaking speed, while a single pre-culture was used for each set of ethanol concentrations. For each shaking speed, two rows of a 48 FP with two wells per ethanol concentration were filled with a fixed volume of 0.1 mL of inoculum. The volume of GYM medium added to each well was dependent on the ethanol concentration, with volumes ranging from 0.9 mL of GYM medium for the control to 0.85 mL of GYM medium for 5 % *[v/v]* ethanol. Furthermore, two rows of a 48 FP were completely filled only with the condition 3 % *[v/v]* ethanol at the shaking speed of 1200 rpm. The experimental layout is displayed in the supplementary in Table S9. The 48 FP was closed using a gas-permeable non-woven sealing foil and was cultivated in a BioLector II (Beckman Coulter, Germany) for a period of five days at 30 °C. The selected 48 FP (MTP-48-BOH 3) was equipped with optical sensor spots for the supplementary detection of pH in a range of 3.6 to 6.5 pH and dissolved oxygen from 0 to 100 % oxygen during the cultivation. For the cultivation condition 3 % *[v/v]* ethanol at 1200 rpm, biological duplicates of 1 mL samples were taken once per day for five days. For every other cultivation condition, two wells were harvested and treated as duplicate cultivations of 1 mL samples. Microscopic images were taken from all samples directly after sampling by using the Axio Lab A1 (Zeiss, Oberkochen, Germany). These morphological investigations were done for every sample from the 48 FP, BSF and STR.

### Cultivation in baffled shake flask

Five different shaking speeds, ranging from 100 rpm to 300 rpm in 50 rpm intervals, were tested in BSF, with culture conditions consisting of GYM medium as the control and supplemented with five ethanol concentrations ranging from 1 % *[v/v]* to 5 % *[v/v].* This resulted in a total of 30 cultivation conditions, all carried out in biological duplicate. For cultivation, 500 mL BSF were prepared with a fixed volume of 10 mL of inoculum and an ethanol concentration dependent volume of fresh GYM medium ranging from 90 mL of GYM medium for the control and 85 mL GYM medium for 5 % *[v/v]* ethanol. All flasks were cultivated on orbital shakers (50 mm diameter) for five days at 30 °C. For the cultivation, 15 pre-cultures were prepared as described in the previous section and before inoculation pooled. A total of 1 mL of samples were taken from each flask at the end of the cultivation. In addition, 1 mL of sample was taken from the cultivation condition 3 % *[v/v]* ethanol at 300 rpm once per day. Morphological investigations of BSF samples were carried out as described in the previous section.

### Cultivation in stirred tank bioreactor

The stirrer speed 500 rpm, 1000 rpm and 1500 rpm were tested in DASbox STR (Eppendorf, Hamburg, Germany). For the culture conditions at 500 rpm and 1500 rpm, an ethanol concentration of 3 % *[v/v]* was tested. For the 1000 rpm culture condition, a control without ethanol was included, alongside five ethanol concentrations ranging from 1 % *[v/v]* to 5 % *[v/v]*. This resulted in a total of eight cultivation conditions. Additionally, the reproducibility of both metabolic footprints and morphology in the STR was evaluated by an independent cultivation conducted at 1000 rpm with 1 % *[v/v]* ethanol supplementation, which resulted in a 92 % overlap of total MFs and identical morphology across both experiments (Table S10 & Figure S2). The STR cultivations were started using a fixed volume of 15 mL inoculum and an ethanol concentration dependent volume of GYM medium ranging from 135 mL of GYM medium for the control to 127.5 mL of GYM medium for 5 % *[v/v]* ethanol. All reactors were cultivated with disk flat blade impellers (30 mm diameter) for five days at 30 °C with an air gassing rate set to 3 L/h. For each fermentation, separate pre-cultures were prepared for inoculation. Dissolved oxygen was measured with the VisiFerm RS485 (Hamilton, Bonaduz, Switzerland) and pH was measured with the pH Sensor InPro3253 (Mettler-Toledo, Greifensee, Switzerland) online in all reactors. The pH was not controlled throughout the cultivation. In total, 1 mL samples were withdrawn at the end of the cultivation. For the cultivation conditions 3 % *[v/v]* ethanol at 1000 rpm, 1 mL samples were taken once per day. Morphological investigations of STR samples were done as described above.

### Sample preparation

Broth samples taken from 48 FP, BSF, and STR were completely transferred into pre-dried and pre-weighted microcentrifuge tubes, followed by cell separation via centrifugation for 20 minutes at 3795 ×g and 4 °C using a Sorvall™ RC 5B Plus centrifuge (Thermo Fisher, Waltham, MA, USA). The supernatant was then filtered through a 0.45 μm polyamide filter (Macherey-Nagel, Düren, Germany) and diluted prior to Liquid chromatography-mass spectrometry (LC-MS/MS) analysis 1:2 with ultrapure water.

### Determination of growth and substrate consumption

The biomass, ethanol, and glucose concentrations were determined for each sample. The biomass concentration was calculated by measuring the cell dry weight (CDW), as previously described^6^. The concentration of ethanol and glucose was determined by high-performance liquid chromatography (HPLC) with a refractive index detector (Agilent 1200 Series/1260 Infinity, Agilent Technologies, Santa Clara, USA), as previously described^81^. The concentrations were determined from an external calibration curve of standards ranging from 2 g/L to10 g/L for glucose and from 8 g/L to 40 g/L for ethanol.

### Characterization of the metabolic footprint

To determine the metabolic footprint in the different cultivations, LC-MS/MS measurements were conducted using an ultra-HPLC (1290 Infinity II, Agilent, Santa clara, CA, USA) system coupled with an electrospray ionization-quadrupole-time of flight (Compact, Bruker, Billerica, MA, USA) with the settings previously described^40^. LC-MS/MS data were analyzed using Data Analysis 4.4 (Bruker, Billerica, MA, USA) and MzMine 2.35^82^. The detailed steps, settings and MFs filtering were applied in accordance with the previously described procedure^40^. Furthermore, only those MFs that were present in both biological duplicate samples were further considered. The MFs detected in the pre-culture and the medium control were excluded from the feature lists.

A Factor Analysis of Mixed Data was performed in RStudio version 4.4.1 using the package FactoMineR version 2.11^83,84^, utilizing the information obtained from the used cultivation system, the ethanol concentration, the theoretical OTR, the morphological investigation of cells and filaments, the formation of biomass, and the MFs found and grouped into total number of MFs, unique MFs and MFs that were found in the STR. The data used for factor analysis is given in the supplementary in Table S3 together with the commented R code in the supplementary in Table S2.

Molecular Networking was performed in default mode according to the online GNPS workflow^85^, as described previously^40^. The molecular network was visualized using Cytoscape version 3.6.1^86^. The nodes were labeled as previously described^40^. The visual representation of the distribution of each ion in 48 FP, BSF and STR was achieved using pie charts. Moreover, the dimensions of the pie charts correspond to the overall highest observed abundance of each node. The shape surrounding the pie charts represents the cultivation system in which the highest abundance was observed. The annotation of MFs was conducted using chemical standards, the GNPS online spectral library^85^, or in silico with SIRIUS 5.62^87^, as previously described^40^. In the supplementary the Figure S3 and Figure S4 show examples for annotations. Chemical structures were generated using the ChemDraw 20 software (PerkinElmer, Waltham, MA, USA).

## Supplementary Information

Supplementary Information.

## Data Availability

Upon request to the corresponding author, research data will be made available. Additional supporting information can be found online in the supporting information section at the end of this article.
