# Supplementary material for: Multidimensional strategy enables scalable metabolome diversity in microbial fermentations
Source: Sci Rep. 2026 Jan 29;16:4084. doi: 10.1038/s41598-026-37748-9 (PMC12855292; doi:10.1038/s41598-026-37748-9)
Supplement: Supplementary file 1 — Supplementary Information. [file 41598_2026_37748_MOESM1_ESM.docx]

**Supporting Information**

**Multidimensional strategy enables scalable metabolome diversity in microbial fermentations**

Anton Lindig ^1^, Makram Fataeri ^1,2^, Georg Hubmann ^1^ and Stephan Lütz ^1*^

^1^ Chair for Bioprocess Engineering, Department of Biochemical and Chemical Engineering, TU Dortmund University, Emil-Figge-Straße 66, 44227 Dortmund, Germany.

^2^ Biotechnology, School of Science, Constructor University Bremen, Campus Ring 1, 28759 Bremen, Germany.

Tel: +49 231 755-7488 Email: [anton.lindig@tu-dortmund.de](mailto:anton.lindig@tu-dortmund.de), [mfataeri@constructor.university](mailto:mfataeri@constructor.university), [georg.hubmann@tu-dortmund.de](mailto:georg.hubmann@tu-dortmund.de) and [stephan.luetz@tu-dortmund.de](mailto:stephan.luetz@tu-dortmund.de)

^*^ Correspondence: Stephan Lütz, Chair for Bioprocess Engineering, Department of Biochemical and Chemical Engineering, TU Dortmund University, Emil-Figge-Straße 66, 44227 Dortmund, Germany. Tel: +49 231 755-7488 Email: [stephan.luetz@tu-dortmund.de](mailto:stephan.luetz@tu-dortmund.de)

**Table of contents**

**Table S1**…………………………………………………………………………….**Page 3 - 6**

**Table S2**……………………………………………………………………………**Page 7 - 9**

**Table S3**……………………………………………………………………………**Page 9 - 14**

**Table S4**……………………………………………………………………………….**Page 15**

**Table S5**…………………………………………………………………………...**Page 16 - 31**

**Table S6**……………………………………………………………………………**Page 32 - 34**

**Table S7**…………………………………………………………………………… **Page 35 - 37**

**Table S8**…………………………………………………………………...…………… **Page 38**

**Table S9**……………………………………………………………...…………… **Page 39 – 40**

**Table S10**…………………………………………………………...…………… **Page 41 – 43**

**Figure S1**……………………………………………………………………………….**Page 44**

**Figure S2**……………………………………………………………………………….**Page 45**

**Figure S3**……………………………………………………………………………….**Page 46**

**Figure S4**……………………………………………………………………………….**Page 47**

**Table S1:** Microscopic images of bacteria under varying agitation speeds and ethanol concentrations across cultivation scales. The images were taken at the end of the five-day cultivation period using a 10x magnification lens.

| **EtOH [% *v/v*]** | | | **control** | **1** | **2** | **3** | **4** | **5** |
| --- | --- | --- | --- | --- | --- | --- | --- | --- |
| **Cultivation scale^†^** | **Agitation speed [rpm]** | **Theoretical OTR [mM/h]** |  |  |  |  |  |  |
| 48 FP | 800 | 25 | 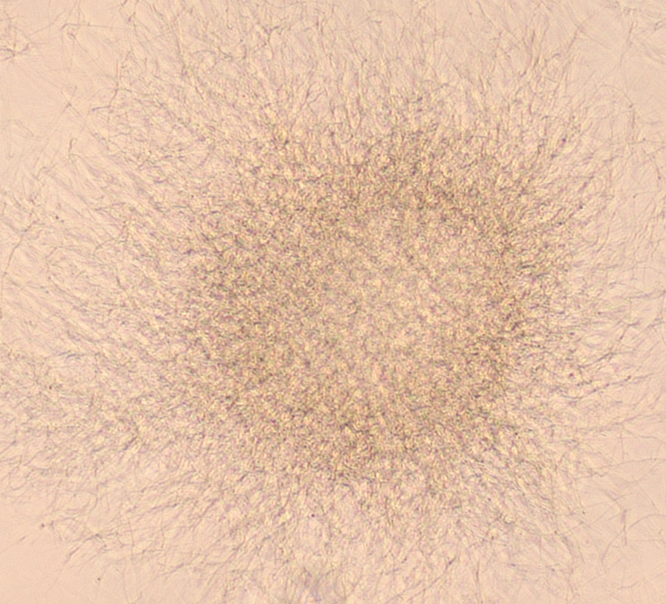  **Pellet/Long/Thin** | 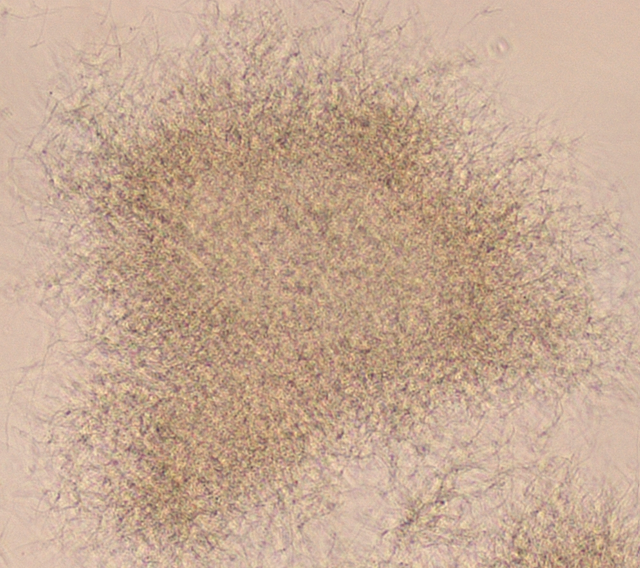  **Mycelia/Long/Thin**  **Pellet/Short/Thin**  **Pellet/Long/Thin**  **Pellet/Long/Thin**  **Pellet/Short/Thick** | 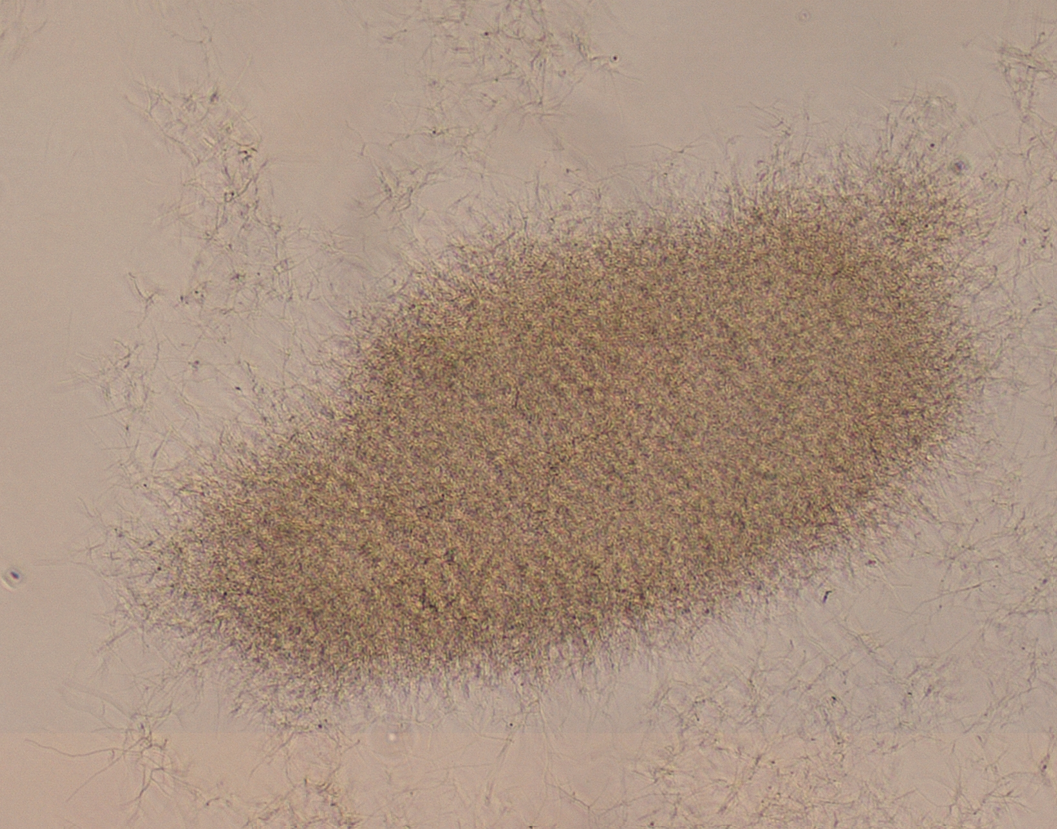 | 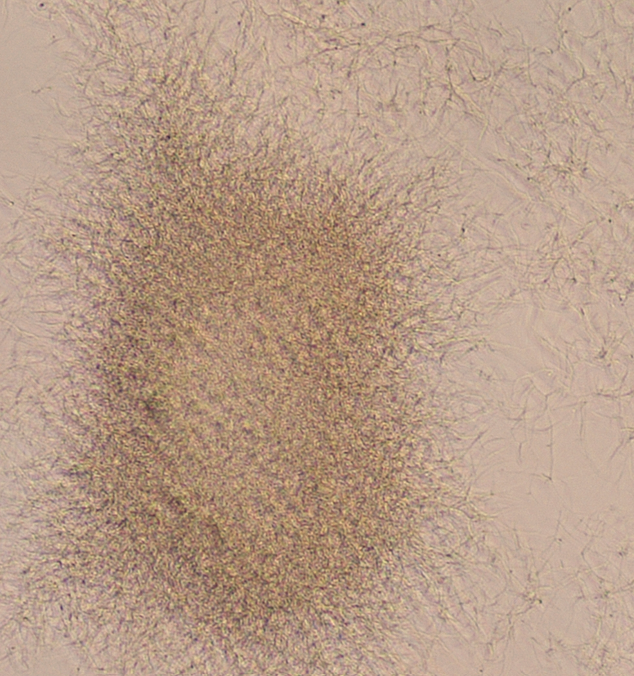 | 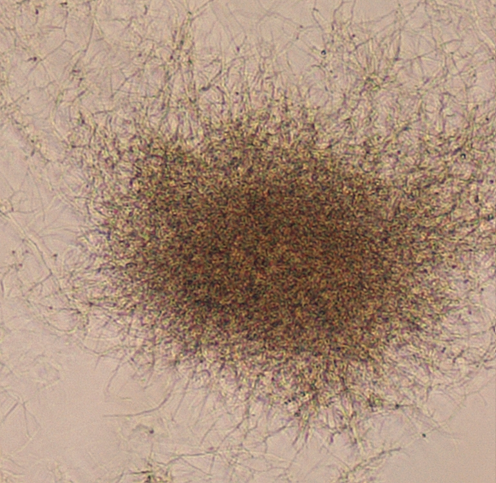 | 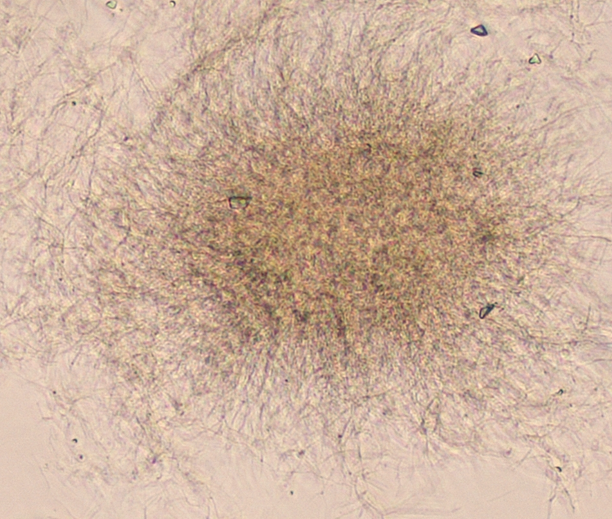 |
| 48 FP | 900 | 39 | 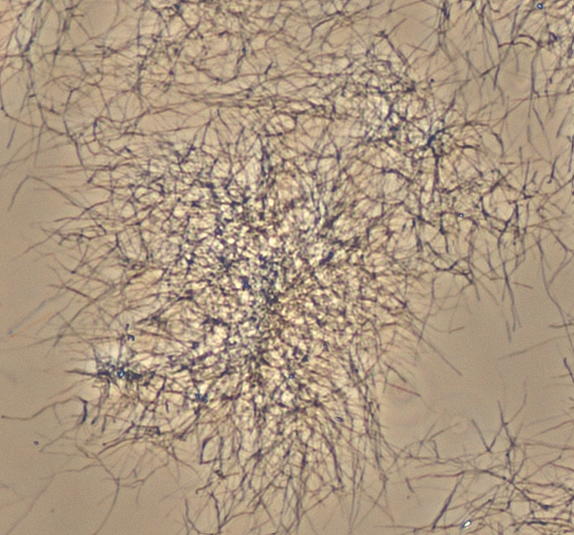 | 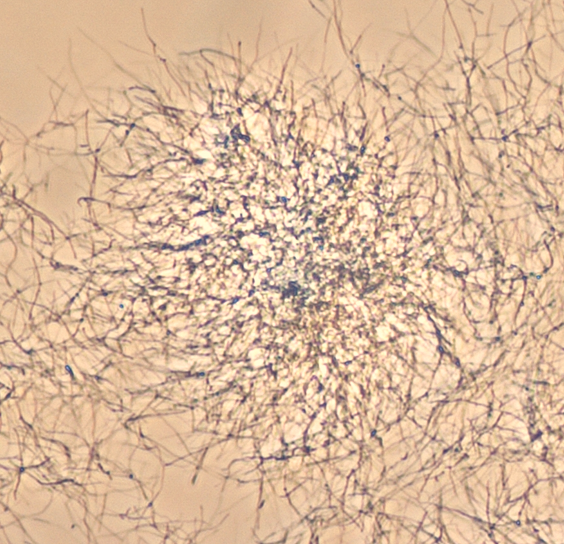 | 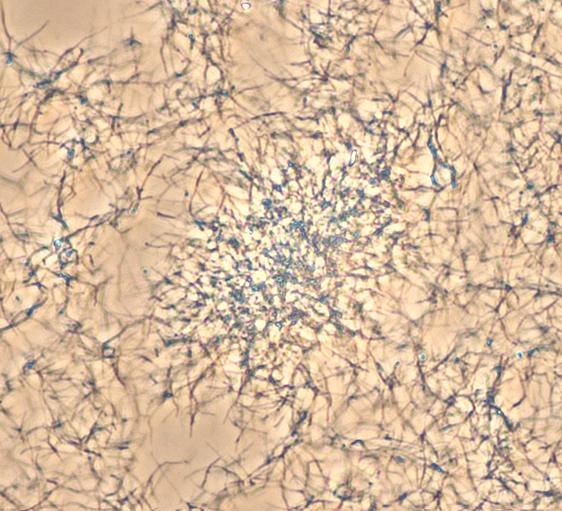 | 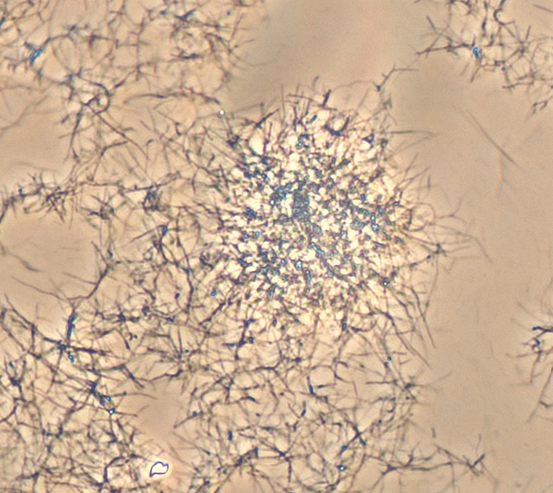 | 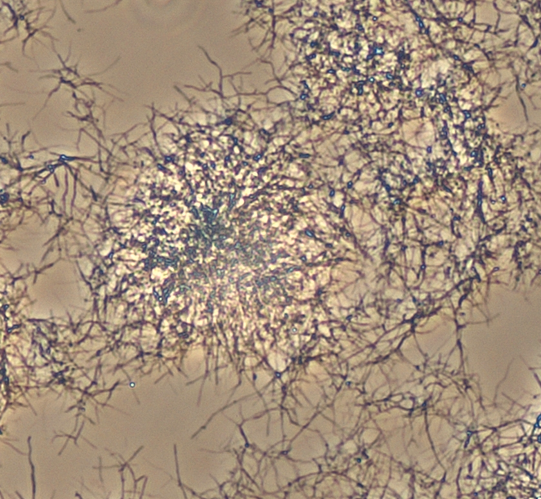 | 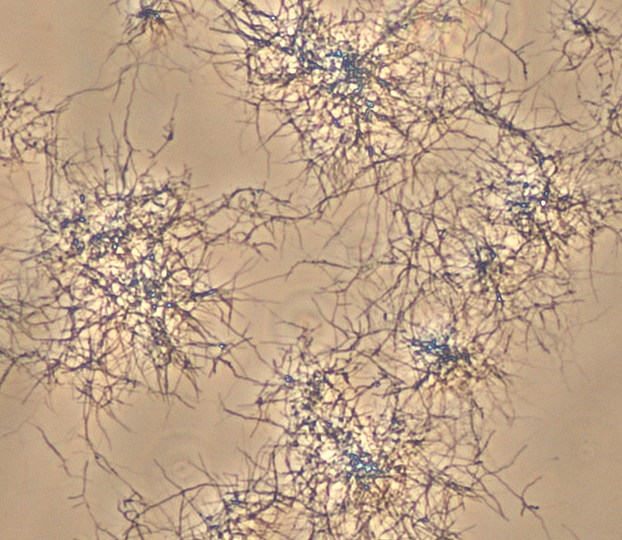  **Mycelia/Short/Thick**  **Mycelia/Short/Thick**  **Mycelia/Short/Thick**  **Mycelia/Short/Thick**  **Mycelia/Short/Thick**  **Mycelia/Long/Thick** |
| 48 FP | 1000 | 45.5 | 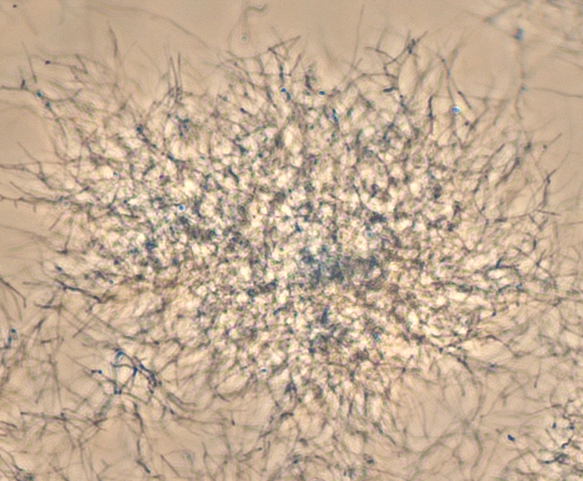  **Mycelia/Short/Thick**  **Mycelia/Short/Thick**  **Mycelia/Short/Thick**  **Mycelia/Short/Thick**  **Mycelia/Short/Thick**  **Mycelia/Long/Thick** | 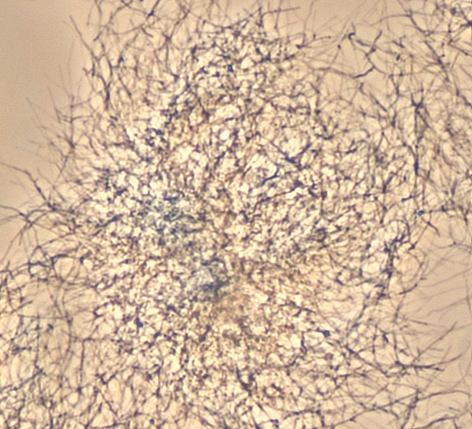 | 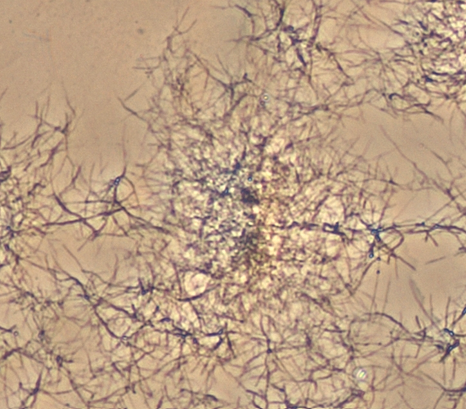 | 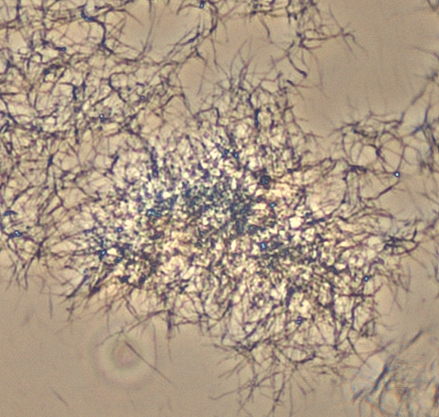 | 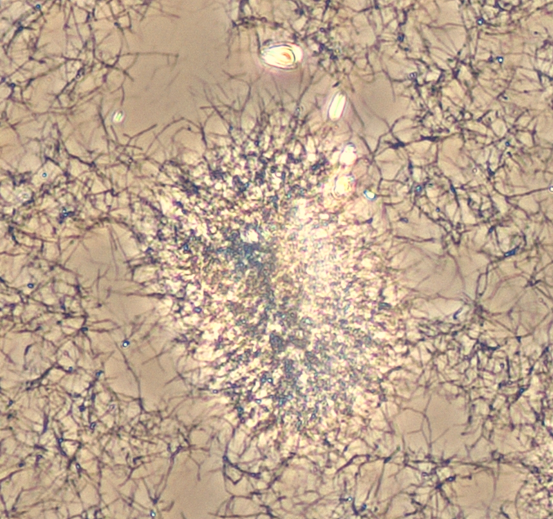 | 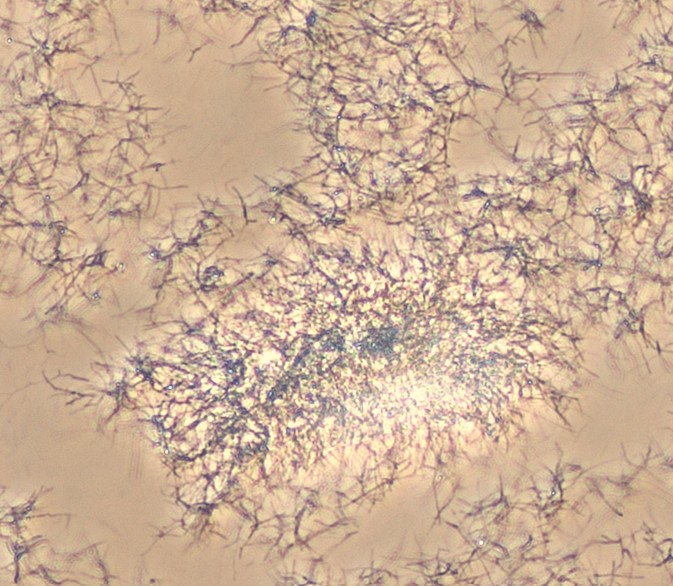 |
| 48 FP | 1100 | 56 | 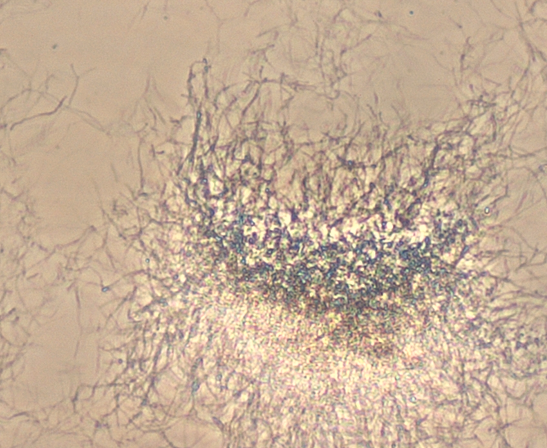  **Mycelia/Short/Thick**  **Mycelia/Short/Thick**  **Mycelia/Long/Thin**  **Mycelia/Long/Thin**  **Mycelia/Long/Thin** | 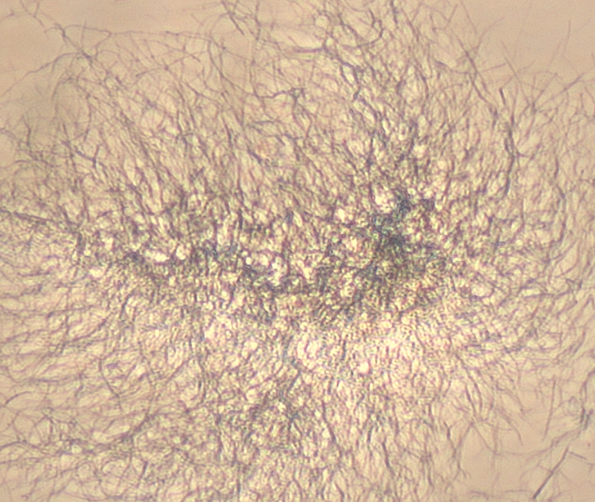 | 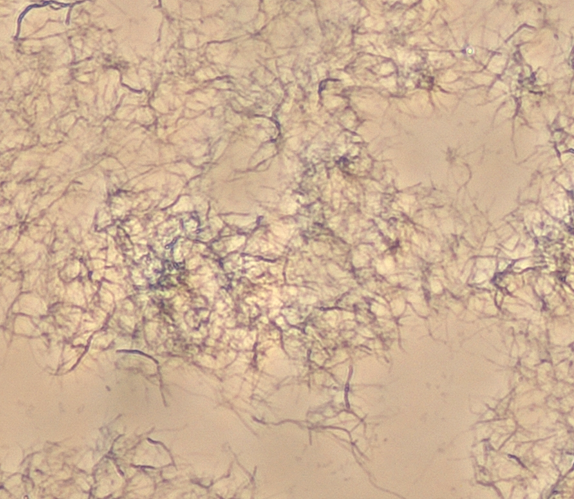 | 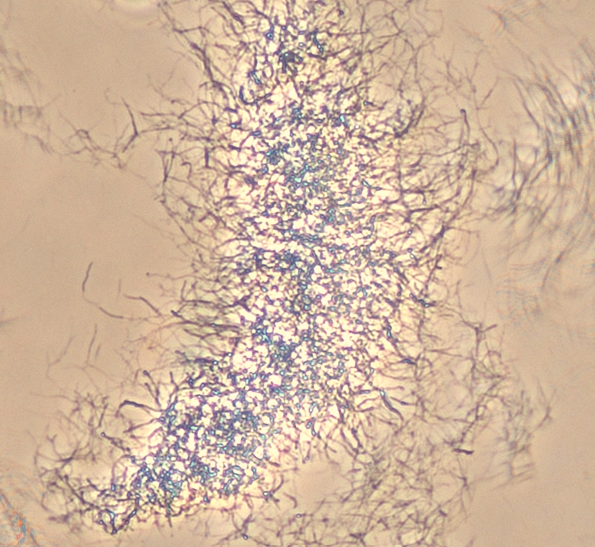 | 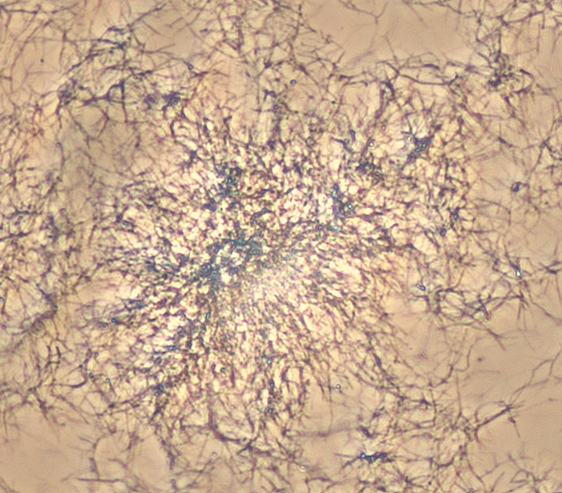 | 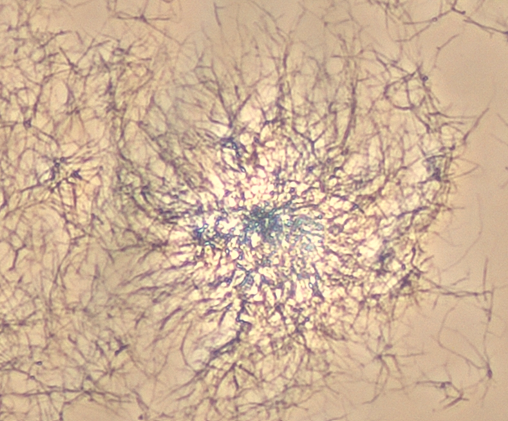  **Mycelia/Long/Thin** |
| 48 FP | 1200 | 64.5 | 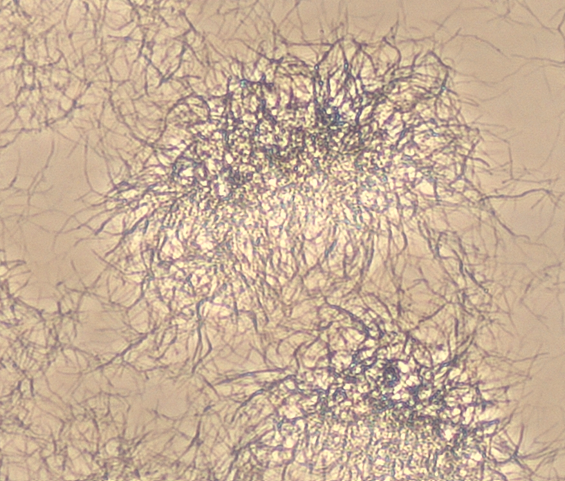  **Mycelia/Short/Thin**  **Mycelia/Short/Thin**  **Mycelia/Short/Thick**  **Mycelia/Long/Thin**  **Mycelia/Long/Thin**  **Mycelia/Long/Thin** | 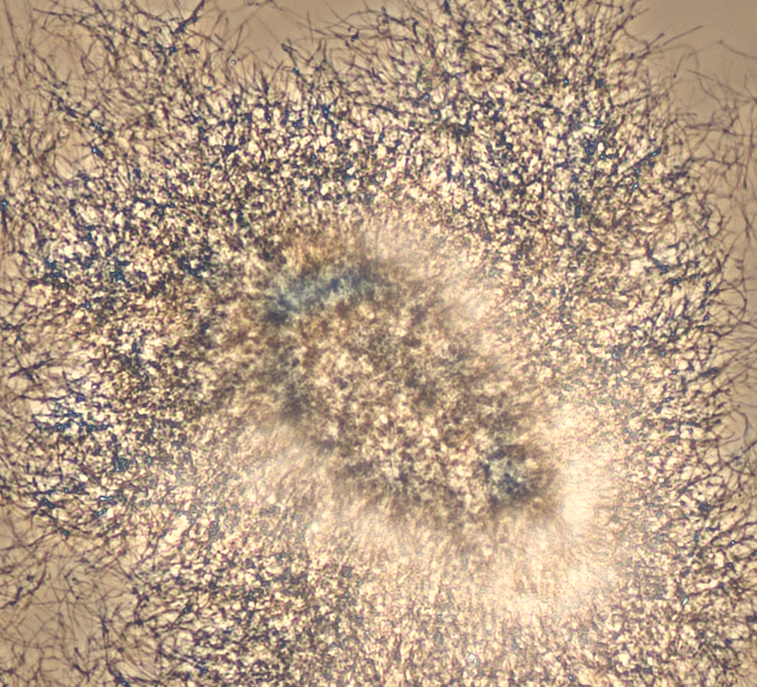 | 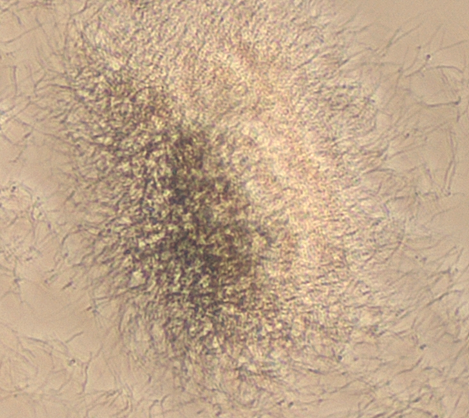 | 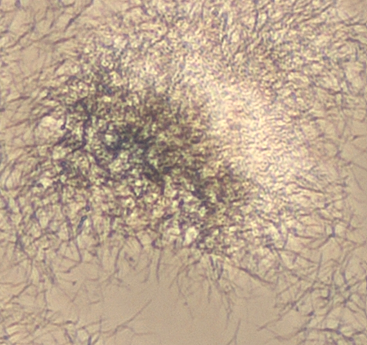 | 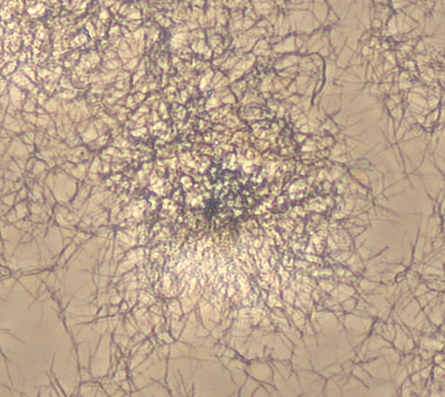 | 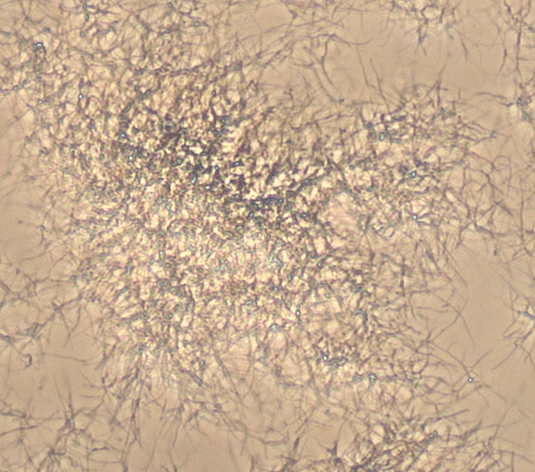 |
| 48 FP | 1300 | 75 | 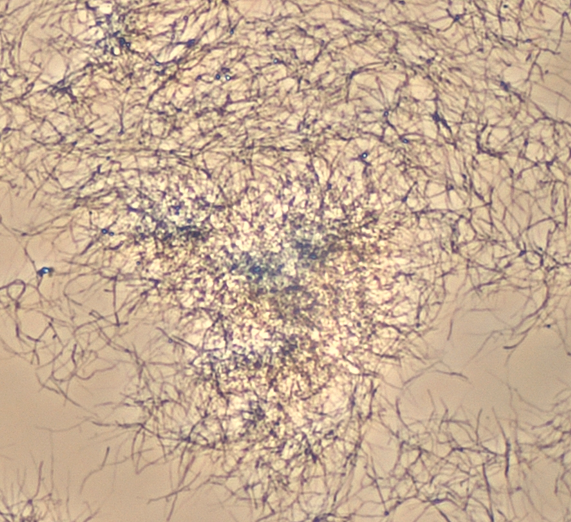  **Mycelia/Short/Thin**  **Mycelia/Short/Thick**  **Mycelia/Short/Thick**  **Mycelia/Short/Thick**  **Disperse/Short/Thick**  **Disperse/Short/Thick** | 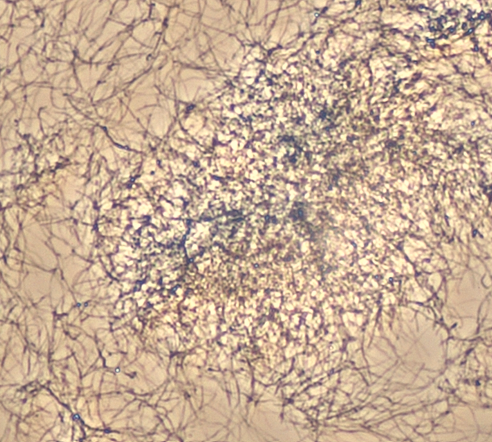 | 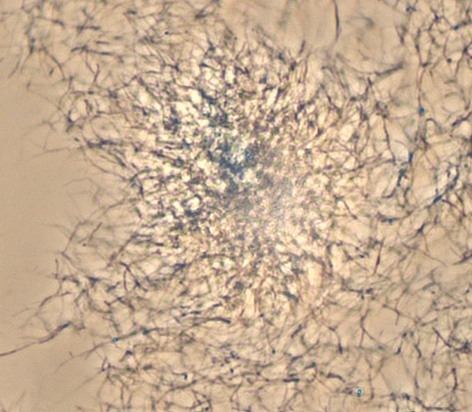 | 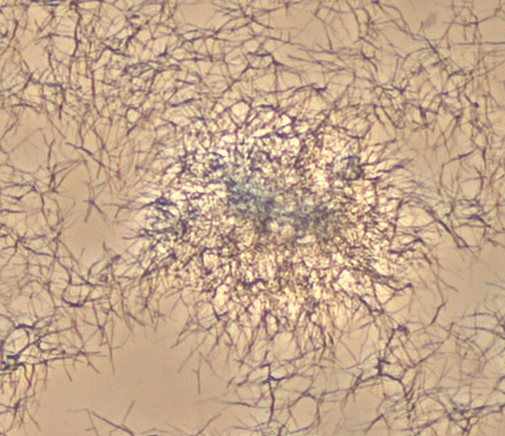 | 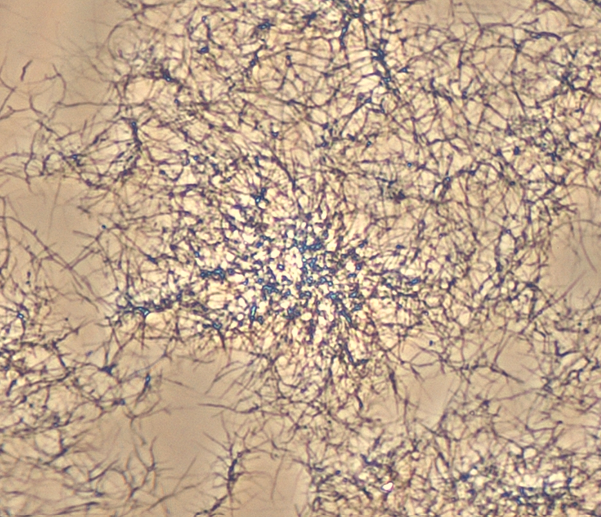 | 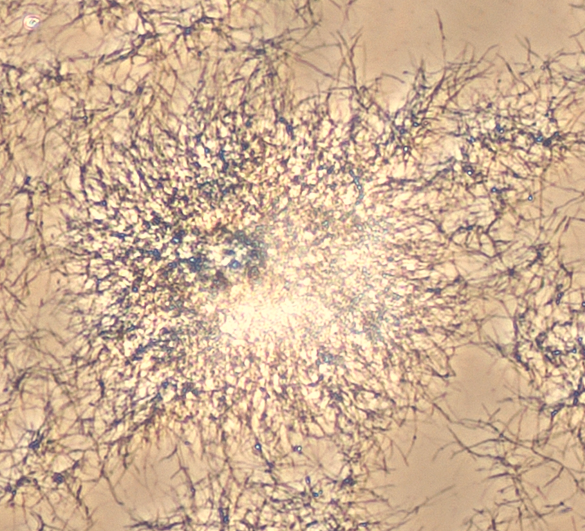 |
| 48 FP | 1400 | 87 | 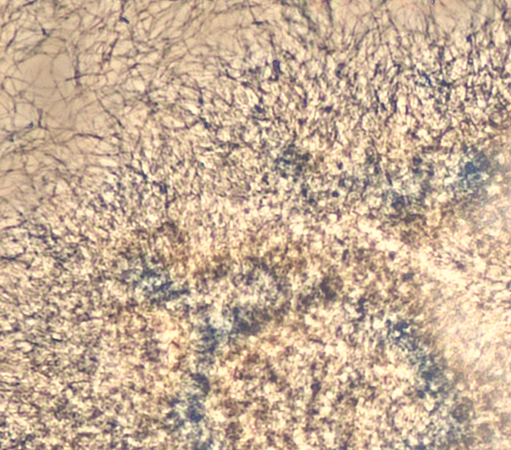  **Mycelia/Long/Thin**  **Mycelia/Long/Thin**  **Mycelia/Short/Thin**  **Mycelia/Short/Thick**  **Disperse/Short/Thick**  **Disperse/Long/Thick** | 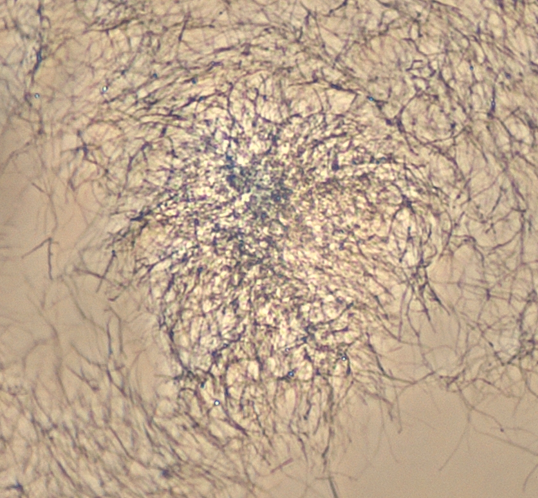 | 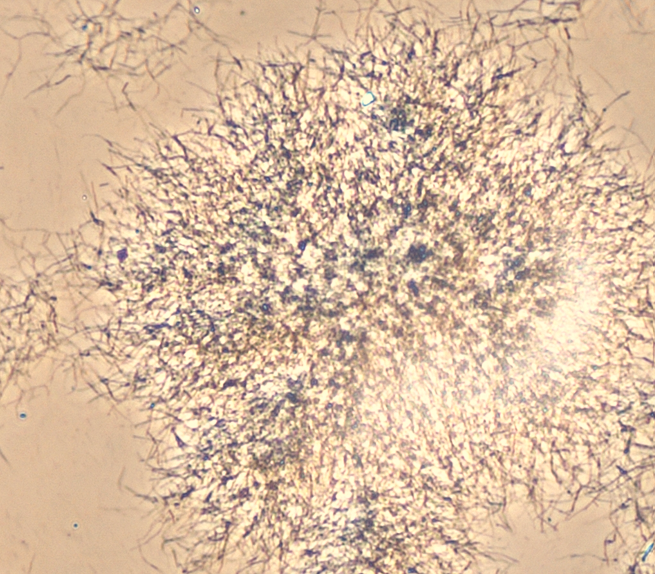 | 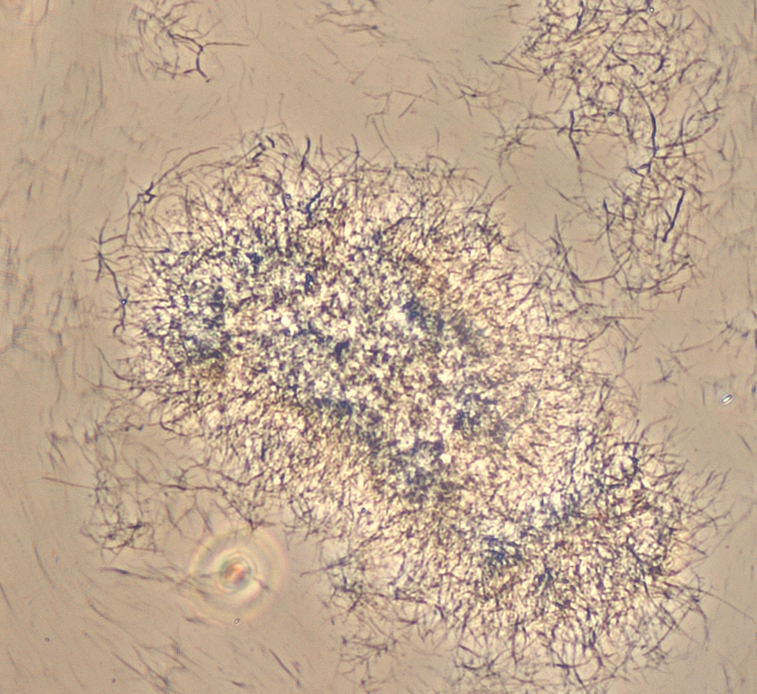 | 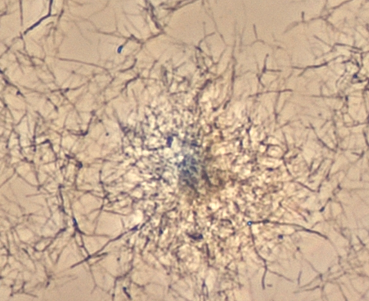 | 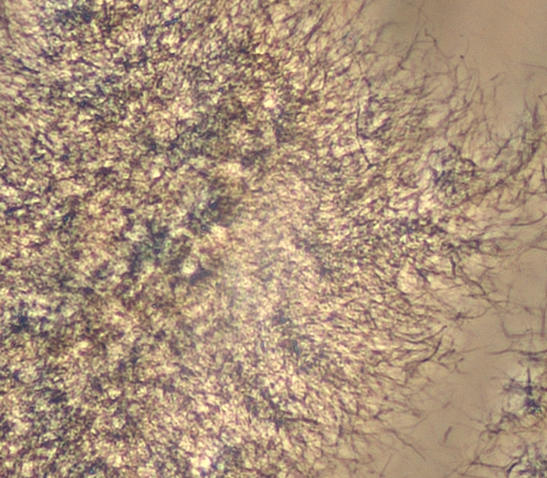 |
| BSF | 100 | 19 | 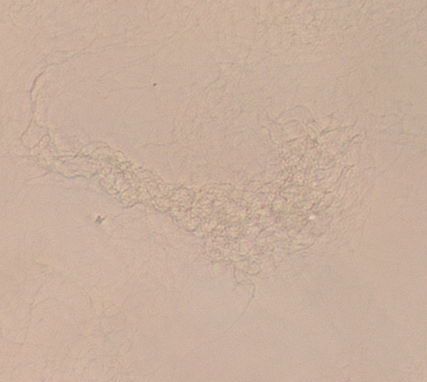  **Pellet/Short/Thick**  **Pellet/Short/Thick**  **Pellet/Long/Thin**  **Pellet/Long/Thin**  **Pellet/Long/Thin**  **Pellet/Long/Thin** | 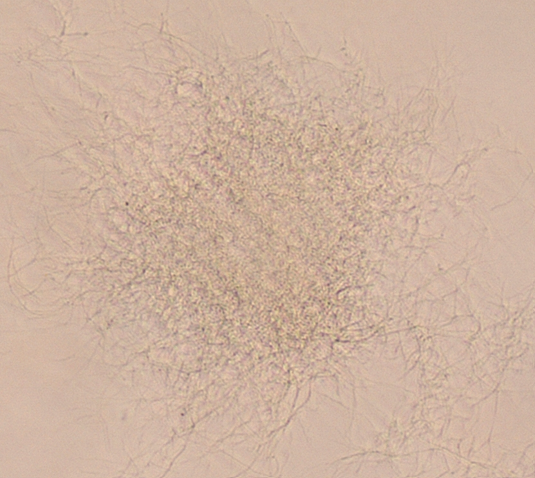 | 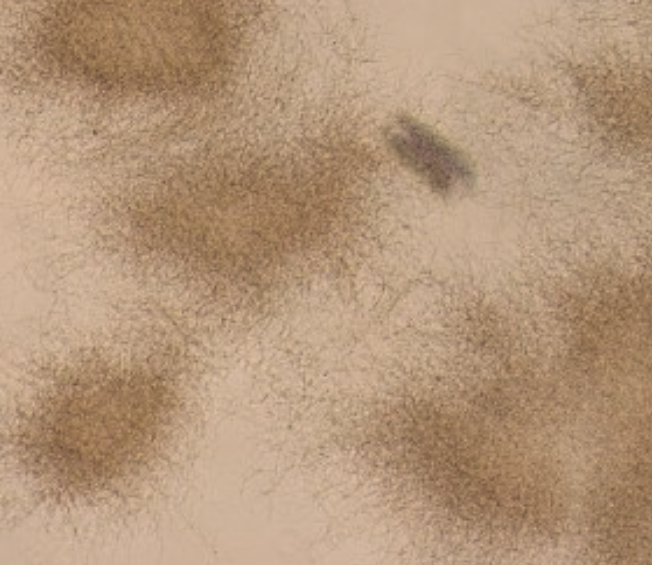 | 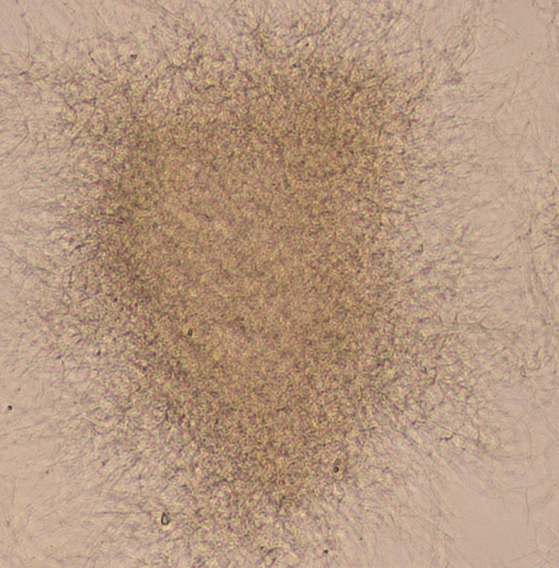 | 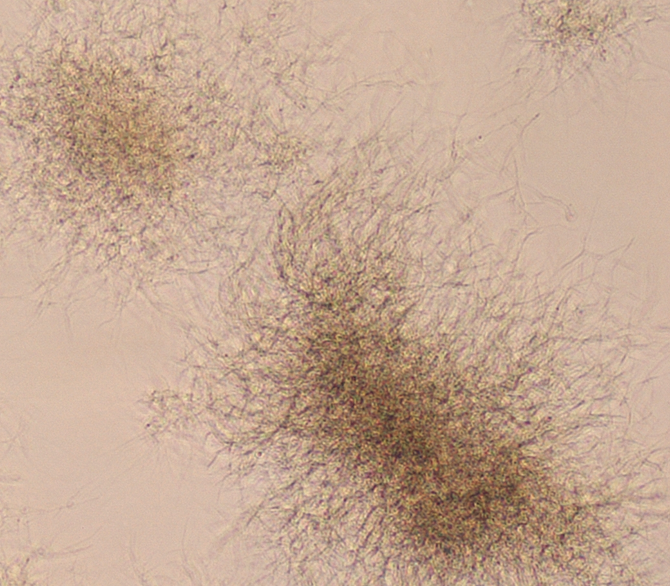 | 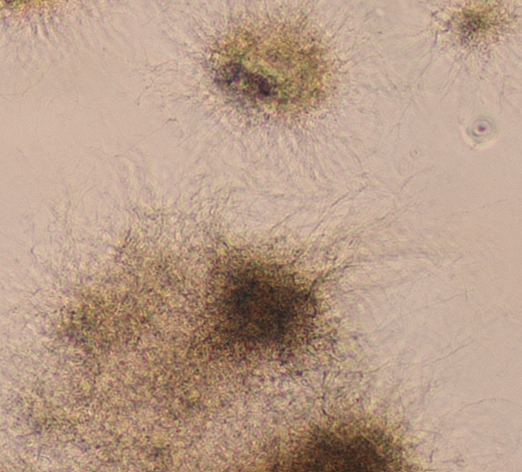 |
| BSF | 150 | 39 | 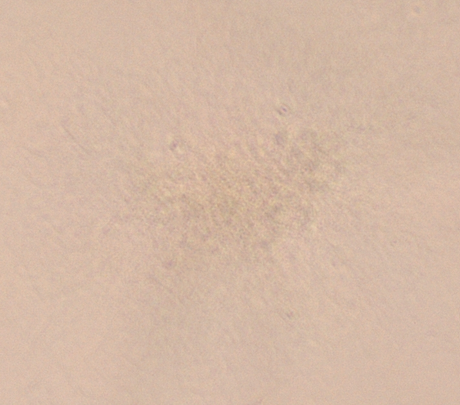  **Pellet/Long/Thin**  **Pellet/Long/Thin**  **Pellet/Long/Thin**  **Pellet/Long/Thin**  **Pellet/Short/Thick**  **Pellet/Short/Thin** | 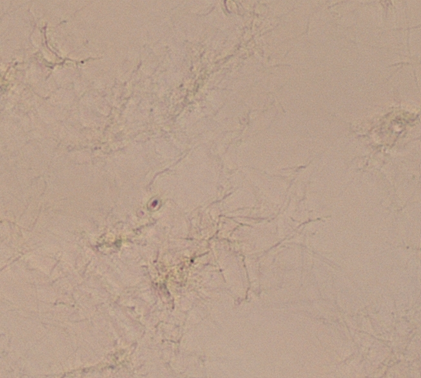 | 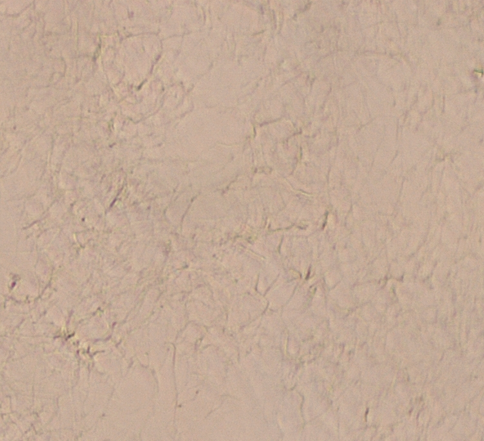 | 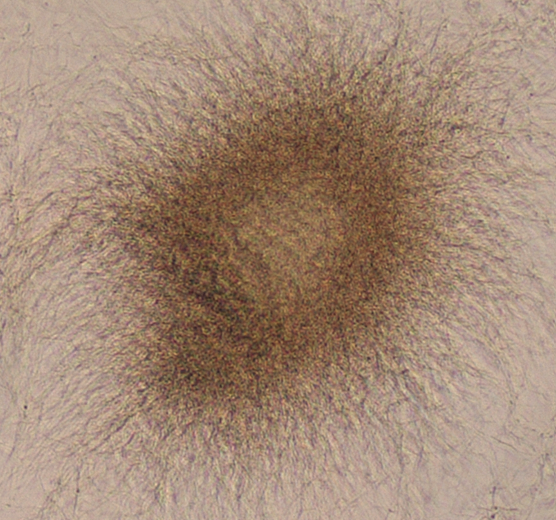 | 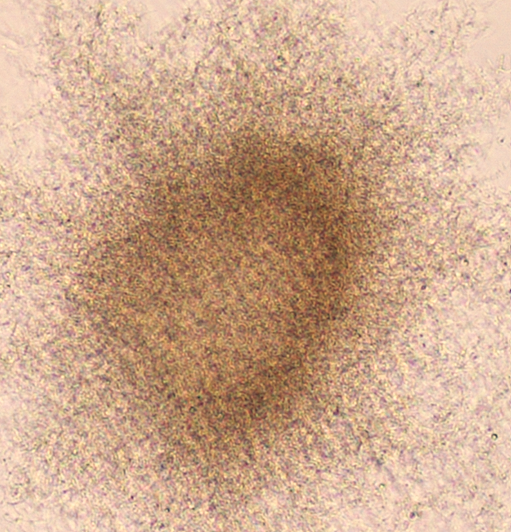 | 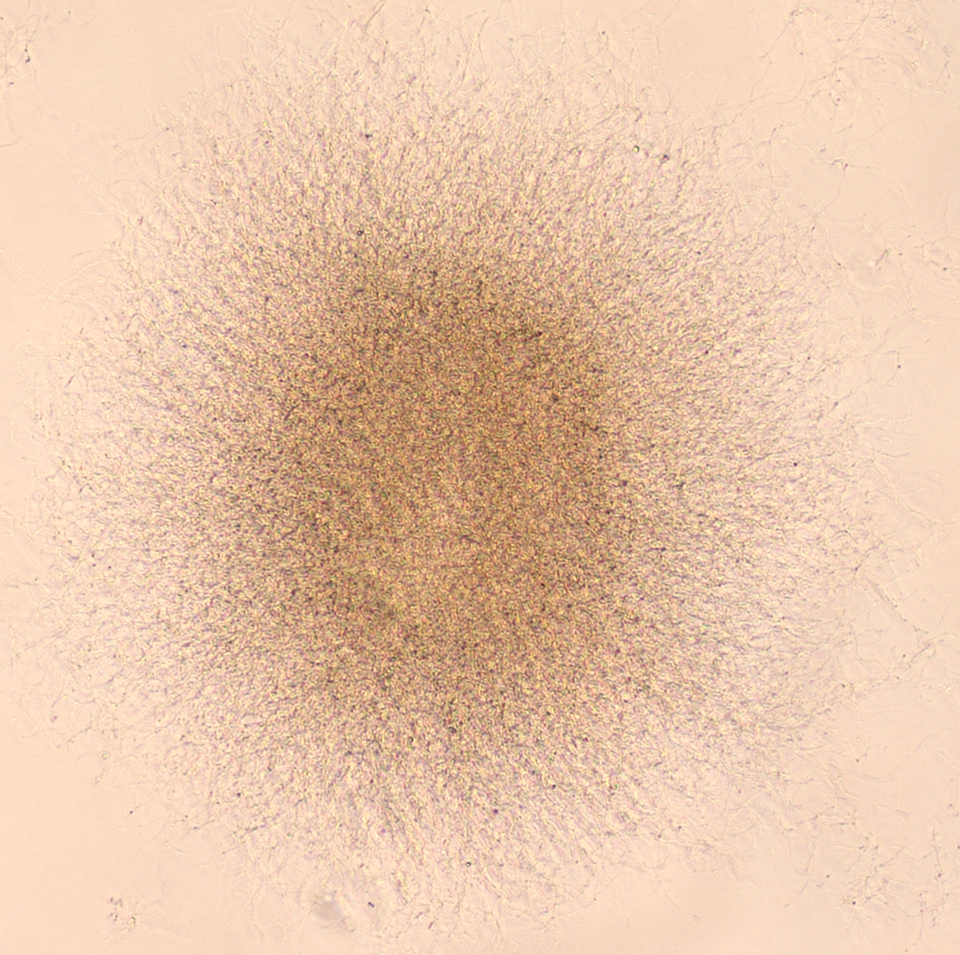 |
| BSF | 200 | 45.5 | 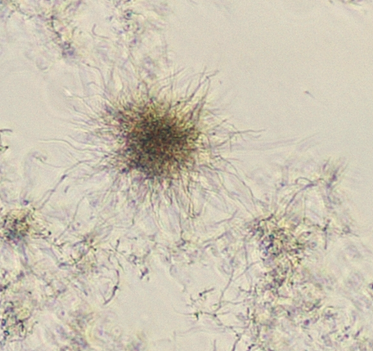  **Pellet/Long/Thin**  **Pellet/Long/Thin**  **Pellet/Short/Thin**  **Pellet/Long/Thin**  **Pellet/Short/Thick**  **Pellet/Short/Thick** | 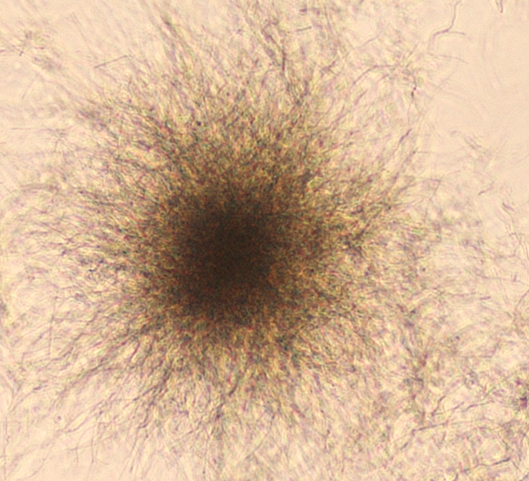 | 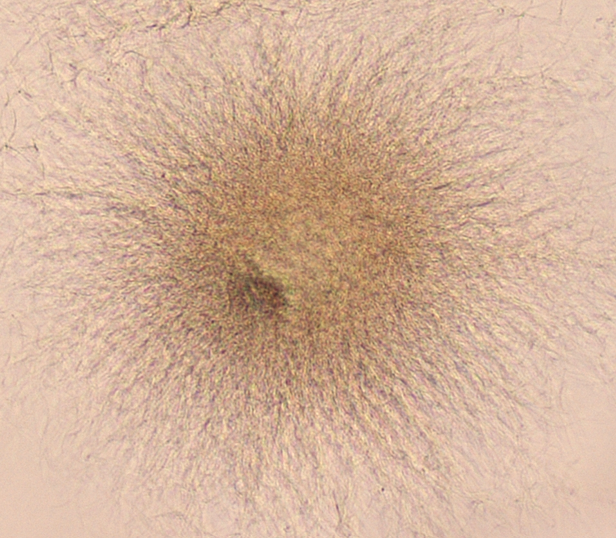 | 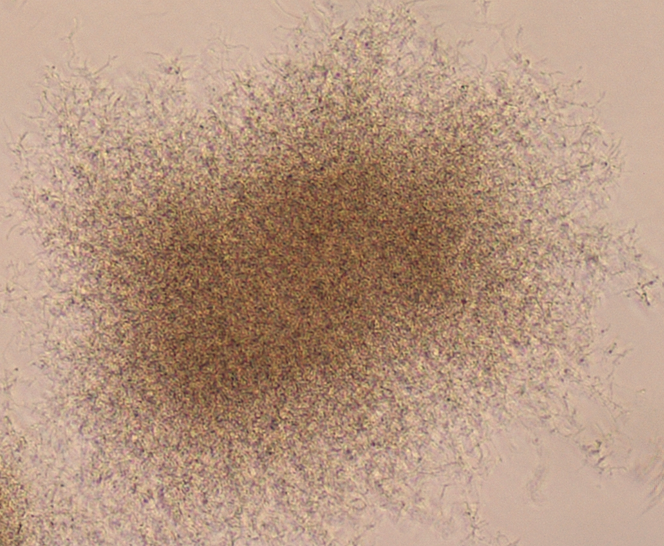 | 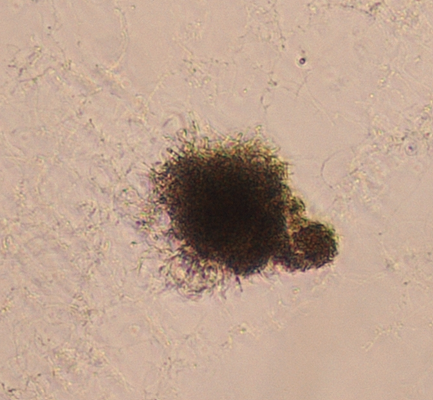 | 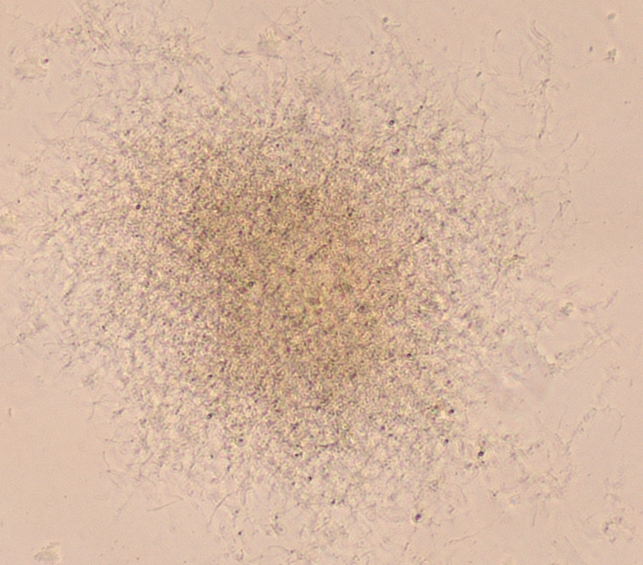 |
| BSF | 250 | 64.5 | 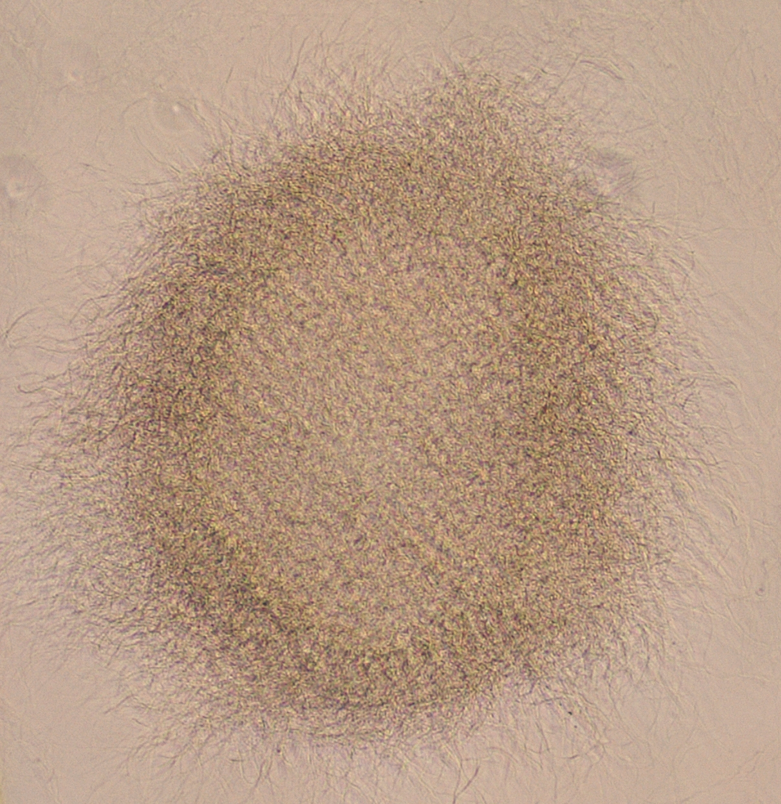 | 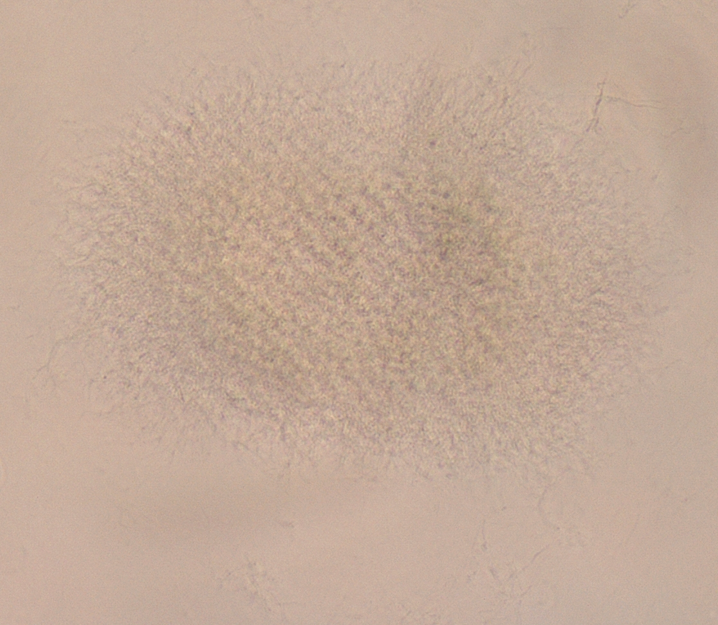 | 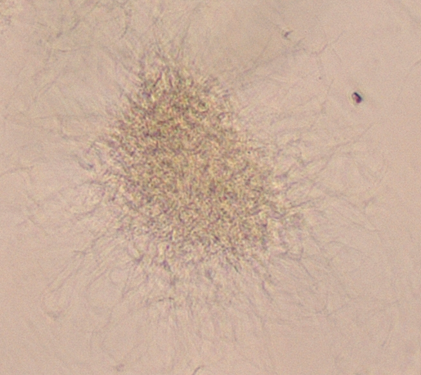 | 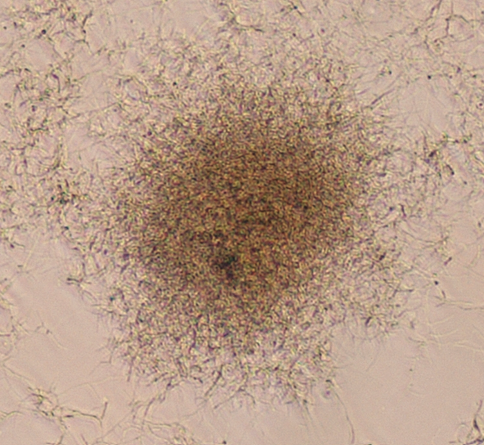 | 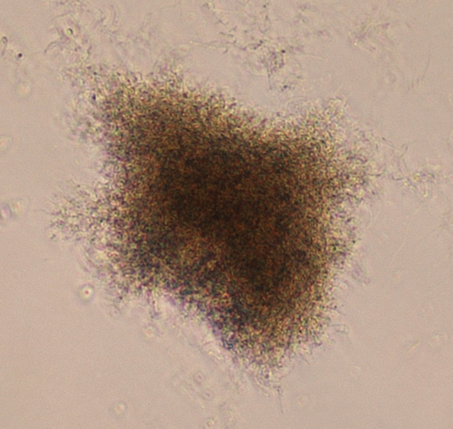 | 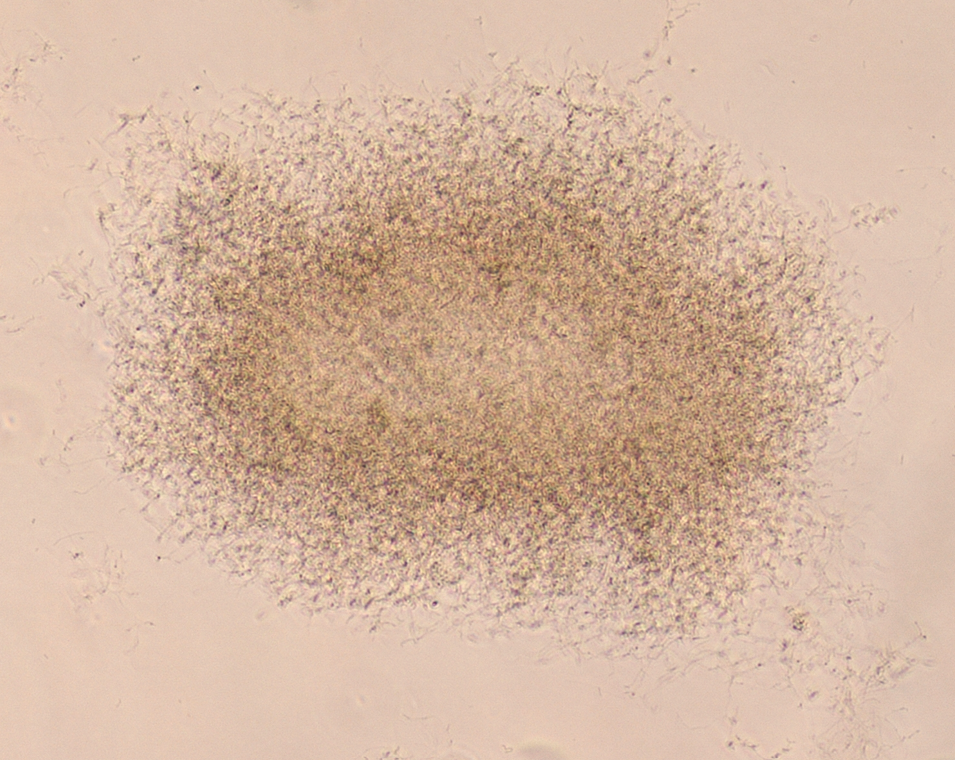 |
| BSF | 300 | 75 | 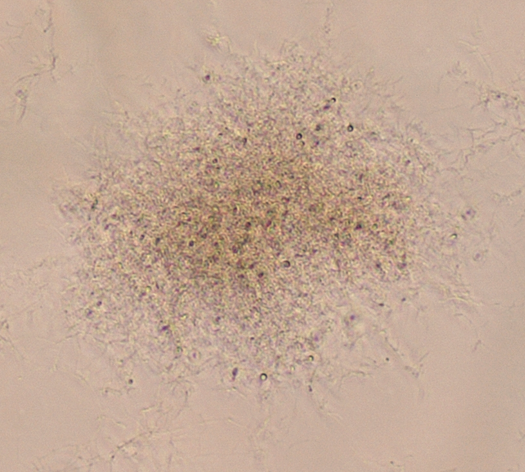  **Pellet/Long/Thin**  **Pellet/Long/Thin**  **Pellet/Short/Thin**  **Pellet/Short/Thick**  **Pellet/Short/Thick**  **Pellet/Short/Thick**  **Pellet/Long/Thin**  **Pellet/Short/Thin**  **Pellet/Short/Thin**  **Pellet/Short/Thin**  **Pellet/Short/Thick**  **Pellet/Short/Thick** | 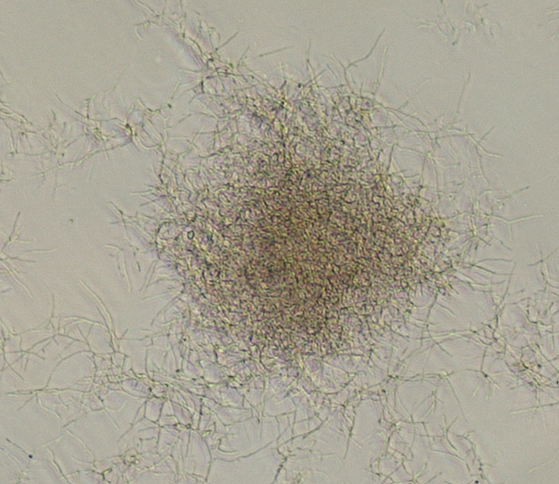 | 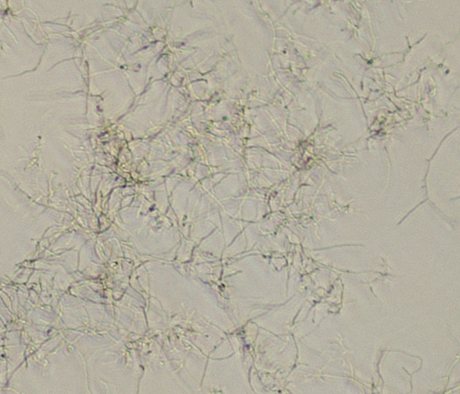 | 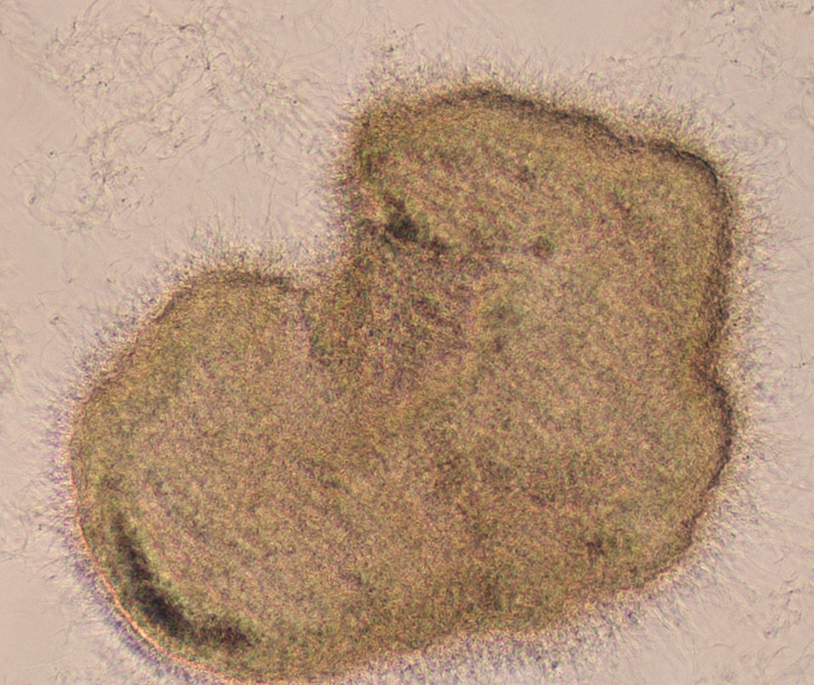 | 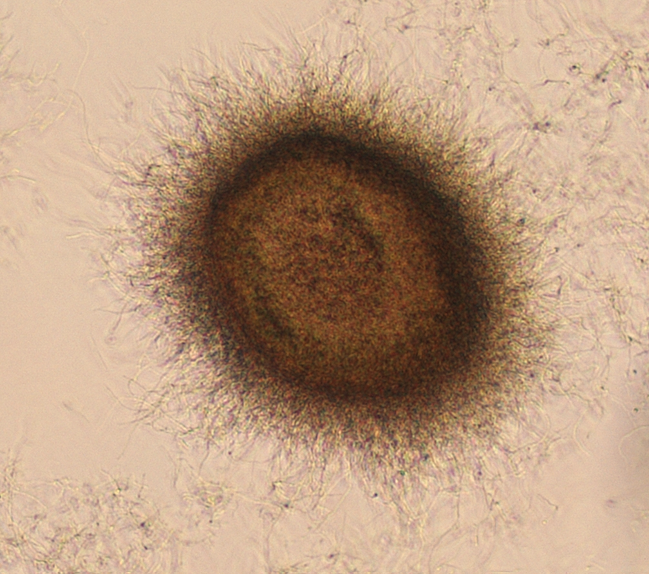 | 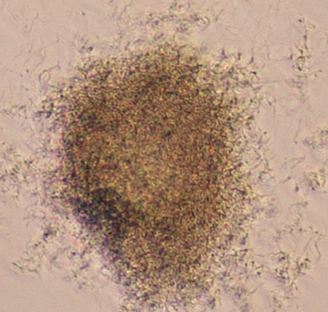 |
| STR | 500 | 25 | **-** | **-** | **-** | 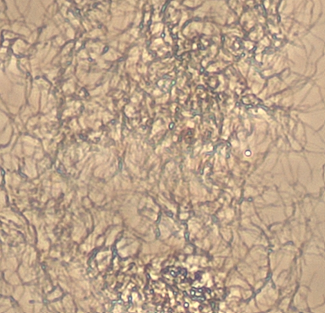  **Disperse/Short/Thick** | **-** | **-** |
| STR | 1000 | 75 | 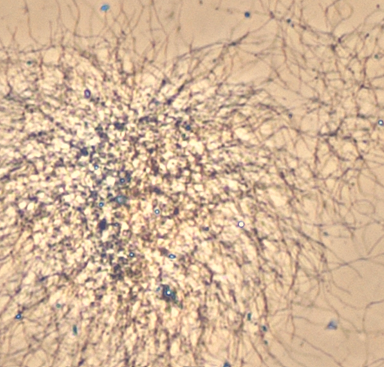 | 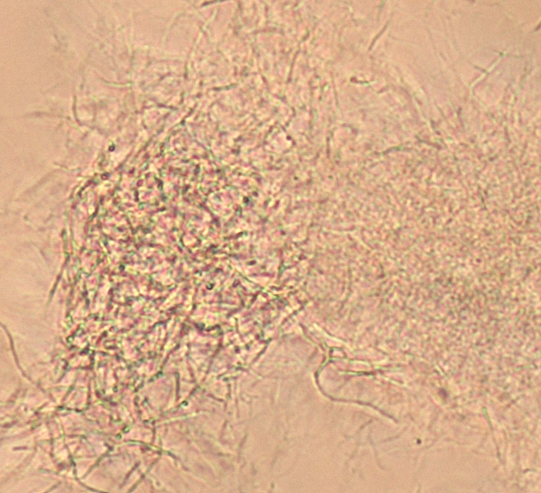 | 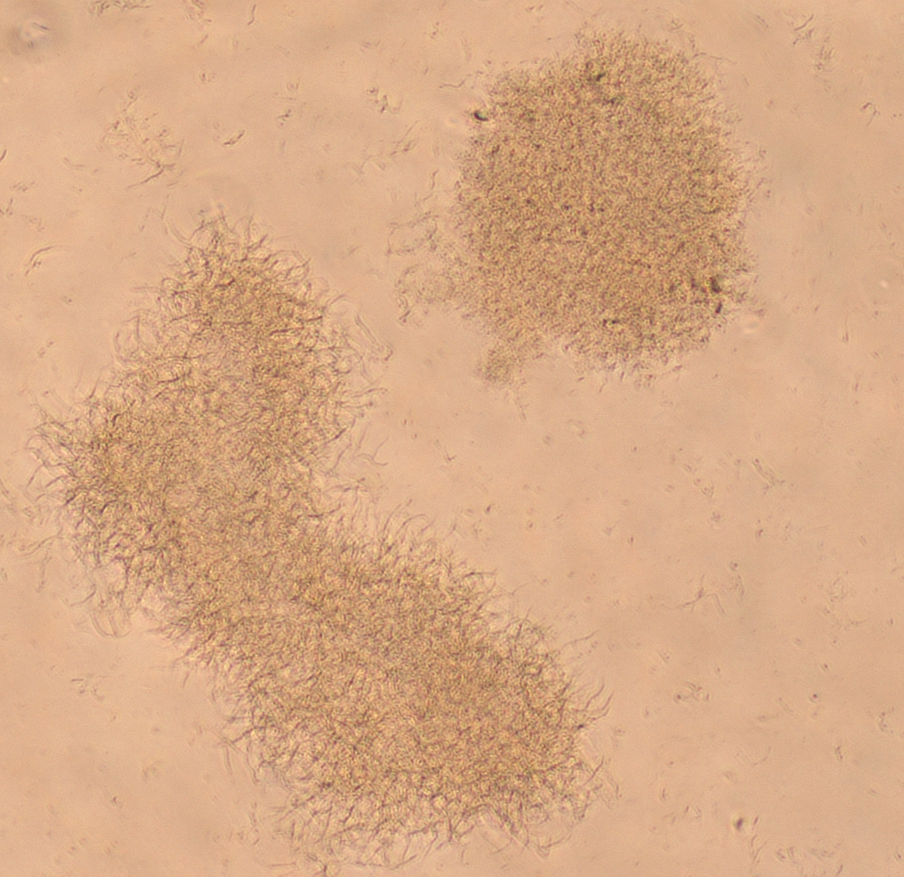 | 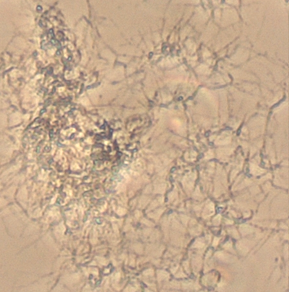 | 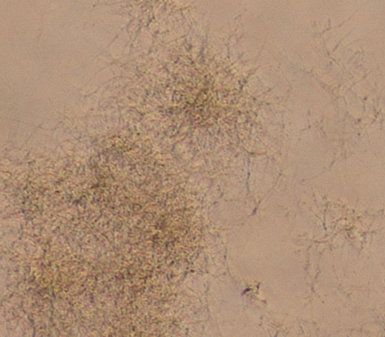 | 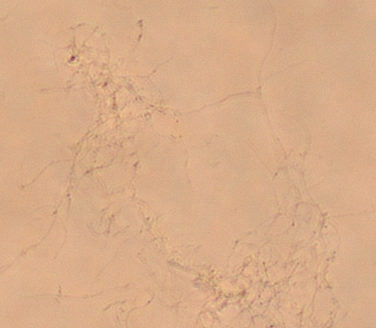 |
| STR | 1500 | 119 | **-**  **Disperse/Long/Thin**  **Disperse/Long/Thin**  **Disperse/Short/Thick**  **Pellet/Short/Thick**  **Disperse/Short/Thin**  **Disperse/Short/Thin** | **-** | **-** | 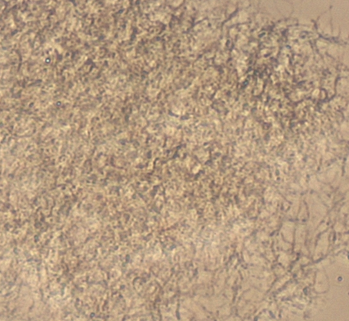  **Disperse/Short/Thick** | **-** | **-** |

^†^*48 flower plates = 48 FP, Baffles shake flask = BSF, Stirred tank bioreactor = STR.*

**Table S2:** R script used for factor analysis, including all steps with explanatory comments.

| **Comment** | **R-Code** |
| --- | --- |
| # Installation of packages | install.packages("FactoMineR")  install.packages("factoextra")  install.packages("readxl")  install.packages("ggplot2")  install.packages("ggrepel") |
| # Loading the packages | library(FactoMineR)  library(factoextra)  library(readxl)  library(ggplot2)  library(ggrepel) |
| # Loading the data from the Excel file | Data_first <- read_excel("DATA.xlsx") |
| # Extracting the "Cultivation_system" column | system_var <- Data_first[[1]] |
| # Ensuring that the "Cultivation_system" column is treated as a factor | system_var <- factor(system_var) |
| # Preparing the data for FAMD (columns 2-10) | data <- Data_first[, 2:10] |
| # The first 3 columns (after the "Cultivation_system" column) contain categorical data (treat as factors), and the last 5 columns contain numerical data. | data[1:3] <- lapply(data[1:3], factor)  data[4:9] <- lapply(data[4:9], as.numeric) |
| # Performing the FAMD | result <- FAMD(data, ncp = 25, graph = FALSE) |
| # Preparing Screeplot with eigenvalues | fviz_screeplot(result, addlabels = TRUE, ylim = c(0, 50)) +  ggtitle("Screeplot") |
| # Preparing Factor contribution to Dimension 1 | fviz_contrib(result, choice = "var", axes = 1, top = 10) +  ggtitle("Factor contribution to Dimension 1") |
| # Preparing Factor contribution to Dimension 2 | fviz_contrib(result, choice = "var", axes = 2, top = 10) +  ggtitle("Factor contribution to Dimension 2") |
| # Preparing Factor contribution to Dimension 3 | fviz_contrib(result, choice = "var", axes = 3, top = 10) +  ggtitle("Factor contribution to Dimension 3") |
| # Preparing Plot of the variables | fviz_famd_var(result, repel = TRUE, col.var = "black") +  ggtitle("Variables plot") |
| # Preparing Monoplot | fviz_famd_var(result, "quanti.var", repel = TRUE,  col.var = "black") |
| # Preparing Biplot with coloring by "Cultivation_system" and adding ellipses with a 68% confidence level | fviz_famd_ind(result,  habillage = system_var,  addEllipses = TRUE,  ellipse.level = 0.68,  repel = TRUE) |

**Table S3:** Data collected in this study and used for factor analysis, including the cultivation systems (48 FP, BSF, and STR), cell forms (dispersed, pellet, and mycelia), filament lengths (long and short), filament thickness (thin and thick), overall MFs normalized to the total amount of MFs detected, scaled-up MFs normalized to the total amount of MFs detected in the STR experiments, unique MFs normalized to the overall MFs detected under the specific cultivation condition, theoretical OTR, ethanol concentration, and CDW.

| **Cultivation_system** | **Cell_form** | **Filaments_Length** | **Filements_Tickness** | **Overall_MFs** | **Scaled-up_MFs** | **Unique_MFs** | **OTR** | **EtOH** | **CDW** |
| --- | --- | --- | --- | --- | --- | --- | --- | --- | --- |
| 48_FP | Pellet | Long | Thin | 0.22 | 0.333 | 0 | 25 | 0 | 2.3 |
| 48_FP | Pellet | Short | Thick | 0.229 | 0.346 | 0 | 25 | 1 | 2.5 |
| 48_FP | Pellet | Long | Thin | 0.244 | 0.386 | 0 | 25 | 2 | 2.6 |
| 48_FP | Pellet | Long | Thin | 0.268 | 0.379 | 0 | 25 | 3 | 2.7 |
| 48_FP | Pellet | Short | Thin | 0.127 | 0.15 | 0.02 | 25 | 4 | 2.8 |
| 48_FP | Mycelia | Long | Thin | 0.078 | 0.124 | 0 | 25 | 5 | 2.9 |
| 48_FP | Mycelia | Long | Thick | 0.373 | 0.294 | 0 | 39 | 0 | 2.4 |
| 48_FP | Mycelia | Short | Thick | 0.392 | 0.307 | 0 | 39 | 1 | 2.5 |
| 48_FP | Mycelia | Short | Thick | 0.389 | 0.366 | 0 | 39 | 2 | 2.7 |
| 48_FP | Mycelia | Short | Thick | 0.383 | 0.379 | 0 | 39 | 3 | 2.7 |
| 48_FP | Mycelia | Short | Thick | 0.205 | 0.092 | 0 | 39 | 4 | 2.9 |
| 48_FP | Mycelia | Short | Thick | 0.389 | 0.346 | 0 | 39 | 5 | 2.8 |
| 48_FP | Mycelia | Long | Thick | 0.389 | 0.275 | 0 | 45.5 | 0 | 2.4 |
| 48_FP | Mycelia | Short | Thick | 0.425 | 0.314 | 0 | 45.5 | 1 | 2.5 |
| 48_FP | Mycelia | Short | Thick | 0.434 | 0.412 | 0 | 45.5 | 2 | 2.7 |
| 48_FP | Mycelia | Short | Thick | 0.404 | 0.405 | 0 | 45.5 | 3 | 2.8 |
| 48_FP | Mycelia | Short | Thick | 0.346 | 0.359 | 0 | 45.5 | 4 | 2.9 |
| 48_FP | Mycelia | Short | Thick | 0.377 | 0.346 | 0 | 45.5 | 5 | 2.9 |
| 48_FP | Mycelia | Long | Thin | 0.349 | 0.229 | 0.01 | 56 | 0 | 2.5 |
| 48_FP | Mycelia | Long | Thin | 0.404 | 0.275 | 0 | 56 | 1 | 2.6 |
| 48_FP | Mycelia | Long | Thin | 0.482 | 0.379 | 0 | 56 | 2 | 2.8 |
| 48_FP | Mycelia | Short | Thick | 0.443 | 0.386 | 0 | 56 | 3 | 2.9 |
| 48_FP | Mycelia | Short | Thick | 0.413 | 0.373 | 0 | 56 | 4 | 2.8 |
| 48_FP | Mycelia | Long | Thin | 0.416 | 0.366 | 0 | 56 | 5 | 2.8 |
| 48_FP | Mycelia | Long | Thin | 0.34 | 0.203 | 0 | 64.5 | 0 | 2.4 |
| 48_FP | Mycelia | Long | Thin | 0.446 | 0.346 | 0 | 64.5 | 1 | 2.6 |
| 48_FP | Mycelia | Long | Thin | 0.461 | 0.366 | 0 | 64.5 | 2 | 2.7 |
| 48_FP | Mycelia | Short | Thick | 0.488 | 0.451 | 0.01 | 64.5 | 3 | 2.8 |
| 48_FP | Mycelia | Short | Thin | 0.434 | 0.379 | 0 | 64.5 | 4 | 2.8 |
| 48_FP | Mycelia | Short | Thin | 0.422 | 0.386 | 0 | 64.5 | 5 | 2.7 |
| 48_FP | Mycelia | Short | Thin | 0.301 | 0.157 | 0 | 75 | 0 | 2.4 |
| 48_FP | Mycelia | Short | Thick | 0.437 | 0.314 | 0 | 75 | 1 | 2.6 |
| 48_FP | Mycelia | Short | Thick | 0.482 | 0.386 | 0 | 75 | 2 | 2.7 |
| 48_FP | Mycelia | Short | Thick | 0.479 | 0.451 | 0 | 75 | 3 | 2.9 |
| 48_FP | Disperse | Short | Thick | 0.437 | 0.366 | 0 | 75 | 4 | 3 |
| 48_FP | Disperse | Short | Thick | 0.443 | 0.386 | 0.01 | 75 | 5 | 2.9 |
| 48_FP | Mycelia | Long | Thin | 0.322 | 0.17 | 0 | 87 | 0 | 2.5 |
| 48_FP | Mycelia | Long | Thin | 0.334 | 0.183 | 0 | 87 | 1 | 2.6 |
| 48_FP | Mycelia | Short | Thin | 0.392 | 0.248 | 0 | 87 | 2 | 2.7 |
| 48_FP | Mycelia | Short | Thick | 0.497 | 0.418 | 0 | 87 | 3 | 2.9 |
| 48_FP | Disperse | Short | Thick | 0.214 | 0.105 | 0 | 87 | 4 | 3 |
| 48_FP | Disperse | Long | Thick | 0.187 | 0.092 | 0 | 87 | 5 | 2.8 |
| BSF | Pellet | Long | Thin | 0.157 | 0.294 | 0 | 19 | 0 | 1.3 |
| BSF | Pellet | Long | Thin | 0.178 | 0.346 | 0 | 19 | 1 | 1.6 |
| BSF | Pellet | Long | Thin | 0.172 | 0.34 | 0 | 19 | 2 | 1.8 |
| BSF | Pellet | Long | Thin | 0.12 | 0.255 | 0 | 19 | 3 | 3.5 |
| BSF | Pellet | Short | Thick | 0.003 | 0.007 | 0 | 19 | 4 | 3.2 |
| BSF | Pellet | Short | Thick | 0.139 | 0.288 | 0.02 | 19 | 5 | 1.8 |
| BSF | Pellet | Long | Thin | 0.157 | 0.294 | 0 | 39 | 0 | 0.9 |
| BSF | Pellet | Long | Thin | 0.148 | 0.294 | 0 | 39 | 1 | 1.2 |
| BSF | Pellet | Long | Thin | 0.136 | 0.268 | 0 | 39 | 2 | 2.0 |
| BSF | Pellet | Long | Thin | 0.039 | 0.085 | 0 | 39 | 3 | 3.1 |
| BSF | Pellet | Short | Thick | 0.003 | 0.007 | 0 | 39 | 4 | 2.1 |
| BSF | Pellet | Short | Thin | 0.006 | 0.013 | 0 | 39 | 5 | 1.3 |
| BSF | Pellet | Long | Thin | 0.151 | 0.281 | 0 | 45.5 | 0 | 0.9 |
| BSF | Pellet | Long | Thin | 0.042 | 0.078 | 0 | 45.5 | 1 | 1.1 |
| BSF | Pellet | Short | Thin | 0.105 | 0.216 | 0 | 45.5 | 2 | 2.1 |
| BSF | Pellet | Long | Thin | 0.012 | 0.026 | 0 | 45.5 | 3 | 3.6 |
| BSF | Pellet | Short | Thick | 0.006 | 0.013 | 0 | 45.5 | 4 | 3.1 |
| BSF | Pellet | Short | Thick | 0.003 | 0.007 | 0 | 45.5 | 5 | 2.1 |
| BSF | Pellet | Long | Thin | 0.096 | 0.196 | 0 | 64.5 | 0 | 2.4 |
| BSF | Pellet | Short | Thin | 0.139 | 0.281 | 0 | 64.5 | 1 | 1.6 |
| BSF | Pellet | Short | Thin | 0.13 | 0.255 | 0 | 64.5 | 2 | 1.9 |
| BSF | Pellet | Short | Thin | 0.03 | 0.065 | 0 | 64.5 | 3 | 3.8 |
| BSF | Pellet | Short | Thick | 0.015 | 0.033 | 0 | 64.5 | 4 | 5.5 |
| BSF | Pellet | Short | Thick | 0.006 | 0.007 | 0.5 | 64.5 | 5 | 1.6 |
| BSF | Pellet | Long | Thin | 0.039 | 0.078 | 0 | 75 | 0 | 0.9 |
| BSF | Pellet | Long | Thin | 0.081 | 0.15 | 0 | 75 | 1 | 0.9 |
| BSF | Pellet | Short | Thin | 0.13 | 0.235 | 0.02 | 75 | 2 | 1.0 |
| BSF | Pellet | Short | Thick | 0.018 | 0.033 | 0 | 75 | 3 | 3.9 |
| BSF | Pellet | Short | Thick | 0.012 | 0.02 | 0 | 75 | 4 | 3.6 |
| BSF | Pellet | Short | Thick | 0.012 | 0.02 | 0 | 75 | 5 | 2.2 |
| STR | Disperse | Short | Thin | 0.295 | 0.641 | 0.06 | 75 | 0 | 1.5 |
| STR | Disperse | Short | Thin | 0.172 | 0.373 | 0.02 | 75 | 1 | 1.5 |
| STR | Pellet | Short | Thick | 0.223 | 0.484 | 0 | 75 | 2 | 1.6 |
| STR | Disperse | Short | Thick | 0.178 | 0.386 | 0 | 75 | 3 | 1.2 |
| STR | Disperse | Long | Thin | 0.063 | 0.137 | 0 | 75 | 4 | 3.4 |
| STR | Disperse | Long | Thin | 0.03 | 0.065 | 0 | 75 | 5 | 1 |
| STR | Disperse | Short | Thick | 0.178 | 0.386 | 0 | 25 | 3 | 2.2 |
| STR | Disperse | Short | Thick | 0.187 | 0.405 | 0.4 | 119 | 3 | 1.9 |

**Table S4:** Examples of the qualitative variables, namely cell form with its categories dispersed growth, pellet formation and mycelia formation, and filaments form with its categories filament lengths and filament thickness.

| **Cell form** | | **Filament form** | |
| --- | --- | --- | --- |
| Disperse | 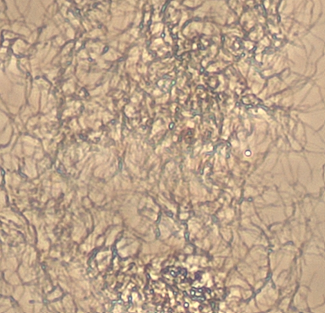 | Long and thin | 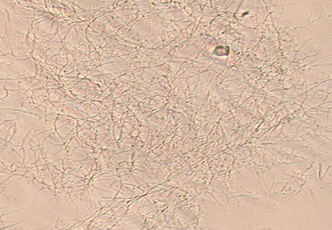 |
| Pellet | 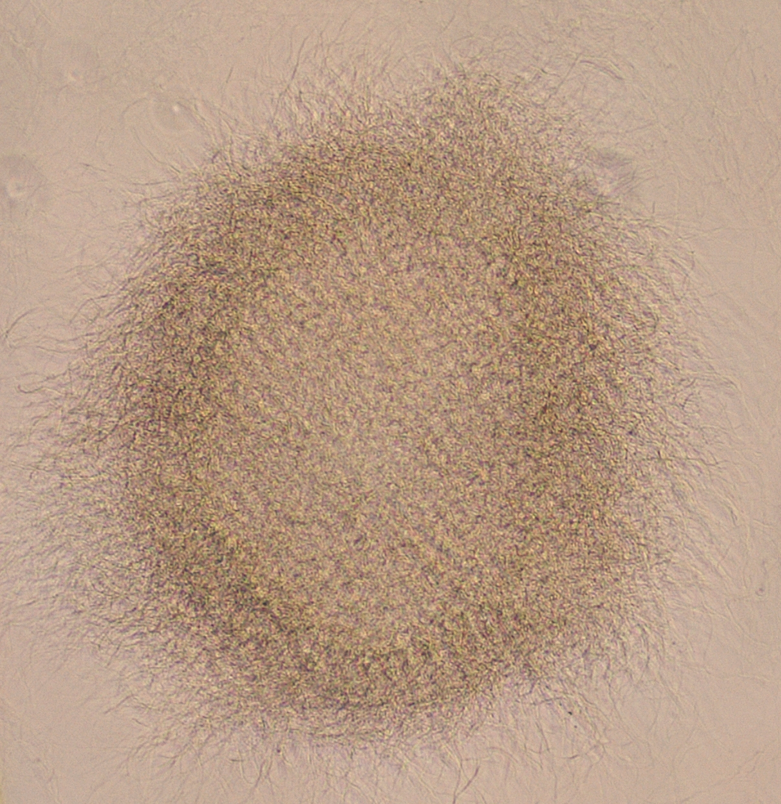 | Short and thick | 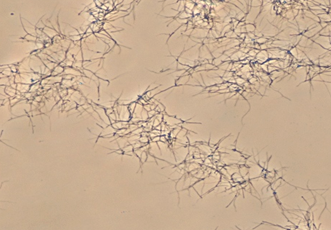 |
| Mycelia | 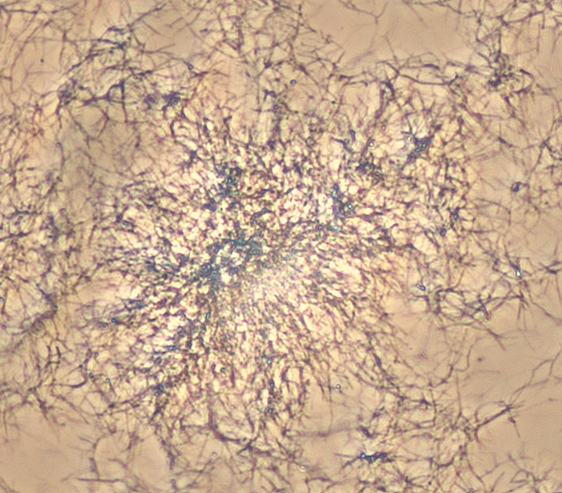 | - | - |

**Table S5:** List of all detected MFs, including the name, abbreviations, neutral mass, sum formula, adduct, maximum abundance, tier level, cultivation system and PubChem CID.

| **MF (rt_m/z)** | **Name** | **Abbreviations** | **Neutral mass [g/mol]** | **Sum formula** | **Adduct** | **Max abundance** | **Tier** | **Cultivation system**† | **PubChem CID** |
| --- | --- | --- | --- | --- | --- | --- | --- | --- | --- |
| 2.07_345.2482 | ‒ | ‒ | ‒ | ‒ | ‒ | 47502 | 4 | 48 FP, BSF, STR | ‒ |
| 2.08_281.0989 | Desferrioxamine B | Des B | 560.7 | C25H48N6O8 | [M + 2H] 2+ | 6654 | 1 | 48 FP, BSF, STR | 2973 |
| 2.08_317.2168 | ‒ | ‒ | ‒ | ‒ | ‒ | 4842 | 4 | 48 FP, BSF, STR | ‒ |
| 2.08_312.1797 | ‒ | ‒ | ‒ | ‒ | ‒ | 3459 | 4 | 48 FP, BSF | ‒ |
| 2.09_356.1305 | ‒ | ‒ | ‒ | ‒ | ‒ | 4123 | 4 | 48 FP, BSF, STR | ‒ |
| 2.1_318.1602 | ‒ | ‒ | ‒ | ‒ | ‒ | 3782 | 4 | 48 FP, BSF, STR | ‒ |
| 2.11_380.0911 | ‒ | ‒ | ‒ | ‒ | ‒ | 3228 | 4 | 48 FP, BSF, STR | ‒ |
| 2.11_204.0614 | ‒ | ‒ | ‒ | ‒ | ‒ | 2156 | 4 | 48 FP, BSF, STR | ‒ |
| 2.12_312.1656 | ‒ | ‒ | ‒ | ‒ | ‒ | 4214 | 4 | 48 FP STR | ‒ |
| 2.12_330.1947 | ‒ | ‒ | ‒ | ‒ | ‒ | 2165 | 4 | BSF, STR | ‒ |
| 2.16_245.1477 | ‒ | ‒ | ‒ | ‒ | ‒ | 14635 | 4 | 48 FP | ‒ |
| 2.16_237.1176 | ‒ | ‒ | ‒ | ‒ | ‒ | 5200 | 4 | 48 FP, BSF | ‒ |
| 2.16_600.2418 | ‒ | ‒ | ‒ | ‒ | ‒ | 4312 | 4 | BSF | ‒ |
| 2.18_585.2899 | ‒ | ‒ | ‒ | ‒ | ‒ | 1315 | 4 | BSF | ‒ |
| 2.22_314.2021 | ‒ | ‒ | ‒ | ‒ | ‒ | 6315 | 4 | 48 FP, BSF, STR | ‒ |
| 2.23_360.2029 | ‒ | ‒ | ‒ | ‒ | ‒ | 7669 | 4 | 48 FP, BSF, STR | ‒ |
| 2.24_263.1355 | ‒ | ‒ | ‒ | ‒ | ‒ | 8566 | 4 | 48 FP, BSF, STR | ‒ |
| 2.26_312.1649 | ‒ | ‒ | ‒ | ‒ | ‒ | 3887 | 4 | 48 FP STR | ‒ |
| 2.28_361.228 | ‒ | ‒ | ‒ | ‒ | ‒ | 26636 | 3 | 48 FP, BSF | ‒ |
| 2.3_268.1633 | ‒ | ‒ | ‒ | ‒ | ‒ | 11310 | 4 | 48 FP, BSF, STR | ‒ |
| 2.3_239.1463 | ‒ | ‒ | ‒ | ‒ | ‒ | 3872 | 4 | 48 FP STR | ‒ |
| 2.31_307.6377 | ‒ | ‒ | ‒ | ‒ | ‒ | 63235 | 4 | 48 FP, BSF | ‒ |
| 2.32_317.0976 | ‒ | ‒ | ‒ | ‒ | ‒ | 5509 | 4 | 48 FP | ‒ |
| 2.33_307.6402 | ‒ | ‒ | ‒ | ‒ | ‒ | 63235 | 4 | 48 FP, BSF | ‒ |
| 2.38_284.195 | ‒ | ‒ | ‒ | ‒ | ‒ | 4414 | 4 | 48 FP, BSF, STR | ‒ |
| 2.39_260.1771 | N'‑[5‑[acetyl(hydroxy)amino]pentyl]butanediamide | N-5-AHAPBA | 259.3 | C11H21N3O4 | ‒ | 7538 | 3 | 48 FP, BSF, STR | 145405924 |
| 2.41_467.2531 | L-leucyl-L-prolyl-L-glutaminyl-L-alanyl-glycine | ‒ | 484.5 | C21H36N6O7 | [M + H - H2O] + | 10827 | 3 | 48 FP, BSF, STR | 11306255 |
| 2.42_467.2598 | L-leucyl-L-prolyl-L-glutaminyl-L-alanyl-glycine | ‒ | 484.5 | C21H36N6O7 | [M + H - H2O] + | 10827 | 3 | 48 FP, BSF, STR | 11306255 |
| 2.43_423.207 | ‒ | ‒ | ‒ | ‒ | ‒ | 6003 | 4 | 48 FP, BSF, STR | ‒ |
| 2.43_289.6544 | ‒ | ‒ | ‒ | ‒ | ‒ | 2226 | 4 | 48 FP, BSF, STR | ‒ |
| 2.45_415.1591 | 3-(3-(3,4-Dihydroxy-5-(6-hydroxy-9H-purin-9-yl)tetrahydrofuran-2-yl)propanoyl)benzoic acid | ‒ | 414.4 | C19H18N4O7 |  | 11164 | 2 | BSF, STR | 135613041 |
| 2.46_314.0852 | ‒ | ‒ | ‒ | ‒ | ‒ | 3414 | 4 | BSF, STR | ‒ |
| 2.48_394.2046 | ‒ | ‒ | ‒ | ‒ | ‒ | 7396 | 4 | 48 FP, BSF, STR | ‒ |
| 2.49_419.2397 | ‒ | ‒ | ‒ | ‒ | ‒ | 4204 | 4 | 48 FP, BSF, STR | ‒ |
| 2.54_166.0843 | ‒ | ‒ | ‒ | ‒ | ‒ | 13438 | 4 | 48 FP, BSF, STR | ‒ |
| 2.55_243.6689 | ‒ | ‒ | ‒ | ‒ | ‒ | 16409 | 4 | 48 FP | ‒ |
| 2.55_431.2424 | L-phenylalanyl-L-leucyl-L-histidine hydroxyamide | ‒ | 430.5 | C21H30N6O4 |  | 1831 | 3 | BSF, STR | 70697494 |
| 2.56_366.1953 | ‒ | ‒ | ‒ | ‒ | ‒ | 1927 | 4 | BSF, STR | ‒ |
| 2.58_408.2194 | ‒ | ‒ | ‒ | ‒ | ‒ | 12542 | 4 | 48 FP, BSF, STR | ‒ |
| 2.58_486.3365 | ‒ | ‒ | ‒ | ‒ | ‒ | 15933 | 4 | 48 FP | ‒ |
| 2.61_257.1911 | ‒ | ‒ | ‒ | ‒ | ‒ | 156349 | 4 | 48 FP | ‒ |
| 2.61_513.376 | N'-[5-[[4-(5-acetamidopentylamino)-4-oxobutanoyl]amino]pentyl]-N-(5-aminopentyl)butanediamide | N-5-AOA | 512.7 | C25H48N6O5 |  | 28460 | 3 | 48 FP | 134335192 |
| 2.61_364.1117 | ‒ | ‒ | ‒ | ‒ | ‒ | 7791 | 4 | 48 FP, BSF, STR | ‒ |
| 2.61_485.3436 | Desferrioxamine mesilate impurity I | Des mesilate impurity I | 502.6 | C23H46N6O6 | [M + H - H2O] + | 19641 | 3 | 48 FP | 102209481 |
| 2.61_369.2475 | ‒ | ‒ | ‒ | ‒ | ‒ | 12840 | 4 | 48 FP | ‒ |
| 2.67_270.1795 | ‒ | ‒ | ‒ | ‒ | ‒ | 3996 | 4 | 48 FP, BSF, STR | ‒ |
| 2.67_360.127 | ‒ | ‒ | ‒ | ‒ | ‒ | 5605 | 4 | 48 FP, BSF, STR | ‒ |
| 2.69_212.1075 | ‒ | ‒ | ‒ | ‒ | ‒ | 3278 | 4 | 48 FP, BSF, STR | ‒ |
| 2.69_301.1741 | ‒ | ‒ | ‒ | ‒ | ‒ | 3467 | 4 | 48 FP | ‒ |
| 2.7_358.1893 | ‒ | ‒ | ‒ | ‒ | ‒ | 5417 | 4 | 48 FP, BSF, STR | ‒ |
| 2.7_327.6927 | ‒ | ‒ | ‒ | ‒ | ‒ | 3712 | 4 | BSF | ‒ |
| 2.71_251.6667 | ‒ | ‒ | ‒ | ‒ | ‒ | 9171 | 4 | 48 FP, BSF | ‒ |
| 2.71_561.361 | Desferrioxamine B | Des B | 560.7 | C25H48N6O8 |  | 3060 | 1 | 48 FP, BSF | 2973 |
| 2.71_444.2806 | ‒ | ‒ | ‒ | ‒ | ‒ | 18855 | 4 | 48 FP | ‒ |
| 2.72_325.092 | ‒ | ‒ | ‒ | ‒ | ‒ | 5398 | 4 | 48 FP, BSF, STR | ‒ |
| 2.72_561.3506 | Desferrioxamine B | Des B | 560.7 | C25H48N6O8 | ‒ | 2992 | 1 | 48 FP, BSF | 2973 |
| 2.74_265.1879 | N-(5-acetamidopentyl)-N'-[5-[[4-[5-(hydroxyamino)pentylamino]-4-oxobutanoyl]amino]pentyl]butanediamide | N-5-AHPOPB | 528.7 | C25H48N6O6 | [M + 2H] 2+ | 201328 | 3 | 48 FP, BSF | 88633559 |
| 2.74_502.3251 | IC202D | ‒ | 501.6 | C23H43N5O7 | ‒ | 12013 | 3 | 48 FP, BSF, STR | 10577363 |
| 2.75_414.1374 | ‒ | ‒ | ‒ | ‒ | ‒ | 30141 | 4 | 48 FP, BSF, STR | ‒ |
| 2.76_369.1987 | ‒ | ‒ | ‒ | ‒ | ‒ | 25973 | 4 | 48 FP, BSF, STR | ‒ |
| 2.76_501.3379 | ‒ | ‒ | ‒ | ‒ | ‒ | 23421 | 4 | 48 FP | ‒ |
| 2.76_529.3723 | N-(5-acetamidopentyl)-N'-[5-[[4-[5-(hydroxyamino)pentylamino]-4-oxobutanoyl]amino]pentyl]butanediamide | N-5-AHPOPB | 528.7 | C25H48N6O6 | ‒ | 55360 | 3 | 48 FP | 88633559 |
| 2.76_316.1854 | L-leucyl-L-threonyl-L-threonine | ‒ | 333.38 | C14H27N3O6 | [M + H - H2O] + | 23497 | 3 | 48 FP, BSF | 145456527 |
| 2.76_428.2835 | ‒ | ‒ | ‒ | ‒ | ‒ | 7905 | 4 | 48 FP | ‒ |
| 2.77_200.0907 | ‒ | ‒ | ‒ | ‒ | ‒ | 18577 | 4 | 48 FP, BSF, STR | ‒ |
| 2.78_529.3641 | N-(5-acetamidopentyl)-N'-[5-[[4-[5-(hydroxyamino)pentylamino]-4-oxobutanoyl]amino]pentyl]butanediamide | N-5-AHPOPB | 528.7 | C25H48N6O6 |  | 43371 | 3 | 48 FP | 88633559 |
| 2.81_303.2369 | ‒ | ‒ | ‒ | ‒ | ‒ | 62688 | 4 | 48 FP, BSF, STR | ‒ |
| 2.81_227.1368 | ‒ | ‒ | ‒ | ‒ | ‒ | 37826 | 4 | 48 FP, BSF, STR | ‒ |
| 2.81_180.099 | ‒ | ‒ | ‒ | ‒ | ‒ | 14287 | 4 | 48 FP, BSF, STR | ‒ |
| 2.82_414.1207 | ‒ | ‒ | ‒ | ‒ | ‒ | 7369 | 4 | 48 FP, BSF | ‒ |
| 2.83_518.3158 | 1k2v | ‒ | 495.6 | C21H45N5O8 | [M + Na] + | 45630 | 3 | 48 FP, BSF, STR | 23585928 |
| 2.83_259.6635 | ‒ | ‒ | ‒ | ‒ | ‒ | 21536 | 4 | 48 FP, BSF, STR | ‒ |
| 2.83_200.0909 | ‒ | ‒ | ‒ | ‒ | ‒ | 18577 | 4 | 48 FP, BSF, STR | ‒ |
| 2.83_417.2283 | ‒ | ‒ | ‒ | ‒ | ‒ | 3324 | 4 | 48 FP, BSF, STR | ‒ |
| 2.84_273.1848 | N-[5-[[4-[5-[acetyl(hydroxy)amino]pentylamino]-4-oxobutanoyl]-hydroxyamino]pentyl]-N'-hydroxy-N'-pentylbutanediamide | N-5-AOHHP | 545.7 | C25H47N5O8 | [M + 2H] 2+ | 491420 | 3 | 48 FP, BSF, STR | 137444661 |
| 2.84_289.1795 | N-Hydroxydesferrioxamine B | Hydroxy-Des B | 576.7 | C25H48N6O9 | [M + 2H] 2+ | 14531 | 3 | 48 FP, BSF, STR | 44149952 |
| 2.84_545.3675 | N'-[5-[acetyl(hydroxy)amino]pentyl]-N-[5-[[4-[5-aminopentyl(hydroxy)amino]-4-oxobutanoyl]amino]pentyl]butanediamide | N-5-AAO | 544.7 | C25H48N6O7 |  | 136437 | 3 | 48 FP, BSF, STR | 60019158 |
| 2.84_518.3234 | 1k2v | ‒ | 495.6 | C21H45N5O8 | [M + Na] + | 45630 | 3 | 48 FP, BSF, STR | 23585928 |
| 2.84_303.2383 | ‒ | ‒ | ‒ | ‒ | ‒ | 62688 | 4 | 48 FP, BSF, STR | ‒ |
| 2.84_577.3532 | N-Hydroxy-desferrioxamine B | Hydroxy-Des B | 576.7 | C25H48N6O9 | ‒ | 15632 | 3 | 48 FP, BSF, STR | 44149952 |
| 2.85_545.3599 | N'-[5-[acetyl(hydroxy)amino]pentyl]-N-[5-[[4-[5-aminopentyl(hydroxy)amino]-4-oxobutanoyl]amino]pentyl]butanediamide | N-5-AAO | 544.7 | C25H48N6O7 | ‒ | 121649 | 3 | 48 FP, BSF, STR | 60019158 |
| 2.85_517.3256 | N'-(5-aminopentyl)-N-[5-[[4-[5-(dihydroxyamino)pentylamino]-4-oxobutanoyl]-hydroxyamino]pentyl]-N'-hydroxybutanediamide | N-5-ADOHH | 534.6 | C23H46N6O8 | [M + H - H2O] + | 32302 | 3 | 48 FP, BSF, STR | 163123947 |
| 2.86_460.2751 | ‒ | ‒ | ‒ | ‒ | ‒ | 20413 | 4 | 48 FP, BSF | ‒ |
| 2.87_517.3319 | ‒ | ‒ | ‒ | ‒ | ‒ | 32302 | 4 | 48 FP, BSF, STR | ‒ |
| 2.88_227.1374 | ‒ | ‒ | ‒ | ‒ | ‒ | 37826 | 4 | 48 FP, BSF, STR | ‒ |
| 2.91_314.1663 | ‒ | ‒ | ‒ | ‒ | ‒ | 22007 | 4 | 48 FP, BSF, STR | ‒ |
| 2.91_499.3239 | ‒ | ‒ | ‒ | ‒ | ‒ | 19273 | 4 | 48 FP | ‒ |
| 2.91_371.2624 | N,N'-bis(4-acetamidobutyl)hexanediamide | N-BAH | 370.5 | C18H34N4O4 | ‒ | 9707 | 3 | 48 FP | 140945982 |
| 2.92_343.2309 | ‒ | ‒ | ‒ | ‒ | ‒ | 27315 | 4 | 48 FP | ‒ |
| 2.92_457.2586 | L-phenylalanyl-L-isoleucyl-L-arginine | ‒ | 434.5 | C21H34N6O4 | [M + Na] + | 6444 | 3 | 48 FP, BSF, STR | 145457235 |
| 2.99_217.1045 | ‒ | ‒ | ‒ | ‒ | ‒ | 7530 | 4 | 48 FP | ‒ |
| 3.01_257.0986 | ‒ | ‒ | ‒ | ‒ | ‒ | 11579 | 4 | 48 FP | ‒ |
| 3.01_235.116 | ‒ | ‒ | ‒ | ‒ | ‒ | 11727 | 4 | 48 FP | ‒ |
| 3.02_332.1804 | ‒ | ‒ | ‒ | ‒ | ‒ | 12118 | 4 | 48 FP | ‒ |
| 3.03_362.2023 | ‒ | ‒ | ‒ | ‒ | ‒ | 4465 | 4 | BSF | ‒ |
| 3.07_445.2647 | N-[5-[acetyl(hydroxy)amino]pentyl]-N'-hydroxy-N'-[5-(4-oxobutanoylamino)pentyl]butanediamide | N-5-AHO | 444.5 | C20H36N4O7 | ‒ | 17803 | 3 | 48 FP, BSF, | 143733683 |
| 3.08_417.229 | ‒ | ‒ | ‒ | ‒ | ‒ | 6257 | 4 | 48 FP, BSF, STR | ‒ |
| 3.08_502.3194 | ‒ | ‒ | ‒ | ‒ | ‒ | 9297 | 4 | 48 FP, BSF, STR | ‒ |
| 3.08_447.2424 | Acetylleucylleucyllysinal hydrate | ‒ | 416.3 | C20H38N4O5 | ‒ | 11161 | 3 | BSF, STR | 44267475 |
| 3.09_500.3039 | ‒ | ‒ | ‒ | ‒ | ‒ | 39125 | 4 | 48 FP, BSF, STR | ‒ |
| 3.09_500.3096 | ‒ | ‒ | ‒ | ‒ | ‒ | 39125 | 4 | 48 FP, BSF, STR | ‒ |
| 3.09_314.17 | ‒ | ‒ | ‒ | ‒ | ‒ | 9161 | 4 | 48 FP | ‒ |
| 3.11_388.0992 | ‒ | ‒ | ‒ | ‒ | ‒ | 4459 | 4 | 48 FP | ‒ |
| 3.13_362.2276 | ‒ | ‒ | ‒ | ‒ | ‒ | 17050 | 4 | 48 FP | ‒ |
| 3.13_427.2548 | ‒ | ‒ | ‒ | ‒ | ‒ | 30969 | 4 | 48 FP | ‒ |
| 3.14_208.0578 | ‒ | ‒ | ‒ | ‒ | ‒ | 7518 | 4 | 48 FP, BSF, STR | ‒ |
| 3.15_376.2064 | ‒ | ‒ | ‒ | ‒ | ‒ | 80083 | 4 | 48 FP, BSF, STR | ‒ |
| 3.15_553.3688 | N-[5-(3-{5-[3-(5-Amino-pentylcarbamoyl)-propionylamino]-pentylcarbamoyl}-propionylamino)-pentyl]-succinamic acid | N-5-APPPPPS | 570.7 | C27H50N6O7 | [M + H - H2O] + | 4593 | 3 | 48 FP | 16654920 |
| 3.15_366.1918 | ‒ | ‒ | ‒ | ‒ | ‒ | 4746 | 4 | 48 FP, BSF, STR | ‒ |
| 3.16_398.1896 | ‒ | ‒ | ‒ | ‒ | ‒ | 6134 | 4 | 48 FP | ‒ |
| 3.16_284.1942 | ‒ | ‒ | ‒ | ‒ | ‒ | 4827 | 4 | 48 FP, BSF, STR | ‒ |
| 3.16_559.3458 | N-[5-[[4-[5-[acetyl(hydroxy)amino]pentylamino]-4-oxobutanoyl]-hydroxyamino]pentyl]-N'-(4-formamidobutyl)butanediamide | N-5-NAHAPO | 558.7 | C25H46N6O8 | ‒ | 5654 | 3 | 48 FP | 163166515 |
| 3.16_505.2386 | ‒ | ‒ | ‒ | ‒ | ‒ | 7239 | 4 | 48 FP | ‒ |
| 3.16_336.1866 | ‒ | ‒ | ‒ | ‒ | ‒ | 3530 | 4 | BSF, STR | ‒ |
| 3.16_280.2028 | ‒ | ‒ | ‒ | ‒ | ‒ | 1423 | 4 | 48 FP | ‒ |
| 3.17_264.6747 | N-(5-acetamidopentyl)-N'-[5-[[4-[5-(hydroxyamino)pentylamino]-4-oxobutanoyl]amino]pentyl]butanediamide | N-5-AHPOPB | 528.7 | C25H48N6O6 | [M + 2H] 2+ | 35741 | 3 | 48 FP | 88633559 |
| 3.18_528.3432 | ‒ | ‒ | ‒ | ‒ | ‒ | 27082 | 4 | 48 FP | ‒ |
| 3.19_550.3208 | ‒ | ‒ | ‒ | ‒ | ‒ | 2889 | 4 | 48 FP | ‒ |
| 3.2_403.2454 | N'-(5-aminopentyl)-N'-hydroxy-N-[5-[hydroxy(4-oxobutanoyl)amino]pentyl]butanediamide | N-5-AHPOPB | 402.5 | C18H34N4O6 | ‒ | 14765 | 3 | 48 FP, BSF, STR | 89072474 |
| 3.21_527.3546 | N'-[5-[[4-[5-[acetyl(hydroxy)amino]pentylamino]-4-oxobutanoyl]-hydroxyamino]pentyl]-N-(5-aminopentyl)butanediamide | N-5-AOHA | 544.7 | C25H48N6O7 | [M + H - H2O] + | 23183 | 3 | 48 FP | 86280288 |
| 3.22_403.2546 | ‒ | ‒ | ‒ | ‒ | ‒ | 14765 | 4 | 48 FP, BSF, STR | ‒ |
| 3.22_461.26 | Desferrioxamine H | Des H | 460.5 | C20H36N4O8 | ‒ | 89087 | 2 | 48 FP, BSF, STR | 57509383 |
| 3.22_461.251 | Desferrioxamine H | Des H | 460.5 | C20H36N4O8 | ‒ | 23959 | 2 | 48 FP, BSF, STR | 57509383 |
| 3.23_483.2443 | Desferrioxamine H | Des H | 460.5 | C20H36N4O8 | [M + Na] + | 8083 | 2 | 48 FP, BSF, STR | 57509383 |
| 3.23_278.1957 | Speibonoxamine | ‒ | 554.7 | C27H50N6O6 | [M + 2H] 2+ | 22794 | 3 | 48 FP | 169492462 |
| 3.23_264.18 |  |  |  |  | ‒ | 14118 | 4 | 48 FP | ‒ |
| 3.29_383.2266 | Desferrioxamine mesilate impurity B | Des mesilate impurity B | 360.5 | C16H32N4O5 | [M + Na] + | 15407 | 3 | 48 FP | 20759763 |
| 3.29_533.3294 | IC202B | ‒ | 532.6 | C23H44N6O8 | ‒ | 9622 | 3 | BSF, STR | 9828474 |
| 3.3_389.1977 | ‒ | ‒ | ‒ | ‒ | ‒ | 12888 | 4 | 48 FP, BSF, STR | ‒ |
| 3.31_286.1938 | ‒ | ‒ | ‒ | ‒ | ‒ | 10806 | 4 | 48 FP | ‒ |
| 3.31_272.6716 | N-(5-acetamidopentyl)-N'-[5-[[4-[hexyl(hydroxy)amino]-4-oxobutanoyl]amino]pentyl]-N'-hydroxybutanediamide | N-5-AHOH | 543.7 | C26H49N5O7 | [M + 2H] 2+ | 26598 | 3 | 48 FP | 134517543 |
| 3.31_411.2582 | 1,13-Dihydroxy-1,7,13,19-tetrazacyclotetracosane-2,6,14,18-tetrone | DTCTC | 428.5 | C20H36N4O6 | [M + H - H2O] + | 65714 | 3 | 48 FP | 132821026 |
| 3.32_571.3719 | ‒ | ‒ | ‒ | ‒ | ‒ | 5370 | 4 | 48 FP | ‒ |
| 3.32_569.3649 | N-(5-acetamidopentyl)-N'-[5-[[4-[5-[acetyl(hydroxy)amino]pentylamino]-4-oxobutanoyl]-hydroxyamino]pentyl]butanediamide | N-5-AAOH | 586.7 | C27H50N6O8 | ‒ | 6978 | 3 | 48 FP | 163158782 |
| 3.34_544.3365 | N-(5-acetamidopentyl)-N'-[5-[[4-[hexyl(hydroxy)amino]-4-oxobutanoyl]amino]pentyl]-N'-hydroxybutanediamide | N-5-AHOH | 543.7 | C26H49N5O7 | ‒ | 29330 | 3 | 48 FP | 134517543 |
| 3.35_315.1066 | ‒ | ‒ | ‒ | ‒ | ‒ | 5177 | 4 | 48 FP | ‒ |
| 3.36_318.1964 | ‒ | ‒ | ‒ | ‒ | ‒ | 11581 | 4 | 48 FP | ‒ |
| 3.38_582.3111 | ‒ | ‒ | ‒ | ‒ | ‒ | 7090 | 4 | 48 FP | ‒ |
| 3.38_543.3505 | Desferrioxamine B | Des B | 560.7 | C25H48N6O8 | [M +H - H2O] + | 13912 | 1 | 48 FP | 2973 |
| 3.38_435.218 | ‒ | ‒ | ‒ | ‒ | ‒ | 2521 | 4 | STR | ‒ |
| 3.39_280.6684 | N-[5-[[4-[5-[acetyl(hydroxy)amino]pentylamino]-4-oxobutanoyl]-hydroxyamino]pentyl]-N'-(4-formamidobutyl)butanediamide | N-5-NAHAPO | 558.7 | C25H46N6O8 | [M + 2H] 2+ | 63193 | 3 | 48 FP, BSF | 163166515 |
| 3.39_295.1806 | Desferrioxamine D3 | Des D3 | 588.7 | C26H48N6O9 | [M + 2H] 2+ | 2709 | 3 | 48 FP, BSF | 154585487 |
| 3.39_587.3218 | ‒ | ‒ | ‒ | ‒ | ‒ | 2822 | 4 | BSF, STR | ‒ |
| 3.4_530.3208 | ‒ | ‒ | ‒ | ‒ | ‒ | 6734 | 4 | 48 FP | ‒ |
| 3.41_560.33 | N-[5-[[4-[5-[acetyl(hydroxy)amino]pentylamino]-4-oxobutanoyl]-hydroxyamino]pentyl]-N'-(4-formamidobutyl)butanediamide | N-5-NAHAPO | 558.7 | C25H46N6O8 | ‒ | 87919 | 3 | 48 FP, BSF | 163166515 |
| 3.42_560.3199 | N-[5-[[4-[5-[acetyl(hydroxy)amino]pentylamino]-4-oxobutanoyl]-hydroxyamino]pentyl]-N'-(4-formamidobutyl)butanediamide | N-5-NAHAPO | 558.7 | C25H46N6O8 | ‒ | 54348 | 3 | 48 FP, BSF | 163166515 |
| 3.42_609.3035 | ‒ | ‒ | ‒ | ‒ | ‒ | 1331 | 4 | BSF | ‒ |
| 3.44_587.3403 | Desferrioxamine D2 | Des D2 | 586.7 | C26H46N6O9 | ‒ | 9965 | 2 | 48 FP, BSF, STR | 14671620 |
| 3.44_294.1835 | ‒ | ‒ | ‒ | ‒ | ‒ | 12229 | 4 | 48 FP, BSF, STR | ‒ |
| 3.45_427.2533 | N-[5-[acetyl(hydroxy)amino]pentyl]-N'-hydroxy-N'-[5-(4-oxobutanoylamino)pentyl]butanediamide | N-5-AHO | 444.5 | C20H36N4O7 | [M +H - H2O] + | 49227 | 3 | 48 FP, BSF, STR | 143733683 |
| 3.45_390.1025 | ‒ | ‒ | ‒ | ‒ | ‒ | 12142 | 4 | 48 FP, BSF, STR | ‒ |
| 3.45_323.1699 | ‒ | ‒ | ‒ | ‒ | ‒ | 23429 | 4 | 48 FP | ‒ |
| 3.45_345.1515 | ‒ | ‒ | ‒ | ‒ | ‒ | 18115 | 4 | 48 FP | ‒ |
| 3.46_532.2989 | N-[5-[[4-[5-[acetyl(hydroxy)amino]pentylamino]-4-oxobutanoyl]-hydroxyamino]pentyl]-N'-butyl-N'-hydroxybutanediamide | N-5-AOHBH | 531.6 | C24H45N5O8 | ‒ | 18805 | 3 | 48 FP | 165618941 |
| 3.46_585.3603 | Dehydroxynocardamine | Dehydroxy-Des E | 584.7 | C27H48N6O8 | ‒ | 46113 | 2 | 48 FP, BSF | 11606728 |
| 3.46_293.184 | ‒ | ‒ | ‒ | ‒ | ‒ | 30213 | 4 | 48 FP, BSF | ‒ |
| 3.46_449.1973 | ‒ | ‒ | ‒ | ‒ | ‒ | 3787 | 4 | STR | ‒ |
| 3.47_532.2888 | ‒ | ‒ | ‒ | ‒ | ‒ | 18320 | 4 | 48 FP | ‒ |
| 3.48_585.35 | Dehydroxynocardamine | Dehydroxy-Des E | 584.7 | C27H48N6O8 | ‒ | 30220 | 2 | 48 FP, BSF | 11606728 |
| 3.49_546.3071 | N-[5-[[4-[5-[acetyl(hydroxy)amino]pentylamino]-4-oxobutanoyl]-hydroxyamino]pentyl]-N'-hydroxy-N'-pentylbutanediamide | N-5-AOHHP | 545.7 | C25H47N5O8 | ‒ | 16581 | 3 | 48 FP, BSF | 137444661 |
| 3.51_243.1311 | ‒ | ‒ | ‒ | ‒ | ‒ | 71122 | 4 | 48 FP, BSF, STR | ‒ |
| 3.51_399.2209 | ‒ | ‒ | ‒ | ‒ | ‒ | 9877 | 4 | 48 FP, BSF, STR | ‒ |
| 3.51_503.3058 | ‒ | ‒ | ‒ | ‒ | ‒ | 12262 | 4 | 48 FP, BSF, STR | ‒ |
| 3.52_288.6655 | ‒ | ‒ | ‒ | ‒ | ‒ | 180552 | 4 | 48 FP, BSF, STR | ‒ |
| 3.52_361.2427 | L-lysyl-L-threonyl-L-isoleucine | ‒ | 360.45 | C16H32N4O5 | ‒ | 22296 | 3 | 48 FP, BSF, STR | 145456782 |
| 3.52_443.2497 | Desferrioxamine H | Des H | 460.5 | C20H36N4O8 | [M +H - H2O] + | 88071 | 2 | 48 FP, BSF, STR | 57509383 |
| 3.52_361.2382 | ‒ | ‒ | ‒ | ‒ | ‒ | 22296 | 4 | 48 FP, BSF, STR | ‒ |
| 3.53_625.3441 | ‒ | ‒ | ‒ | ‒ | ‒ | 10100 | 4 | 48 FP, BSF, STR | ‒ |
| 3.53_576.3263 | 5-[[4-[5-[[4-[5-[Acetyl(hydroxy)amino]pentylamino]-4-oxobutanoyl]-hydroxyamino]pentylamino]-4-oxobutanoyl]-hydroxyamino]pentanoic acid | N-5-AOHOH | 575.7 | C25H45N5O10 | ‒ | 281908 | 2 | 48 FP, BSF, STR | 14459425 |
| 3.53_359.1312 | ‒ | ‒ | ‒ | ‒ | ‒ | 16282 | 4 | 48 FP | ‒ |
| 3.53_337.1504 | ‒ | ‒ | ‒ | ‒ | ‒ | 10225 | 4 | 48 FP | ‒ |
| 3.53_213.1547 | ‒ | ‒ | ‒ | ‒ | ‒ | 4119 | 4 | 48 FP, BSF, STR | ‒ |
| 3.53_520.3313 | ‒ | ‒ | ‒ | ‒ | ‒ | 7844 | 4 | 48 FP STR | ‒ |
| 3.54_443.2476 | Desferrioxamine H | Des H | 460.5 | C20H36N4O8 | [M +H - H2O] + | 88071 | 2 | 48 FP, BSF, STR | 57509383 |
| 3.54_576.3174 | 5-[[4-[5-[[4-[5-[Acetyl(hydroxy)amino]pentylamino]-4-oxobutanoyl]-hydroxyamino]pentylamino]-4-oxobutanoyl]-hydroxyamino]pentanoic acid | N-5-AOHOH | 575.7 | C25H45N5O10 | ‒ | 235894 | 2 | 48 FP, BSF, STR | 14459425 |
| 3.54_334.1958 | ‒ | ‒ | ‒ | ‒ | ‒ | 54046 | 4 | 48 FP, BSF, STR | ‒ |
| 3.54_217.105 | ‒ | ‒ | ‒ | ‒ | ‒ | 5644 | 4 | 48 FP | ‒ |
| 3.6_623.331 | ‒ | ‒ | ‒ | ‒ | ‒ | 13752 | 4 | 48 FP, BSF, STR | ‒ |
| 3.6_301.1801 | Desferrioxamine E | Des E | 600.7 | C27H48N6O9 | [M + 2H] 2+ | 61343 | 1 | 48 FP, BSF, STR | 161532 |
| 3.6_601.3561 | Desferrioxamine E | Des E | 600.7 | C27H48N6O9 | ‒ | 113281 | 1 | 48 FP, BSF, STR | 161532 |
| 3.6_623.3372 | Desferrioxamine E | Des E | 600.7 | C27H48N6O9 | [M + Na] + | 13752 | 1 | 48 FP, BSF, STR | 161532 |
| 3.6_264.1782 | ‒ | ‒ | ‒ | ‒ | ‒ | 16234 | 4 | STR | ‒ |
| 3.6_564.3576 | ‒ | ‒ | ‒ | ‒ | ‒ | 21467 | 2 | 48 FP, BSF, STR | ‒ |
| 3.61_280.6672 | N-[5-[[4-[5-[acetyl(hydroxy)amino]pentylamino]-4-oxobutanoyl]-hydroxyamino]pentyl]-N'-(4-formamidobutyl)butanediamide | N-5-NAHAPO | 558.7 | C25H46N6O8 | [M + 2H] 2+ | 10357 | 2 | 48 FP, BSF, | 163166515 |
| 3.61_547.3327 | N-[5-[[4-[4-[acetyl(hydroxy)amino]butylamino]-4-oxobutanoyl]-hydroxyamino]pentyl]-N'-(5-aminopentyl)-N'-hydroxybutanediamide | N-5-AOHAH | 546.7 | C24H46N6O8 | ‒ | 29915 | 2 | 48 FP, BSF, STR | 14029344 |
| 3.61_286.1608 | ‒ | ‒ | ‒ | ‒ | ‒ | 10301 | 4 | STR | ‒ |
| 3.63_560.3332 | ‒ | ‒ | ‒ | ‒ | ‒ | 34993 | 4 | 48 FP, BSF, STR | ‒ |
| 3.67_318.1735 | ‒ | ‒ | ‒ | ‒ | ‒ | 20591 | 4 | 48 FP, BSF, STR | ‒ |
| 3.68_582.3094 | ‒ | ‒ | ‒ | ‒ | ‒ | 6027 | 4 | 48 FP | ‒ |
| 3.68_459.2727 | ‒ | ‒ | ‒ | ‒ | ‒ | 2003 | 4 | BSF | ‒ |
| 3.69_591.3611 | ‒ | ‒ | ‒ | ‒ | ‒ | 13052 | 4 | 48 FP, BSF, STR | ‒ |
| 3.69_608.3868 | ‒ | ‒ | ‒ | ‒ | ‒ | 9744 | 4 | BSF, STR | ‒ |
| 3.73_269.0565 | ‒ | ‒ | ‒ | ‒ | ‒ | 14404 | 4 | 48 FP, BSF | ‒ |
| 3.75_313.0467 | ‒ | ‒ | ‒ | ‒ | ‒ | 8188 | 4 | 48 FP | ‒ |
| 3.75_473.2891 | ‒ | ‒ | ‒ | ‒ | ‒ | 2078 | 4 | BSF | ‒ |
| 3.76_574.3092 | N-[5-[[4-[5-[acetyl(hydroxy)amino]pentylamino]-4-oxobutanoyl]-hydroxyamino]pentyl]-N'-heptyl-N'-hydroxybutanediamide | N-5-AOHH | 573.7 | C27H51N5O8 | ‒ | 6004 | 3 | 48 FP | 14078495 |
| 3.77_419.139 | ‒ | ‒ | ‒ | ‒ | ‒ | 6595 | 4 | 48 FP | ‒ |
| 3.77_441.2943 | Leupeptin Pr | ‒ | 440.6 | C21H40N6O4 | ‒ | 2645 | 3 | BSF | 13209091 |
| 3.78_599.33 | N-Hydroxy-desferrioxamine B | Hydroxy-Des B | 576.7 | C25H48N6O9 | [M + Na] + | 3819 | 3 | 48 FP, BSF | 44149952 |
| 3.79_599.3393 | N-Hydroxy-desferrioxamine B | Hydroxy-Des B | 576.7 | C25H48N6O9 | [M + Na] + | 8394 | 3 | 48 FP, BSF | 44149952 |
| 3.82_378.2319 | ‒ | ‒ | ‒ | ‒ | ‒ | 6324 | 4 | BSF, STR | ‒ |
| 3.82_358.2617 | ‒ | ‒ | ‒ | ‒ | ‒ | 3836 | 4 | BSF, STR | ‒ |
| 3.86_362.1781 | ‒ | ‒ | ‒ | ‒ | ‒ | 5246 | 4 | 48 FP | ‒ |
| 3.92_434.1993 | ‒ | ‒ | ‒ | ‒ | ‒ | 2857 | 4 | 48 FP | ‒ |
| 3.92_507.239 | ‒ | ‒ | ‒ | ‒ | ‒ | 4527 | 4 | STR | ‒ |
| 3.95_475.2781 | ‒ | ‒ | ‒ | ‒ | ‒ | 1863 | 4 | BSF | ‒ |
| 4_384.2355 | ‒ | ‒ | ‒ | ‒ | ‒ | 5848 | 4 | STR | ‒ |
| 4.04_219.0556 | ‒ | ‒ | ‒ | ‒ | ‒ | 5028 | 4 | 48 FP | ‒ |
| 4.07_331.1708 | ‒ | ‒ | ‒ | ‒ | ‒ | 3187 | 4 | STR | ‒ |
| 4.08_457.2982 | ‒ | ‒ | ‒ | ‒ | ‒ | 2948 | 4 | STR | ‒ |
| 4.08_309.1909 | ‒ | ‒ | ‒ | ‒ | ‒ | 2414 | 4 | STR | ‒ |
| 4.1_164.1011 | ‒ | ‒ | ‒ | ‒ | ‒ | 3678 | 4 | 48 FP, STR | ‒ |
| 4.16_288.1138 | N-[5-[acetyl(hydroxy)amino]pentyl]-N'-hydroxy-N'-[5-[[4-[hydroxy-[(5E)-5-hydroxyiminopentyl]amino]-4-oxobutanoyl]amino]pentyl]butanediamide | N-5-AHHHO | 574.7 | C25H46N6O9 | [M + 2H] 2+ | 4435 | 3 | 48 FP | 10393334 |
| 4.21_322.2201 | ‒ | ‒ | ‒ | ‒ | ‒ | 11404 | 4 | STR | ‒ |
| 4.22_164.1058 | ‒ | ‒ | ‒ | ‒ | ‒ | 7980 | 4 | 48 FP STR | ‒ |
| 4.22_388.1572 | ‒ | ‒ | ‒ | ‒ | ‒ | 10117 | 4 | 48 FP, BSF, STR | ‒ |
| 4.22_400.2874 | ‒ | ‒ | ‒ | ‒ | ‒ | 5273 | 4 | STR | ‒ |
| 4.22_331.1699 | ‒ | ‒ | ‒ | ‒ | ‒ | 2168 | 4 | STR | ‒ |
| 4.23_388.1493 | ‒ | ‒ | ‒ | ‒ | ‒ | 10117 | 4 | 48 FP, BSF, STR | ‒ |
| 4.25_318.1257 | ‒ | ‒ | ‒ | ‒ | ‒ | 8361 | 4 | 48 FP | ‒ |
| 4.27_413.1187 | ‒ | ‒ | ‒ | ‒ | ‒ | 11781 | 4 | 48 FP | ‒ |
| 4.3_197.0946 | ‒ | ‒ | ‒ | ‒ | ‒ | 8948 | 4 | 48 FP | ‒ |
| 4.3_355.1174 | ‒ | ‒ | ‒ | ‒ | ‒ | 11636 | 4 | 48 FP | ‒ |
| 4.35_565.2806 | ‒ | ‒ | ‒ | ‒ | ‒ | 2368 | 4 | STR | ‒ |
| 4.37_385.1466 | ‒ | ‒ | ‒ | ‒ | ‒ | 33309 | 4 | 48 FP | ‒ |
| 4.37_363.1645 | ‒ | ‒ | ‒ | ‒ | ‒ | 25008 | 4 | 48 FP | ‒ |
| 4.37_345.1541 | ‒ | ‒ | ‒ | ‒ | ‒ | 18413 | 4 | 48 FP | ‒ |
| 4.45_302.1298 | Desferrioxamine Et1 | Des Et1 | 602.7 | C26H46N6O10 | [M + 2H] 2+ | 34179 | 2 | 48 FP | 14671623 |
| 4.46_324.112 | ‒ | ‒ | ‒ | ‒ | ‒ | 5506 | 4 | 48 FP | ‒ |
| 4.48_213.0896 | ‒ | ‒ | ‒ | ‒ | ‒ | 10273 | 4 | 48 FP | ‒ |
| 4.52_451.2182 | N-benzoyl-L-leucyl-L-alpha-aspartyl-L-threoninamide | ‒ | 450.5 | C21H30N4O7 | ‒ | 55195 | 3 | 48 FP | 44287994 |
| 4.53_473.2006 | ‒ | ‒ | ‒ | ‒ | ‒ | 48768 | 4 | 48 FP | ‒ |
| 4.53_468.2443 | ‒ | ‒ | ‒ | ‒ | ‒ | 19109 | 4 | 48 FP | ‒ |
| 4.53_411.1031 | ‒ | ‒ | ‒ | ‒ | ‒ | 14672 | 4 | 48 FP | ‒ |
| 4.59_213.0899 | ‒ | ‒ | ‒ | ‒ | ‒ | 10273 | 4 | 48 FP | ‒ |
| 4.6_411.105 | ‒ | ‒ | ‒ | ‒ | ‒ | 14672 | 4 | 48 FP | ‒ |
| 4.61_414.3037 | ‒ | ‒ | ‒ | ‒ | ‒ | 5830 | 4 | STR | ‒ |
| 4.65_300.1142 | ‒ | ‒ | ‒ | ‒ | ‒ | 17043 | 4 | 48 FP | ‒ |
| 4.67_458.3303 | ‒ | ‒ | ‒ | ‒ | ‒ | 3149 | 4 | STR | ‒ |
| 4.68_463.284 | ‒ | ‒ | ‒ | ‒ | ‒ | 4613 | 4 | STR | ‒ |
| 4.69_380.2613 | ‒ | ‒ | ‒ | ‒ | ‒ | 2391 | 4 | STR | ‒ |
| 4.74_297.0512 | ‒ | ‒ | ‒ | ‒ | ‒ | 176149 | 4 | 48 FP, BSF, STR | ‒ |
| 4.74_640.2306 | ‒ | ‒ | ‒ | ‒ | ‒ | 8748 | 4 | 48 FP | ‒ |
| 4.75_364.0934 | ‒ | ‒ | ‒ | ‒ | ‒ | 103801 | 4 | 48 FP, BSF | ‒ |
| 4.75_279.0401 | ‒ | ‒ | ‒ | ‒ | ‒ | 16831 | 4 | 48 FP, BSF | ‒ |
| 4.76_485.3294 | ‒ | ‒ | ‒ | ‒ | ‒ | 4952 | 4 | STR | ‒ |
| 4.76_507.3106 | ‒ | ‒ | ‒ | ‒ | ‒ | 2178 | 4 | STR | ‒ |
| 4.84_529.3564 | ‒ | ‒ | ‒ | ‒ | ‒ | 3661 | 4 | STR | ‒ |
| 4.9_453.2579 | ‒ | ‒ | ‒ | ‒ | ‒ | 3053 | 4 | 48 FP | ‒ |
| 4.9_431.2745 | ‒ | ‒ | ‒ | ‒ | ‒ | 3842 | 4 | 48 FP | ‒ |
| 5.04_497.2842 | ‒ | ‒ | ‒ | ‒ | ‒ | 10605 | 4 | 48 FP | ‒ |
| 5.05_475.3018 | ‒ | ‒ | ‒ | ‒ | ‒ | 12704 | 4 | 48 FP | ‒ |
| 5.14_401.213 | ‒ | ‒ | ‒ | ‒ | ‒ | 2739 | 4 | STR | ‒ |
| 5.21_237.088 | ‒ | ‒ | ‒ | ‒ | ‒ | 32397 | 4 | 48 FP | ‒ |
| 5.21_399.1613 | ‒ | ‒ | ‒ | ‒ | ‒ | 7816 | 4 | 48 FP | ‒ |
| 5.21_543.3746 | ‒ | ‒ | ‒ | ‒ | ‒ | 6609 | 4 | STR | ‒ |
| 5.35_464.1978 | ‒ | ‒ | ‒ | ‒ | ‒ | 26218 | 4 | 48 FP | ‒ |
| 5.43_601.2473 | ‒ | ‒ | ‒ | ‒ | ‒ | 18360 | 4 | 48 FP | ‒ |
| 5.44_579.2668 | ‒ | ‒ | ‒ | ‒ | ‒ | 12451 | 4 | 48 FP | ‒ |
| 5.45_541.1643 | ‒ | ‒ | ‒ | ‒ | ‒ | 48893 | 4 | 48 FP | ‒ |
| 5.45_563.1454 | ‒ | ‒ | ‒ | ‒ | ‒ | 5355 | 4 | 48 FP | ‒ |
| 5.47_339.1076 | ‒ | ‒ | ‒ | ‒ | ‒ | 3890 | 4 | 48 FP | ‒ |
| 5.5_689.2997 | ‒ | ‒ | ‒ | ‒ | ‒ | 16543 | 4 | 48 FP | ‒ |
| 5.5_667.3142 | ‒ | ‒ | ‒ | ‒ | ‒ | 11720 | 4 | 48 FP | ‒ |
| 5.51_311.0665 | ‒ | ‒ | ‒ | ‒ | ‒ | 4448 | 4 | 48 FP, BSF | ‒ |
| 5.57_478.1783 | ‒ | ‒ | ‒ | ‒ | ‒ | 24030 | 4 | 48 FP | ‒ |
| 5.57_555.1439 | ‒ | ‒ | ‒ | ‒ | ‒ | 2895 | 4 | 48 FP | ‒ |
| 5.71_413.1782 | L-asparagyl-L-asparagyl-L-prolyl-L-serine | ‒ | 430.41 | C16H26N6O8 | [M + H - H2O] + | 8450 | 3 | 48 FP | 71464612 |
| 5.71_391.195 | ‒ | ‒ | ‒ | ‒ | ‒ | 2466 | 4 | 48 FP | ‒ |
| 5.75_393.1595 | ‒ | ‒ | ‒ | ‒ | ‒ | 4049 | 4 | 48 FP | ‒ |
| 5.79_625.3294 | L-alanyl-L-prolyl-glycyl-L-prolyl-L-seryl-L-lysyl-L-serine | ‒ | 642.7 | C27H46N8O10 | ‒ | 2646 | 3 | 48 FP | 10233147 |
| 5.8_603.3486 | Desferrioxamine Et1 | Des Et1 | 602.7 | C26H46N6O10 | ‒ | 2720 | 2 | 48 FP | 14671623 |
| 5.87_378.1249 | L-alpha-aspartyl-L-alpha-aspartyl-L-phenylalanine | ‒ | 395.4 | C17H21N3O8 | [M + H - H2O] + | 17206 | 3 | 48 FP | 471581 |
| 6.11_817.3465 | ‒ | ‒ | ‒ | ‒ | ‒ | 6558 | 4 | 48 FP | ‒ |
| 6.19_463.3257 | ‒ | ‒ | ‒ | ‒ | ‒ | 4495 | 4 | STR | ‒ |
| 6.21_274.2716 | ‒ | ‒ | ‒ | ‒ | ‒ | 1729 | 4 | STR | ‒ |
| 6.29_532.2869 | ‒ | ‒ | ‒ | ‒ | ‒ | 125308 | 4 | 48 FP, BSF, STR | ‒ |
| 6.29_532.2925 | ‒ | ‒ | ‒ | ‒ | ‒ | 99985 | 4 | 48 FP, BSF, STR | ‒ |
| 6.29_514.2803 | ‒ | ‒ | ‒ | ‒ | ‒ | 3025 | 4 | 48 FP | ‒ |
| 6.3_572.2844 | DL-tyrosyl-DL-isoleucyl-DL-asparagyl-DL-tyrosine | ‒ | 571.6 | C28H37N5O8 | ‒ | 10440 | 3 | 48 FP, BSF, STR | 19953641 |
| 6.31_572.2744 | ‒ | ‒ | ‒ | ‒ | ‒ | 9173 | 4 | 48 FP, BSF, STR | ‒ |
| 6.35_365.1293 | ‒ | ‒ | ‒ | ‒ | ‒ | 5264 | 4 | 48 FP | ‒ |
| 6.36_387.1107 | ‒ | ‒ | ‒ | ‒ | ‒ | 3854 | 4 | 48 FP | ‒ |
| 6.64_499.7558 | ‒ | ‒ | ‒ | ‒ | ‒ | 1144 | 4 | BSF | ‒ |
| 6.67_552.3162 | ‒ | ‒ | ‒ | ‒ | ‒ | 3931 | 4 | 48 FP STR | ‒ |
| 6.72_499.7772 | ‒ | ‒ | ‒ | ‒ | ‒ | 63598 | 4 | 48 FP, BSF, STR | ‒ |
| 6.72_490.77 | ‒ | ‒ | ‒ | ‒ | ‒ | 31429 | 4 | 48 FP, BSF, STR | ‒ |
| 6.72_521.3672 | ‒ | ‒ | ‒ | ‒ | ‒ | 5376 | 4 | STR | ‒ |
| 6.86_687.4844 | ‒ | ‒ | ‒ | ‒ | ‒ | 11534 | 4 | STR | ‒ |
| 6.96_448.2022 | L-valyl-L-seryl-L-seryl-L-seryl-L-serine | ‒ | 465.5 | C17H31N5O10 | [M + H - H2O] + | 6653 | 3 | 48 FP | 134824945 |
| 6.98_326.994 | ‒ | ‒ | ‒ | ‒ | ‒ | 2055 | 4 | 48 FP, BSF | ‒ |
| 6.98_348.9742 | ‒ | ‒ | ‒ | ‒ | ‒ | 1337 | 4 | BSF | ‒ |
| 6.98_350.9705 | ‒ | ‒ | ‒ | ‒ | ‒ | 1403 | 4 | BSF | ‒ |
| 7.19_579.4077 | ‒ | ‒ | ‒ | ‒ | ‒ | 5058 | 4 | STR | ‒ |
| 7.26_281.1151 | ‒ | ‒ | ‒ | ‒ | ‒ | 10523 | 4 | 48 FP | ‒ |
| 7.26_439.3056 | ‒ | ‒ | ‒ | ‒ | ‒ | 5544 | 4 | STR | ‒ |
| 7.27_745.5287 | ‒ | ‒ | ‒ | ‒ | ‒ | 5578 | 4 | STR | ‒ |
| 7.27_395.2804 | ‒ | ‒ | ‒ | ‒ | ‒ | 5496 | 4 | STR | ‒ |
| 7.28_417.2916 | ‒ | ‒ | ‒ | ‒ | ‒ | 5520 | 4 | STR | ‒ |
| 7.3_597.4186 | ‒ | ‒ | ‒ | ‒ | ‒ | 6474 | 4 | STR | ‒ |
| 7.56_535.2106 | ‒ | ‒ | ‒ | ‒ | ‒ | 48389 | 4 | 48 FP | ‒ |
| 7.56_225.0902 | ‒ | ‒ | ‒ | ‒ | ‒ | 9755 | 4 | 48 FP | ‒ |
| 7.57_279.0979 | ‒ | ‒ | ‒ | ‒ | ‒ | 146203 | 4 | 48 FP | ‒ |
| 7.66_468.326 | ‒ | ‒ | ‒ | ‒ | ‒ | 6723 | 4 | STR | ‒ |
| 7.67_637.4472 | ‒ | ‒ | ‒ | ‒ | ‒ | 4413 | 4 | STR | ‒ |
| 7.7_424.2992 | ‒ | ‒ | ‒ | ‒ | ‒ | 12125 | 4 | STR | ‒ |
| 7.7_446.3121 | ‒ | ‒ | ‒ | ‒ | ‒ | 8376 | 4 | STR | ‒ |
| 7.73_402.2857 | ‒ | ‒ | ‒ | ‒ | ‒ | 11890 | 4 | STR | ‒ |
| 7.75_655.4587 | ‒ | ‒ | ‒ | ‒ | ‒ | 6518 | 4 | STR | ‒ |
| 8.07_497.343 | ‒ | ‒ | ‒ | ‒ | ‒ | 7267 | 4 | STR | ‒ |
| 8.1_453.3183 | ‒ | ‒ | ‒ | ‒ | ‒ | 17272 | 4 | STR | ‒ |
| 8.1_475.3304 | ‒ | ‒ | ‒ | ‒ | ‒ | 12799 | 4 | STR | ‒ |
| 8.12_431.3068 | ‒ | ‒ | ‒ | ‒ | ‒ | 15022 | 4 | STR | ‒ |
| 8.12_695.4884 | ‒ | ‒ | ‒ | ‒ | ‒ | 4197 | 4 | STR | ‒ |
| 8.16_371.1199 | ‒ | ‒ | ‒ | ‒ | ‒ | 5616 | 4 | 48 FP | ‒ |
| 8.21_787.5383 | ‒ | ‒ | ‒ | ‒ | ‒ | 4588 | 4 | STR | ‒ |
| 8.26_409.293 | ‒ | ‒ | ‒ | ‒ | ‒ | 19135 | 4 | STR | ‒ |
| 8.59_471.3214 | ‒ | ‒ | ‒ | ‒ | ‒ | 12503 | 4 | STR | ‒ |
| 8.67_449.3073 | ‒ | ‒ | ‒ | ‒ | ‒ | 13946 | 4 | STR | ‒ |
| 8.76_438.2902 | ‒ | ‒ | ‒ | ‒ | ‒ | 36722 | 4 | STR | ‒ |
| 8.88_760.5375 | ‒ | ‒ | ‒ | ‒ | ‒ | 2946 | 4 | STR | ‒ |
| 8.89_522.3558 | ‒ | ‒ | ‒ | ‒ | ‒ | 9762 | 4 | STR | ‒ |
| 8.9_445.3891 | ‒ | ‒ | ‒ | ‒ | ‒ | 4354 | 4 | 48 FP STR | ‒ |
| 8.97_511.3446 | ‒ | ‒ | ‒ | ‒ | ‒ | 15188 | 4 | STR | ‒ |
| 9.12_301.128 | ‒ | ‒ | ‒ | ‒ | ‒ | 4030 | 4 | 48 FP, BSF, STR | ‒ |
| 10.07_546.4839 | ‒ | ‒ | ‒ | ‒ | ‒ | 2574 | 4 | 48 FP, BSF, STR | ‒ |
| 10.18_546.4782 | ‒ | ‒ | ‒ | ‒ | ‒ | 3814 | 4 | 48 FP, BSF, STR | ‒ |
| 10.4_311.254 | ‒ | ‒ | ‒ | ‒ | ‒ | 1526 | 4 | 48 FP STR | ‒ |
| 11.77_659.963 | ‒ | ‒ | ‒ | ‒ | ‒ | 8305 | 4 | STR | ‒ |
| 12.13_688.4836 | ‒ | ‒ | ‒ | ‒ | ‒ | 1747 | 4 | STR | ‒ |
| 12.19_662.4679 | ‒ | ‒ | ‒ | ‒ | ‒ | 1632 | 4 | STR | ‒ |
| 12.43_338.3263 | ‒ | ‒ | ‒ | ‒ | ‒ | 2275 | 4 | BSF | ‒ |

^†^*48 flower plates = 48 FP, Baffles shake flask = BSF, Stirred tank bioreactor = STR.*

**Table S6:** List of all identified non-ribosomal peptides across all cultivation scales. The list comprises the peptide name, neutral mass, sum formula, detected adducts, chemical structure, annotation confidence level and the cultivation systems in which the non-ribosomal peptides were detected.

| **Name** | **Neutral mass [g/mol]** | **Sum formula** | **Adduct** | **Structure** | **Tier** | **48 FP** | **BSF** | **STR** |
| --- | --- | --- | --- | --- | --- | --- | --- | --- |
| L-alanyl-L-prolyl-glycyl-L-prolyl-L-seryl-L-lysyl-L-serine | 642.7 | C_27_H_46_N_8_O_10_ | [M + H] + |  | 3 | ✓ |  |  |
| DL-tyrosyl-DL-isoleucyl-DL-asparagyl-DL-tyrosine | 571.6 | C_28_H_37_N_5_O_8_ | [M + H] + |  | 3 | ✓ | ✓ | ✓ |
| L-leucyl-L-prolyl-L-glutaminyl-L-alanyl-glycine | 484.5 | C_21_H_36_N_6_O_7_ | [M + H - H_2_O] + |  | 3 | ✓ | ✓ | ✓ |
| L-valyl-L-seryl-L-seryl-L-seryl-L-serine | 465.5 | C_17_H_31_N_5_O_10_ | [M + H - H_2_O] + |  | 3 | ✓ |  |  |
| N-benzoyl-L-leucyl-L-alpha-aspartyl-L-threoninamide | 450.5 | C_21_H_30_N_4_O_7_ | [M + H] + |  | 3 | ✓ |  |  |
| Leupeptin Pr | 440.6 | C_21_H_40_N_6_O_4_ | [M + H] + |  | 3 |  | ✓ |  |
| L-phenylalanyl-L-isoleucyl-L-arginine | 434.5 | C_21_H_34_N_6_O_4_ | [M + Na] + |  | 3 | ✓ | ✓ | ✓ |
| L-phenylalanyl-L-leucyl-L-histidine hydroxyamide | 430.5 | C_21_H_30_N_6_O_4_ | [M + H] + |  | 3 |  | ✓ | ✓ |
| L-asparagyl-L-asparagyl-L-prolyl-L-serine | 430.41 | C_16_H_26_N_6_O_8_ | [M + H - H_2_O] + |  | 3 | ✓ |  |  |
| Acetylleucylleucyllysinal hydrate | 416.3 | C_20_H_38_N_4_O_5_ | [M + H] + |  | 3 |  | ✓ | ✓ |
| L-alpha-aspartyl-L-alpha-aspartyl-L-phenylalanine | 395.4 | C_17_H_21_N_3_O_8_ | [M + H - H_2_O] + |  | 3 | ✓ |  |  |
| L-lysyl-L-threonyl-L-isoleucine | 360.45 | C_16_H_32_N_4_O_5_ | [M + H] + |  | 3 | ✓ | ✓ | ✓ |
| L-leucyl-L-threonyl-L-threonine | 333.38 | C_14_H_27_N_3_O_6_ | [M + H - H_2_O] + |  | 3 | ✓ | ✓ |  |

**Table S7:** List of all identified desferrioxamine chemicals across all cultivation scales. The list comprises the name, neutral mass, sum formula, detected adducts, chemical structure, annotation confidence level and the cultivation systems in which the siderophore were detected.

| **Name**^†^ | **Neutral mass [g/mol]** | **Sum formula** | **Adduct** | **Structure** | **Tier** | **48 FP** | **BSF** | **STR** |
| --- | --- | --- | --- | --- | --- | --- | --- | --- |
| Des Et1 | 602.7 | C_26_H_46_N_6_O_10_ | [M + H] +, [M+ 2H] 2+ |  | 2 | ✓ |  |  |
| Des E | 600.7 | C_27_H_48_N_6_O_9_ | [M + H] +, [M + Na] +,  [M+ 2H] 2+ |  | 1 | ✓ | ✓ | ✓ |
| Des D3 | 588.7 | C_26_H_48_N_6_O_9_ | [M+ 2H] 2+ |  | 3 | ✓ | ✓ |  |
| Des D2 | 586.7 | C_26_H_46_N_6_O_9_ | [M + H] + |  | 2 | ✓ | ✓ | ✓ |
| N-5-AAOH | 586.7 | C_27_H_50_N_6_O_8_ | [M + H] + |  | 3 | ✓ |  |  |
| Dehydroxy-Des E | 584.7 | C_27_H_48_N_6_O_8_ | [M + H] + |  | 2 | ✓ | ✓ |  |
| Hydroxy-Des B | 576.7 | C_25_H_48_N_6_O_9_ | [M + H] +, [M + Na] +,  [M+ 2H] 2+ |  | 3 | ✓ | ✓ | ✓ |
| N-5-AOHOH | 575.7 | C_25_H_45_N_5_O_10_ | [M + H] + |  | 2 | ✓ | ✓ | ✓ |
| N-5-AHHHO | 574.7 | C_25_H_46_N_6_O_9_ | [M+ 2H] 2+ |  | 3 | ✓ |  |  |
| N-5-AOHH | 573.7 | C_27_H_51_N_5_O_8_ | [M + H] + |  | 3 | ✓ |  |  |
| N-5-APPPPPS | 570.7 | C_27_H_50_N_6_O_7_ | [M + H - H_2_O] + |  | 3 | ✓ |  |  |
| Des B | 560.7 | C_25_H_48_N_6_O_8_ | [M + H] +, [M + H - H_2_O] +,  [M+ 2H] 2+ |  | 1 | ✓ | ✓ | ✓ |
| N-5-NAHAPO | 558.7 | C_25_H_46_N_6_O_8_ | [M + H] +, [M+ 2H] 2+ |  | 2 | ✓ | ✓ |  |
| Speibonoxamine | 554.7 | C_27_H_50_N_6_O_6_ | [M+ 2H] 2+ |  | 3 | ✓ |  |  |
| N-5-AOHAH | 546.7 | C_24_H_46_N_6_O_8_ | [M + H] + |  | 2 | ✓ | ✓ | ✓ |
| N-5-AOHHP | 545.7 | C_25_H_47_N_5_O_8_ | [M + H] +, [M+ 2H] 2+ |  | 3 | ✓ | ✓ | ✓ |
| N-5-AAO | 544.7 | C_25_H_48_N_6_O_7_ | [M + H] + |  | 3 | ✓ | ✓ | ✓ |
| N-5-AOHA | 544.7 | C_25_H_48_N_6_O_7_ | [M + H - H_2_O] + |  | 3 | ✓ |  |  |
| N-5-AHOH | 543.7 | C_26_H_49_N_5_O_7_ | [M + H] +, [M+ 2H] 2+ |  | 3 | ✓ |  |  |
| N-5-ADOHH | 534.6 | C_23_H_46_N_6_O_8_ | [M + H - H_2_O] + |  | 3 | ✓ | ✓ | ✓ |
| IC202B | 532.6 | C_23_H_44_N_6_O_8_ | [M + H] + |  | 3 |  | ✓ | ✓ |
| N-5-AOHBH | 531.6 | C_24_H_45_N_5_O_8_ | [M + H] + |  | 3 | ✓ |  |  |
| N-5-AHPOPB | 528.7 | C_25_H_48_N_6_O_6_ | [M + H] +, [M+ 2H] 2+ |  | 3 | ✓ | ✓ |  |
| N-5-AOA | 512.7 | C_25_H_48_N_6_O_5_ | [M + H] + |  | 3 | ✓ |  |  |
| Des mesilate impurity I | 502.6 | C_23_H_46_N_6_O_6_ | [M + H - H_2_O] + |  | 3 | ✓ |  |  |
| IC202D | 501.6 | C_23_H_43_N_5_O_7_ | [M + H] + |  | 3 | ✓ | ✓ | ✓ |
| 1k2v | 495.6 | C_21_H_45_N_5_O_8_ | [M + Na] + |  | 3 | ✓ | ✓ | ✓ |
| Des H | 460.5 | C_20_H_36_N_4_O_8_ | [M + H] +, [M + H - H_2_O] +,  [M + Na] + |  | 2 | ✓ | ✓ | ✓ |
| N-5-AHO | 444.5 | C_20_H_36_N_4_O_7_ | [M + H] +, [M + H - H_2_O] + |  | 3 | ✓ | ✓ | ✓ |
| DTCTC | 428.5 | C_20_H_36_N_4_O_6_ | [M + H - H_2_O] + |  | 3 | ✓ |  |  |
| N-5-AHHO | 402.5 | C_18_H_34_N_4_O_6_ | [M + H] + |  | 3 | ✓ | ✓ | ✓ |
| N-BAH | 370.5 | C_18_H_34_N_4_O_4_ | [M + H] + |  | 3 | ✓ |  |  |
| Des mesilate impurity B | 360.5 | C_16_H_32_N_4_O_5_ | [M + Na] + |  | 3 | ✓ |  |  |
| N-5-AHAPBA | 259.3 | C_11_H_21_N_3_O_4_ | [M + H] + |  | 3 | ✓ | ✓ | ✓ |

^†^***Des*** *= Desferrioxamine,* ***N*-5-AAOH** *= N-(5-acetamidopentyl)-N'-[5-[[4-[5-[acetyl(hydroxy)amino]pentylamino]-4-oxobutanoyl]-hydroxyamino]pentyl]butanediamide,* ***N-5-AOHOH*** *= 5-[[4-[5-[[4-[5-[Acetyl(hydroxy)amino]pentylamino]-4-oxobutanoyl]-hydroxyamino]pentylamino]-4-oxobutanoyl]-hydroxyamino]pentanoic acid,* ***N-5-AHHHO*** *= N-[5-[acetyl(hydroxy)amino]pentyl]-N'-hydroxy-N'-[5-[[4-[hydroxy-[(5E)-5-hydroxyiminopentyl]amino]-4-oxobutanoyl]amino]pentyl]butanediamide,* ***N-5-AOHH*** *= N-[5-[[4-[5-[acetyl(hydroxy)amino]pentylamino]-4-oxobutanoyl]-hydroxyamino]pentyl]-N'-heptyl-N'-hydroxybutanediamide,* ***N-5-APPPPPS*** *= N-[5-(3-{5-[3-(5-Amino-pentylcarbamoyl)-propionylamino]-pentylcarbamoyl}-propionylamino)-pentyl]-succinamic acid,* ***N-5-NAHAPO*** *= N-[5-[[4-[5-[acetyl(hydroxy)amino]pentylamino]-4-oxobutanoyl]-hydroxyamino]pentyl]-N'-(4-formamidobutyl)butanediamide,* ***N-5-AOHAH*** *= N-[5-[[4-[4-[acetyl(hydroxy)amino]butylamino]-4-oxobutanoyl]-hydroxyamino]pentyl]-N'-(5-aminopentyl)-N'-hydroxybutanediamide,* ***N-5-AOHHP*** *= N-[5-[[4-[5-[acetyl(hydroxy)amino]pentylamino]-4-oxobutanoyl]-hydroxyamino]pentyl]-N'-hydroxy-N'-pentylbutanediamide,* ***N-5-AAO*** *= N'-[5-[acetyl(hydroxy)amino]pentyl]-N-[5-[[4-[5-aminopentyl(hydroxy)amino]-4-oxobutanoyl]amino]pentyl]butanediamide,* ***N-5-AOHA*** *= N'-[5-[[4-[5-[acetyl(hydroxy)amino]pentylamino]-4-oxobutanoyl]-hydroxyamino]pentyl]-N-(5-aminopentyl)butanediamide,* ***N-5-AHOH*** *= N-(5-acetamidopentyl)-N'-[5-[[4-[hexyl(hydroxy)amino]-4-oxobutanoyl]amino]pentyl]-N'-hydroxybutanediamide,* ***N-5-ADOHH*** *= N'-(5-aminopentyl)-N-[5-[[4-[5-(dihydroxyamino)pentylamino]-4-oxobutanoyl]-hydroxyamino]pentyl]-N'-hydroxybutanediamide,* ***N-5-AOHBH*** *= N-[5-[[4-[5-[acetyl(hydroxy)amino]pentylamino]-4-oxobutanoyl]-hydroxyamino]pentyl]-N'-butyl-N'-hydroxybutanediamide,* ***N-5-AHPOPB*** *= N-(5-acetamidopentyl)-N'-[5-[[4-[5-(hydroxyamino)pentylamino]-4-oxobutanoyl]amino]pentyl]butanediamide,* ***N-5-AOA*** *= N'-[5-[[4-(5-acetamidopentylamino)-4-oxobutanoyl]amino]pentyl]-N-(5-aminopentyl)butanediamide,* ***N-5-AHO*** *= N-[5-[acetyl(hydroxy)amino]pentyl]-N'-hydroxy-N'-[5-(4-oxobutanoylamino)pentyl]butanediamide,* ***DTCTC*** *= 1,13-Dihydroxy-1,7,13,19-tetrazacyclotetracosane-2,6,14,18-tetrone,* ***N-5-AHHO*** *= N'-(5-aminopentyl)-N'-hydroxy-N-[5-[hydroxy(4-oxobutanoyl)amino]pentyl]butanediamide,* ***N-BAH*** *= N,N'-bis(4-acetamidobutyl)hexanediamide,* ***N-5-AHAPBA*** *= N'‑[5‑[acetyl(hydroxy)amino]pentyl]butanediamide.*

**Table S8:** The commercial origin of the ingredients utilized in the preparation of the culture medium and the chemical standards employed for Tier 1 mass feature annotation.

| **Compound name** | **CAS** | **Catalogue** | **Supplier** |
| --- | --- | --- | --- |
| D-Glucose monohydrate | 77938-63-7 | 6887.5 | Carl Roth GmbH + Co. KG |
| Yeast Extract | 8013-01-2 | 2363.7 | Carl Roth GmbH + Co. KG |
| Malt extract | 8002-48-0 | X976.2 | Carl Roth GmbH + Co. KG |
| Ethanol, LC-MS grade | 64-17-5 | 1HP8.2 | Carl Roth GmbH + Co. KG |
| Desferrioxamine B | 70-51-9 | Y0001937 | Sigma-Aldrich Chemie GmbH |
| Desferrioxamine E | 26605-16-3 | AG-CN2-0150-M001 | Adipogen Life Sciences |

**Table S9:** The inoculation plan for each run utilizing the 48 FP, with the applied agitation speed, ethanol concentration and sampling day

|  | **1** | **2** | **3** | **4** | **5** | **6** | **7** | **8** |
| --- | --- | --- | --- | --- | --- | --- | --- | --- |
| **A** | **1200 rpm + 3% v/v EtOH**  100 µL Inoculum + 870 µL GYM-Medium + 30 µL Ethanol  Sampling on day 0 | | **1200 rpm + 0% v/v EtOH** 100 µL Inoculum  + 900 µL GYM-Medium  Sampling on day 5 | | **1300 rpm + 0% v/v EtOH** 100 µL Inoculum  + 900 µL GYM-Medium  Sampling on day 5 | | **1400 rpm + 0% v/v EtOH** 100 µL Inoculum  + 900 µL GYM-Medium  Sampling on day 5 | |
| **B** | **1200 rpm + 3% v/v EtOH**  100 µL Inoculum + 870 µL GYM-Medium + 30 µL Ethanol  Sampling on day 1 | | **1200 rpm + 1% v/v EtOH**  100 µL Inoculum + 890 µL GYM-Medium + 10 µL Ethanol  Sampling on day 5 | | **1300 rpm + 1% v/v EtOH**  100 µL Inoculum + 890 µL GYM-Medium + 10 µL Ethanol  Sampling on day 5 | | **1400 rpm + 1% v/v EtOH**  100 µL Inoculum + 890 µL GYM-Medium + 10 µL Ethanol  Sampling on day 5 | |
| **C** | **1200 rpm + 3% v/v EtOH**  100 µL Inoculum + 870 µL GYM-Medium + 30 µL Ethanol  Sampling on day 2 | | **1200 rpm + 2% v/v EtOH**  100 µL Inoculum + 880 µL GYM-Medium + 20 µL Ethanol  Sampling on day 5 | | **1300 rpm + 2% v/v EtOH**  100 µL Inoculum + 880 µL GYM-Medium + 20 µL Ethanol  Sampling on day 5 | | **1400 rpm + 2% v/v EtOH**  100 µL Inoculum + 880 µL GYM-Medium + 20 µL Ethanol  Sampling on day 5 | |
| **D** | **1200 rpm + 3% v/v EtOH**  100 µL Inoculum + 870 µL GYM-Medium + 30 µL Ethanol  Sampling on day 3 | | **1200 rpm + 4% v/v EtOH**  100 µL Inoculum + 860 µL GYM-Medium + 40 µL Ethanol  Sampling on day 5 | | **1300 rpm + 3% v/v EtOH**  100 µL Inoculum + 870 µL GYM-Medium + 30 µL Ethanol  Sampling on day 5 | | **1400 rpm + 3% v/v EtOH**  100 µL Inoculum + 870 µL GYM-Medium + 30 µL Ethanol  Sampling on day 5 | |
| **E** | **1200 rpm + 3% v/v EtOH**  100 µL Inoculum + 870 µL GYM-Medium + 30 µL Ethanol  Sampling on day 4 | | **1200 rpm + 5% v/v EtOH**  100 µL Inoculum + 850µL GYM-Medium + 50 µL Ethanol  Sampling on day 5 | | **1300 rpm + 4% v/v EtOH**  100 µL Inoculum + 860 µL GYM-Medium + 40 µL Ethanol  Sampling on day 5 | | **1400 rpm + 4% v/v EtOH**  100 µL Inoculum + 860 µL GYM-Medium + 40 µL Ethanol  Sampling on day 5 | |
| **F** | **1200 rpm + 3% v/v EtOH**  100 µL Inoculum + 870 µL GYM-Medium + 30 µL Ethanol  Sampling on day 5 | | **-** | | **1300 rpm + 5% v/v EtOH**  100 µL Inoculum + 850µL GYM-Medium + 50 µL Ethanol  Sampling on day 5 | | **1400 rpm + 5% v/v EtOH**  100 µL Inoculum + 850µL GYM-Medium + 50 µL Ethanol  Sampling on day 5 | |

|  | **1** | **2** | **3** | **4** | **5** | **6** | **7** | **8** |
| --- | --- | --- | --- | --- | --- | --- | --- | --- |
| **A** | **800 rpm + 0% v/v EtOH** 100 µL Inoculum  + 900 µL GYM-Medium  Sampling on day 5 | | **900 rpm + 0% v/v EtOH** 100 µL Inoculum  + 900 µL GYM-Medium  Sampling on day 5 | | **1000 rpm + 0% v/v EtOH** 100 µL Inoculum  + 900 µL GYM-Medium  Sampling on day 5 | | **1100 rpm + 0% v/v EtOH** 100 µL Inoculum  + 900 µL GYM-Medium  Sampling on day 5 | |
| **B** | **800 rpm + 1% v/v EtOH**  100 µL Inoculum + 890 µL GYM-Medium + 10 µL Ethanol  Sampling on day 5 | | **900 rpm + 1% v/v EtOH**  100 µL Inoculum + 890 µL GYM-Medium + 10 µL Ethanol  Sampling on day 5 | | **1000 rpm + 1% v/v EtOH**  100 µL Inoculum + 890 µL GYM-Medium + 10 µL Ethanol  Sampling on day 5 | | **1100 rpm + 1% v/v EtOH**  100 µL Inoculum + 890 µL GYM-Medium + 10 µL Ethanol  Sampling on day 5 | |
| **C** | **800 rpm + 2% v/v EtOH**  100 µL Inoculum + 880 µL GYM-Medium + 20 µL Ethanol  Sampling on day 5 | | **900 rpm + 2% v/v EtOH**  100 µL Inoculum + 880 µL GYM-Medium + 20 µL Ethanol  Sampling on day 5 | | **1000 rpm + 2% v/v EtOH**  100 µL Inoculum + 880 µL GYM-Medium + 20 µL Ethanol  Sampling on day 5 | | **1100 rpm + 2% v/v EtOH**  100 µL Inoculum + 880 µL GYM-Medium + 20 µL Ethanol  Sampling on day 5 | |
| **D** | **800 rpm + 3% v/v EtOH**  100 µL Inoculum + 870 µL GYM-Medium + 30 µL Ethanol  Sampling on day 5 | | **900 rpm + 3% v/v EtOH**  100 µL Inoculum + 870 µL GYM-Medium + 30 µL Ethanol  Sampling on day 5 | | **1000 rpm + 3% v/v EtOH**  100 µL Inoculum + 870 µL GYM-Medium + 30 µL Ethanol  Sampling on day 5 | | **1100 rpm + 3% v/v EtOH**  100 µL Inoculum + 870 µL GYM-Medium + 30 µL Ethanol  Sampling on day 5 | |
| **E** | **800 rpm + 4% v/v EtOH**  100 µL Inoculum + 860 µL GYM-Medium + 40 µL Ethanol  Sampling on day 5 | | **900 rpm + 4% v/v EtOH**  100 µL Inoculum + 860 µL GYM-Medium + 40 µL Ethanol  Sampling on day 5 | | **1000 rpm + 4% v/v EtOH**  100 µL Inoculum + 860 µL GYM-Medium + 40 µL Ethanol  Sampling on day 5 | | **1100 rpm + 4% v/v EtOH**  100 µL Inoculum + 860 µL GYM-Medium + 40 µL Ethanol  Sampling on day 5 | |
| **F** | **800 rpm + 5% v/v EtOH**  100 µL Inoculum + 850µL GYM-Medium + 50 µL Ethanol  Sampling on day 5 | | **900 rpm + 5% v/v EtOH**  100 µL Inoculum + 850µL GYM-Medium + 50 µL Ethanol  Sampling on day 5 | | **1000 rpm + 5% v/v EtOH**  100 µL Inoculum + 850µL GYM-Medium + 50 µL Ethanol  Sampling on day 5 | | **1100 rpm + 5% v/v EtOH**  100 µL Inoculum + 850µL GYM-Medium + 50 µL Ethanol  Sampling on day 5 | |

**Table S10:** List of detected metabolic features (MFs) from the duplicate stirred-tank bioreactor runs supplemented with 1% ethanol. The table includes the compound name, abbreviation, neutral mass, sum formula, adduct, maximum detected abundance, tier level, information on which duplicate the feature was detected in, and the corresponding PubChem CID. Cultivation was carried out in stirred-tank bioreactor 1 (STR1). A second, independent cultivation in stirred-tank bioreactor 2 (STR2) was performed to investigate the reproducibility of metabolic footprinting results and morphological characteristics.

| **MF (rt_m/z)** | **Name** | **Abbreviations** | **Neutral mass [g/mol]** | **Sum formula** | **Adduct** | **Max abundance** | **Tier** | **Cultivation system**† | **PubChem CID** |
| --- | --- | --- | --- | --- | --- | --- | --- | --- | --- |
| 2.07_345.2482 | ‒ | ‒ | ‒ | ‒ | ‒ | 47502 | 4 | STR 1 | ‒ |
| 2.08_281.0989 | Desferrioxamine B | Des B | 560.7 | C25H48N6O8 | [M + 2H] 2+ | 6654 | 1 | STR 1, STR 2 | 2973 |
| 2.09_356.1305 | ‒ | ‒ | ‒ | ‒ | ‒ | 5189 | 4 | STR 1, STR 2 | ‒ |
| 2.3_268.1633 | ‒ | ‒ | ‒ | ‒ | ‒ | 11310 | 4 | STR 1, STR 2 | ‒ |
| 2.41_467.2531 | L-leucyl-L-prolyl-L-glutaminyl-L-alanyl-glycine | ‒ | 484.5 | C21H36N6O7 | [M + H - H2O] + | 10827 | 3 | STR 1, STR 2 | 11306255 |
| 2.42_467.2598 | L-leucyl-L-prolyl-L-glutaminyl-L-alanyl-glycine | ‒ | 484.5 | C21H36N6O7 | [M + H - H2O] + | 10827 | 3 | STR 1, STR 2 | 11306255 |
| 2.43_289.6544 | ‒ | ‒ | ‒ | ‒ | ‒ | 4113 | 4 | STR 1, STR 2 | ‒ |
| 2.45_415.1591 | 3-(3-(3,4-Dihydroxy-5-(6-hydroxy-9H-purin-9-yl)tetrahydrofuran-2-yl)propanoyl)benzoic acid | ‒ | 414.4 | C19H18N4O7 |  | 11164 | 2 | STR 1, STR 2 | 135613041 |
| 2.48_394.2046 | ‒ | ‒ | ‒ | ‒ | ‒ | 7396 | 4 | STR 1, STR 2 | ‒ |
| 2.54_166.0843 | ‒ | ‒ | ‒ | ‒ | ‒ | 13438 | 4 | STR 1, STR 2 | ‒ |
| 2.58_408.2194 | ‒ | ‒ | ‒ | ‒ | ‒ | 12542 | 4 | STR 1, STR 2 | ‒ |
| 2.67_270.1795 | ‒ | ‒ | ‒ | ‒ | ‒ | 3996 | 4 | STR 1, STR 2 | ‒ |
| 2.75_414.1374 | ‒ | ‒ | ‒ | ‒ | ‒ | 30141 | 4 | STR 1, STR 2 | ‒ |
| 2.76_369.1987 | ‒ | ‒ | ‒ | ‒ | ‒ | 46983 | 4 | STR 1, STR 2 | ‒ |
| 2.77_200.0907 | ‒ | ‒ | ‒ | ‒ | ‒ | 18577 | 4 | STR 1, STR 2 | ‒ |
| 2.81_227.1368 | ‒ | ‒ | ‒ | ‒ | ‒ | 37826 | 4 | STR 1, STR 2 | ‒ |
| 2.81_180.099 | ‒ | ‒ | ‒ | ‒ | ‒ | 25888 | 4 | STR 1, STR 2 | ‒ |
| 2.83_518.3158 | 1k2v | ‒ | 495.6 | C21H45N5O8 | [M + Na] + | 45630 | 3 | STR 1, STR 2 | 23585928 |
| 2.83_259.6635 | ‒ | ‒ | ‒ | ‒ | ‒ | 21536 | 4 | STR 1, STR 2 | ‒ |
| 2.83_200.0909 | ‒ | ‒ | ‒ | ‒ | ‒ | 18577 | 4 | STR 1, STR 2 | ‒ |
| 2.84_273.1848 | N-[5-[[4-[5-[acetyl(hydroxy)amino]pentylamino]-4-oxobutanoyl]-hydroxyamino]pentyl]-N'-hydroxy-N'-pentylbutanediamide | N-5-AOHHP | 545.7 | C25H47N5O8 | [M + 2H] 2+ | 491420 | 3 | STR 1, STR 2 | 137444661 |
| 2.84_289.1795 | N-Hydroxydesferrioxamine B | Hydroxy-Des B | 576.7 | C25H48N6O9 | [M + 2H] 2+ | 14531 | 3 | STR 1, STR 2 | 44149952 |
| 2.84_545.3675 | N'-[5-[acetyl(hydroxy)amino]pentyl]-N-[5-[[4-[5-aminopentyl(hydroxy)amino]-4-oxobutanoyl]amino]pentyl]butanediamide | N-5-AAO | 544.7 | C25H48N6O7 |  | 136437 | 3 | STR 1, STR 2 | 60019158 |
| 2.84_518.3234 | 1k2v | ‒ | 495.6 | C21H45N5O8 | [M + Na] + | 45630 | 3 | STR 1, STR 2 | 23585928 |
| 2.84_577.3532 | N-Hydroxy-desferrioxamine B | Hydroxy-Des B | 576.7 | C25H48N6O9 | ‒ | 17448 | 3 | STR 1, STR 2 | 44149952 |
| 2.85_545.3599 | N'-[5-[acetyl(hydroxy)amino]pentyl]-N-[5-[[4-[5-aminopentyl(hydroxy)amino]-4-oxobutanoyl]amino]pentyl]butanediamide | N-5-AAO | 544.7 | C25H48N6O7 | ‒ | 121649 | 3 | STR 1, STR 2 | 60019158 |
| 2.85_517.3256 | N'-(5-aminopentyl)-N-[5-[[4-[5-(dihydroxyamino)pentylamino]-4-oxobutanoyl]-hydroxyamino]pentyl]-N'-hydroxybutanediamide | N-5-ADOHH | 534.6 | C23H46N6O8 | [M + H - H2O] + | 32302 | 3 | STR 1, STR 2 | 163123947 |
| 2.87_517.3319 | ‒ | ‒ | ‒ | ‒ | ‒ | 32302 | 4 | STR 1, STR 2 | ‒ |
| 2.88_227.1374 | ‒ | ‒ | ‒ | ‒ | ‒ | 37826 | 4 | STR 1, STR 2 | ‒ |
| 2.91_314.1663 | ‒ | ‒ | ‒ | ‒ | ‒ | 22007 | 4 | STR 1, STR 2 | ‒ |
| 3.14_208.0578 | ‒ | ‒ | ‒ | ‒ | ‒ | 9818 | 4 | STR 1, STR 2 | ‒ |
| 3.15_376.2064 | ‒ | ‒ | ‒ | ‒ | ‒ | 80083 | 4 | STR 1 | ‒ |
| 3.16_284.1942 | ‒ | ‒ | ‒ | ‒ | ‒ | 4827 | 4 | STR 1, STR 2 | ‒ |
| 3.2_403.2454 | N'-(5-aminopentyl)-N'-hydroxy-N-[5-[hydroxy(4-oxobutanoyl)amino]pentyl]butanediamide | N-5-AHPOPB | 402.5 | C18H34N4O6 | ‒ | 14765 | 3 | STR 1, STR 2 | 89072474 |
| 3.22_403.2546 | ‒ | ‒ | ‒ | ‒ | ‒ | 14765 | 4 | STR 1, STR 2 | ‒ |
| 3.22_461.26 | Desferrioxamine H | Des H | 460.5 | C20H36N4O8 | ‒ | 89087 | 2 | STR 1, STR 2 | 57509383 |
| 3.22_461.251 | Desferrioxamine H | Des H | 460.5 | C20H36N4O8 | ‒ | 23959 | 2 | STR 1, STR 2 | 57509383 |
| 3.29_533.3294 | IC202B | ‒ | 532.6 | C23H44N6O8 | ‒ | 9622 | 3 | STR 1, STR 2 | 9828474 |
| 3.51_243.1311 | ‒ | ‒ | ‒ | ‒ | ‒ | 107392 | 4 | STR 1, STR 2 | ‒ |
|  |  |  |  |  |  |  |  | STR 1, STR 2 |  |
| 3.52_288.6655 | ‒ | ‒ | ‒ | ‒ | ‒ | 180552 | 4 | STR 1 | ‒ |
| 3.52_361.2427 | L-lysyl-L-threonyl-L-isoleucine | ‒ | 360.45 | C16H32N4O5 | ‒ | 22296 | 3 | STR 1, STR 2 | 145456782 |
| 3.52_443.2497 | Desferrioxamine H | Des H | 460.5 | C20H36N4O8 | [M +H - H2O] + | 88071 | 2 | STR 1, STR 2 | 57509383 |
| 3.53_576.3263 | 5-[[4-[5-[[4-[5-[Acetyl(hydroxy)amino]pentylamino]-4-oxobutanoyl]-hydroxyamino]pentylamino]-4-oxobutanoyl]-hydroxyamino]pentanoic acid | N-5-AOHOH | 575.7 | C25H45N5O10 | ‒ | 281908 | 2 | STR 1, STR 2 | 14459425 |
| 3.6_623.331 | ‒ | ‒ | ‒ | ‒ | ‒ | 13752 | 4 | STR 1, STR 2 | ‒ |
| 3.6_301.1801 | Desferrioxamine E | Des E | 600.7 | C27H48N6O9 | [M + 2H] 2+ | 61343 | 1 | STR 1, STR 2 | 161532 |
|  |  |  |  |  |  |  |  | STR 1, STR 2 |  |
| 3.6_601.3561 | Desferrioxamine E | Des E | 600.7 | C27H48N6O9 | ‒ | 113281 | 1 | STR 1, STR 2 | 161532 |
| 3.6_623.3372 | Desferrioxamine E | Des E | 600.7 | C27H48N6O9 | [M + Na] + | 13752 | 1 | STR 1, STR 2 | 161532 |
| 4.1_164.1011 | ‒ | ‒ | ‒ | ‒ | ‒ | 3678 | 4 | STR 1, STR 2 | ‒ |
| 4.22_164.1058 | ‒ | ‒ | ‒ | ‒ | ‒ | 7980 | 4 | STR 1, STR 2 | ‒ |
| 4.22_400.2874 | ‒ | ‒ | ‒ | ‒ | ‒ | 8874 | 4 | STR 1, STR 2 | ‒ |
| 6.29_532.2869 | ‒ | ‒ | ‒ | ‒ | ‒ | 2168 | 4 | STR 2 | ‒ |
| 6.72_499.7772 | ‒ | ‒ | ‒ | ‒ | ‒ | 63598 | 4 | STR 1, STR 2 | ‒ |
| 6.72_490.77 | ‒ | ‒ | ‒ | ‒ | ‒ | 31429 | 4 | STR 1, STR 2 | ‒ |
| 7.45_238.5541 | ‒ | ‒ | ‒ | ‒ | ‒ | 4289 | 4 | STR 2 | ‒ |
| 10.18_546.4782 | ‒ | ‒ | ‒ | ‒ | ‒ | 3814 | 4 | STR 1, STR 2 | ‒ |

^†^*STR 1 = Stirred tank bioreactor duplicate 1 = STR 2 = Stirred tank bioreactor duplicate 2.*


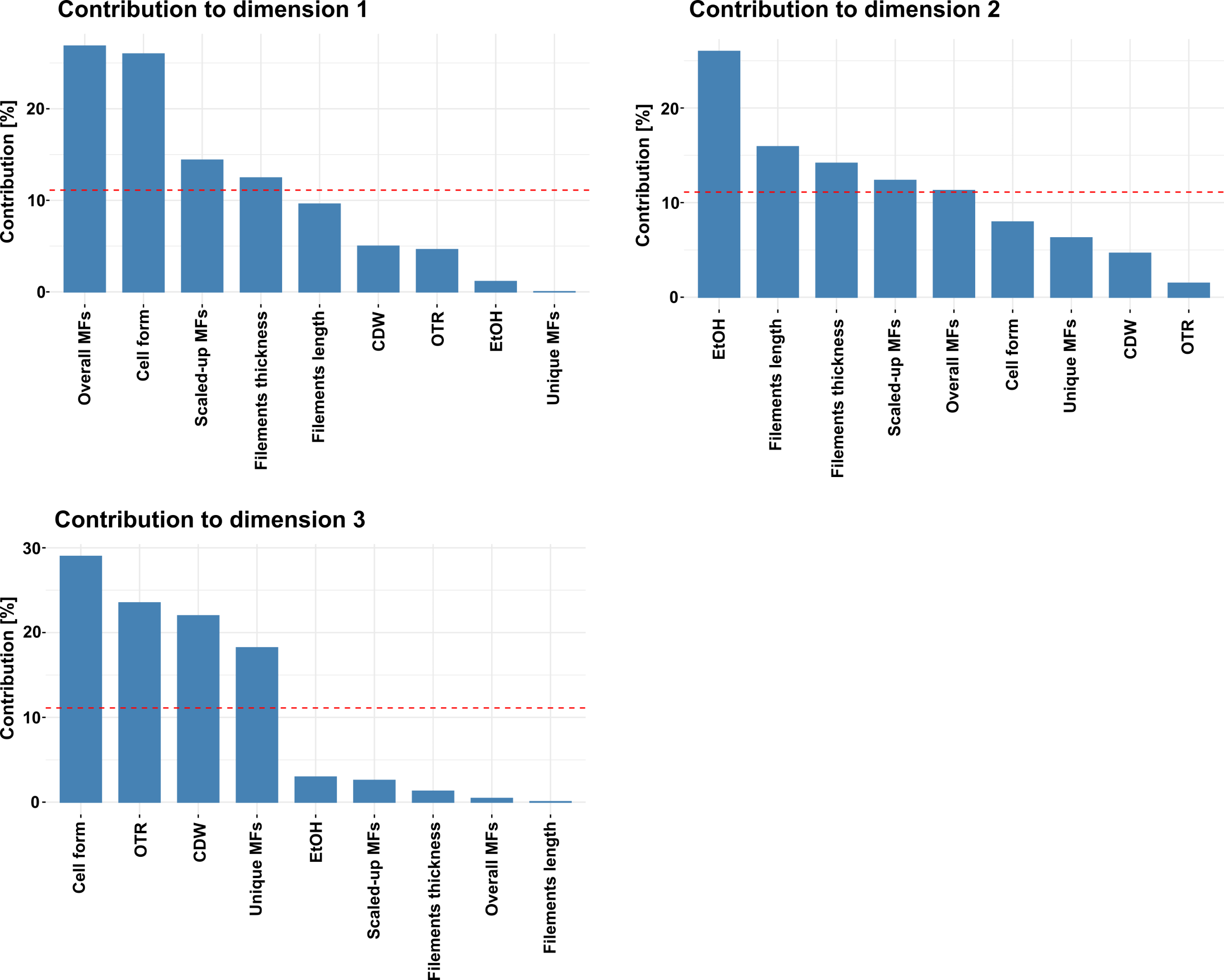


**Figure S1:** This figures depicts the contributions of both quantitative and qualitative variables (theoretical OTR = OTR, cell dry weight = CDW, ethanol concentrations = EtOH, the total number of MFs = Overall MFs, the unique MFs and the MFs detectable in STR = Scaled-up MFs, filament lengths, filament thickness and cell form) to the significant dimensions one, two, and three. The red dashed line represents the expected average value assuming uniform contributions.


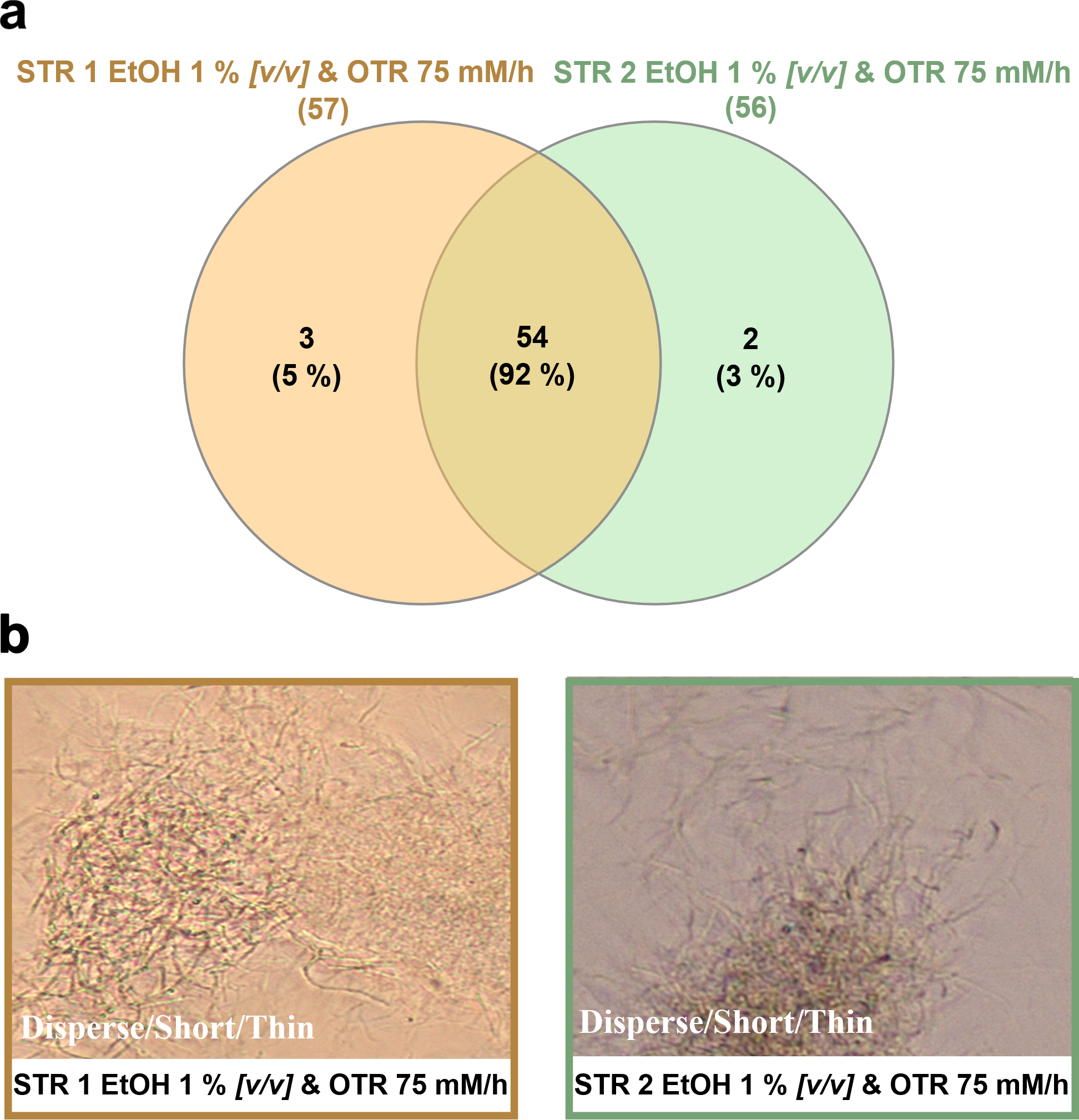


**Figure S2:** **a** Venn diagram illustrating the overlap of detected MFs at the end of the cultivation period, obtained in duplicate STR runs supplemented with 1% ethanol. Stirred-tank bioreactor 1 (STR1) was used for the main cultivation experiment and stirred-tank bioreactor 2 (STR2) was operated as an independent biological replicate to verify the reproducibility of both metabolic footprinting data and morphological observations. Metabolic footprint analysis was performed using an untargeted metabolomics workflow on supernatant extracts. An MF is defined as a detected ion grouped with its corresponding retention time (rt_m/z). Only MFs with an abundance greater than 1000 and available MS/MS fragmentation were considered. Features originating from pre-culture or medium-only controls were excluded. b Representative micrographs captured at the end of the cultivation period (10× magnification), showing the predominant morphology observed in the STR.


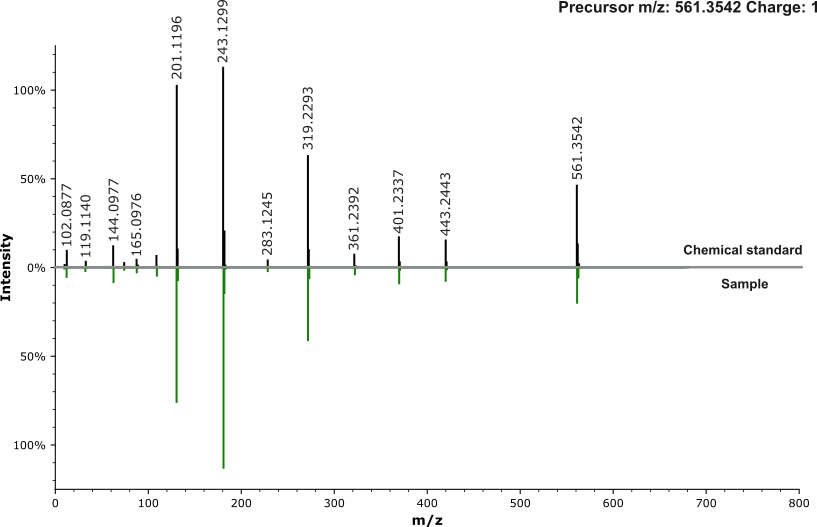


**Figure S3:** The chemical standard annotation for desferrioxamine B. The top section displays the MS/MS fragmentation pattern from the chemical standard, while the bottom section shows the MS/MS acquired in this study.
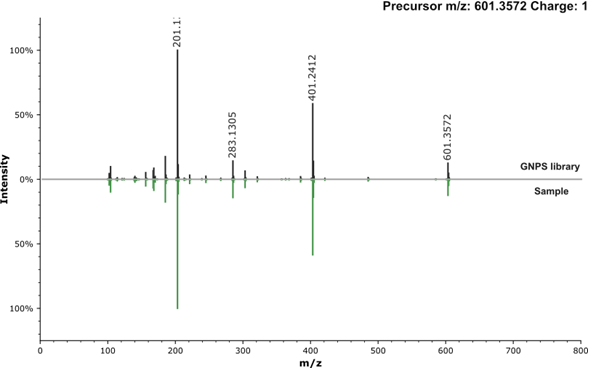


**Figure S4:** A GNPS spectral library hit for desferrioxamine Et1. The MSMS fragmentation pattern from the spectral library is displayed on the top, and the MSMS acquired in this study is displayed on the bottom.
